# Identification of breast cancer associated variants that modulate transcription factor binding

**DOI:** 10.1371/journal.pgen.1006761

**Published:** 2017-09-28

**Authors:** Yunxian Liu, Ninad M. Walavalkar, Mikhail G. Dozmorov, Stephen S. Rich, Mete Civelek, Michael J. Guertin

**Affiliations:** 1 Department of Biochemistry and Molecular Genetics, University of Virginia, Charlottesville, Virginia, United States of America; 2 Department of Biostatistics, Virginia Commonwealth University, Richmond, Virginia, United States of America; 3 Center for Public Health Genomics, University of Virginia, Charlottesville, Virginia, United States of America; 4 Department of Biomedical Engineering, University of Virginia, Charlottesville, Virginia, United Statess of America; Case Western Reserve University School of Medicine, UNITED STATES

## Abstract

Genome-wide association studies (GWAS) have discovered thousands loci associated with disease risk and quantitative traits, yet most of the variants responsible for risk remain uncharacterized. The majority of GWAS-identified loci are enriched for non-coding single-nucleotide polymorphisms (SNPs) and defining the molecular mechanism of risk is challenging. Many non-coding causal SNPs are hypothesized to alter transcription factor (TF) binding sites as the mechanism by which they affect organismal phenotypes. We employed an integrative genomics approach to identify candidate TF binding motifs that confer breast cancer-specific phenotypes identified by GWAS. We performed *de novo* motif analysis of regulatory elements, analyzed evolutionary conservation of identified motifs, and assayed TF footprinting data to identify sequence elements that recruit TFs and maintain chromatin landscape in breast cancer-relevant tissue and cell lines. We identified candidate causal SNPs that are predicted to alter TF binding within breast cancer-relevant regulatory regions that are in strong linkage disequilibrium with significantly associated GWAS SNPs. We confirm that the TFs bind with predicted allele-specific preferences using CTCF ChIP-seq data. We used The Cancer Genome Atlas breast cancer patient data to identify ANKLE1 and ZNF404 as the target genes of candidate TF binding site SNPs in the 19p13.11 and 19q13.31 GWAS-identified loci. These SNPs are associated with the expression of ZNF404 and ANKLE1 in breast tissue. This integrative analysis pipeline is a general framework to identify candidate causal variants within regulatory regions and TF binding sites that confer phenotypic variation and disease risk.

## Author summary

The promise of effective personalized medicine is dependent upon the ability to identify genetic variants in the population that influence disease risk and then use this information to accurately predict the likelihood of disease incidence for individual patients. High-risk individuals may be entered into clinical trails, pre-clinical intervention strategies, or increased frequency of screening to detect early disease onset. However, the contribution of any one genetic variant to increase disease susceptibility is typically small, with many potential causal variants in the genomic region associated with risk. Therefore, it is important to understand the biological mechanisms by which the variants within a genetic region influence disease susceptibility by refining the set of all variants to those that are highly plausible to be causal. Herein, we describe a method to integrate molecular genomics data with genetic epidemiological data to inform on the underlying molecular mechanisms that influence breast cancer risk. This approach identifies the important transcription factors that directly regulate gene expression to modulate disease susceptibility.

## Introduction

Genome-wide association studies (GWAS) have identified more than 90 genomic loci and common genetic variants associated with breast cancer [1–5]. The single nucleotide polymorphisms (SNPs) associated with breast cancer have been shown to be enriched in DNA regulatory regions [6, 7], with few residing in coding regions of genes. The mechanisms by which most of these variants contribute to breast cancer biology remain unknown [8–11]. The effects of putative causal non-coding SNPs are challenging to interpret as they may alter transcription factor (TF) binding sites [12], lncRNA structure [13], splicing [14], transcription start or termination signals, or DNA shape [15]. Non-coding SNPs that alter TF binding sites are the most easily interpreted because they have the potential to modulate gene expression to mediate their effects on disease risk [16]. Therefore, it is possible to identify putative causal SNPs by focusing on those that alter TF binding sites in breast tissue.

TF dysregulation is a hallmark of many cancers [17, 18]. Genes encoding TFs in tumor cells are often amplified, deleted, rearranged via chromosomal translocation, or subjected to point mutations that result in a gain- or loss-of-function [18]. For example, transcriptional amplification of c-Myc reduces rate-limiting constraints for tumor cell growth and proliferation; high c-Myc expression correlates with tumor aggression and poor clinical outcome [19]. Estrogen receptor (ER) is a TF that regulates cell proliferation, which is the defining feature of luminal breast cancers [20]. Identifying the full set of TFs that function within a cell type remains a challenge.

Enzymatic accessibility assays identify open chromatin in the genome, which is an indirect measure of regulatory element activity and TF binding events [21–25]. TFs that directly or indirectly recruit cofactors, such as histone modifiers and nucleosome remodelers, recognize sequence motifs that are enriched in regions of open chromatin characterized by active histone marks and enzymatic hypersensitivity peaks [26, 27]. These cofactors maintain the chromatin structure at regulatory elements. Therefore, one strategy for identifying the functional TFs in a cell is to query the sequence underlying accessible regions for over-represented motifs [28–31]. Alternatively, depletion of signal within hypersensitive regions (footprints) [30, 31] coupled to motif analysis can be used to infer TF binding. The reliance on hypersensitivity footprinting to define a near-comprehensive set of TF motifs is limiting because many TFs do not have footprints for biological reasons [31] and the enzymes exhibit sequence-specificity that can be misinterpreted as footprint signatures [31–33]. These methods strictly identify motifs that are over-represented within regions of open chromatin, but families of TFs often contain paralogous DNA binding domains and thus recognize indistinguishable sequence motifs. One can directly measure the expression of TFs to identify putative functional TFs among related TFs [34]; however, expression of a TF is an imperfect proxy for TF function. For example, many nuclear receptors are not transcriptionally functional in the absence of ligand regardless of expression levels. Here we propose an approach that integrates open chromatin genomic data and gene expression data to identify candidate TFs that are functional in breast cancer-relevant cells.

Expression quantitative trait loci (eQTL) analysis identifies genetic variants that correlate with gene expression differences in a population. eQTL analysis complements genetic association data by predicting causal genes whose expression differences dictate organismal phenotypes [35, 36]. The gene that is responsible for a trait may be located relatively far from the GWAS associated SNPs, as the causal SNPs may modulate TF binding and TFs can act distally to regulate gene transcription. In these cases, preferential binding of a TF to one allele causes differential regulation of gene expression to confer the phenotype [37]. Several studies have provided evidence of causal relationships for gene expression mediating the association between GWAS SNPs and traits [16, 38]. Phenotype-associated SNPs are enriched for eQTLs, suggesting that eQTL analysis can enhance discovery of causal genes associated with complex phenotypes [39, 40].

The goal of this study is to gain mechanistic insight into how breast cancer susceptibility alleles confer disease risk. First we identify sequence motifs in breast-relevant tissue and cell lines that are enriched within enzymatic hypersensitive sites and support their functional role with evolutionary conservation analysis and gene expression data. Next we identify breast cancer risk alleles that are predicted to modulate TF binding and we perform eQTL analysis to find candidate causal risk genes.

## Results

### Identification of functional TFs within breast-derived cells and tissues

**Fig 1:**
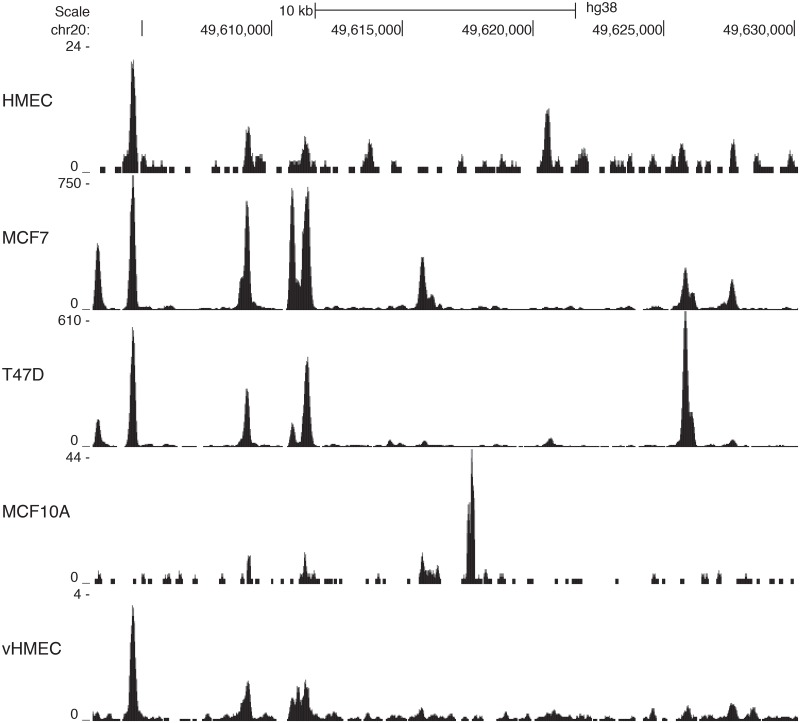
DNase-seq and ATAC-seq quantify differential open chromatin in breast cancer-relevant tissue and cell lines. We show smoothed DNase-seq and ATAC-seq tracks at a locus on chromosome 20. MCF7, T47D, HMEC and vHMEC DNase-seq data share several chromatin accessibility regions and MCF10A ATAC-seq data identifies a peak of differential chromatin accessibility that is distinct from the other data sets.

Regulatory regions are bound by a milieu of protein complexes and regulatory nucleic acids, such as transcription factors, histone modifiers, nucleosome remodelers, and lncRNA. Sequence-specific TFs are directly or indirectly responsible for the recruitment of downstream transcriptional modifiers in a sequence-dependent manner. Therefore, we sought to identify all TFs that bind within open chromatin in breast cancer-relevant cells and tissues. We performed ATAC-seq in a mammary epithelial cell line (MCF10A) to complement publicly available DNase-seq data from Encyclopedia of DNA Elements (ENCODE) [41] and the Roadmap Epigenomics project [42]. We quantified open chromatin and identified regulatory elements genome-wide in five breast cancer-relevant cell lines and breast tissue: MCF7 cells, MCF10A cells, T47D cells, cultured human mammary epithelial cells (HMEC), and primary breast variant HMECs (vHMEC). Many regions of chromatin accessibility are shared between the cell lines, although the degree of accessibility for a region can vary between cell types (Fig 1). Enzymatic accessibility coverage (peaks) [28, 29] or depletions of signal in hypersensitive region (footprints) [30, 31] coupled to motif analysis are routinely used to identify TFs that maintain open chromatin structure.

**Fig 2:**
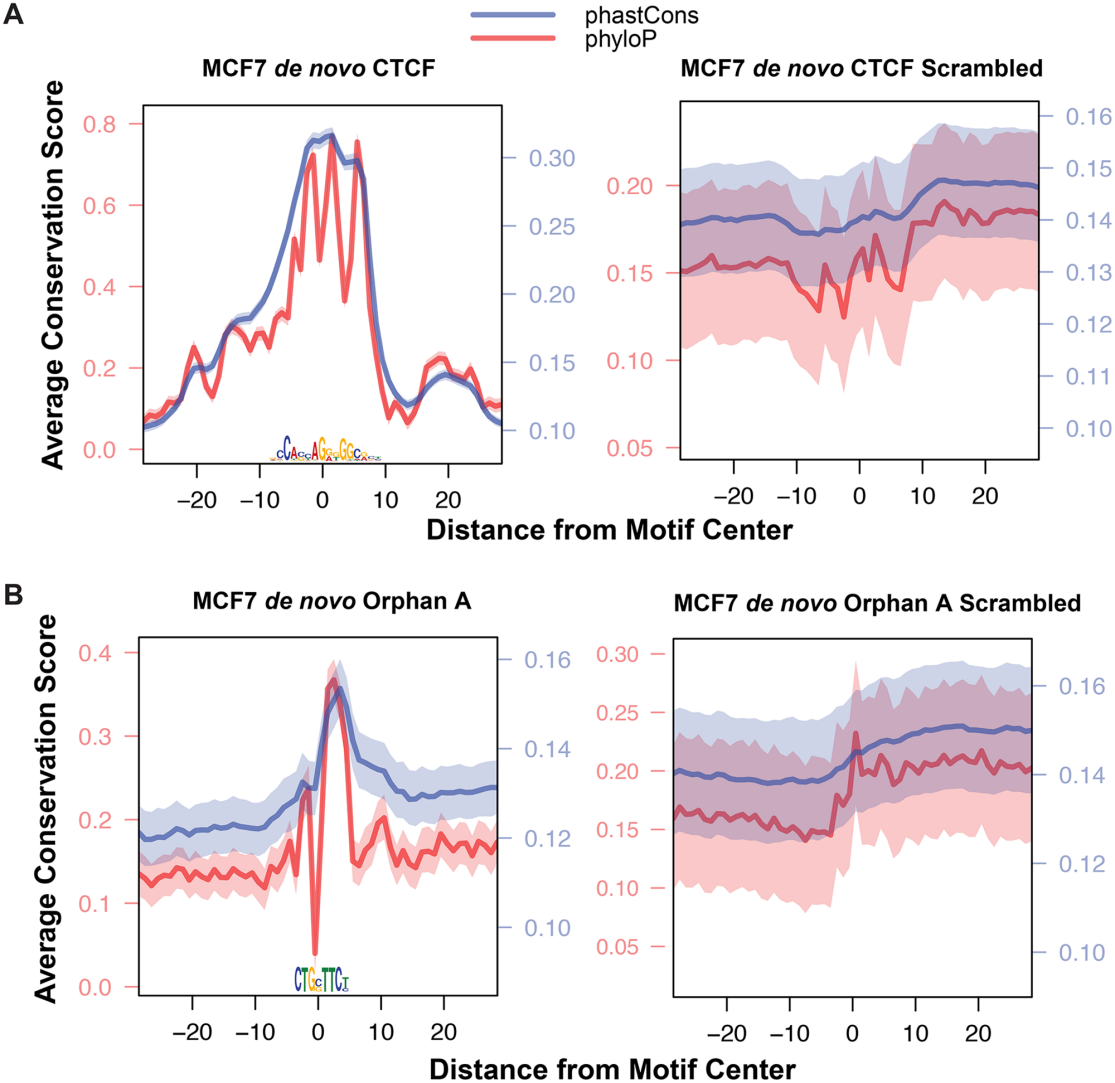
*De novo* identified motifs within open chromatin are evolutionarily conserved. (A) CTCF motifs within hypersensitive sites are, on average, conserved; note the peak of phastCons (blue trace) and pyhloP (red trace) intensity at the motif compared to the flanking region. The right panel is an average of 20 scrambled CTCF weight matrices. We do not observe any conservation peak after scrambling. (B) A novel orphan motif, which does not have a known cognate TF partner, is also evolutionarily conserved within open chromatin regions.

**Fig 3:**
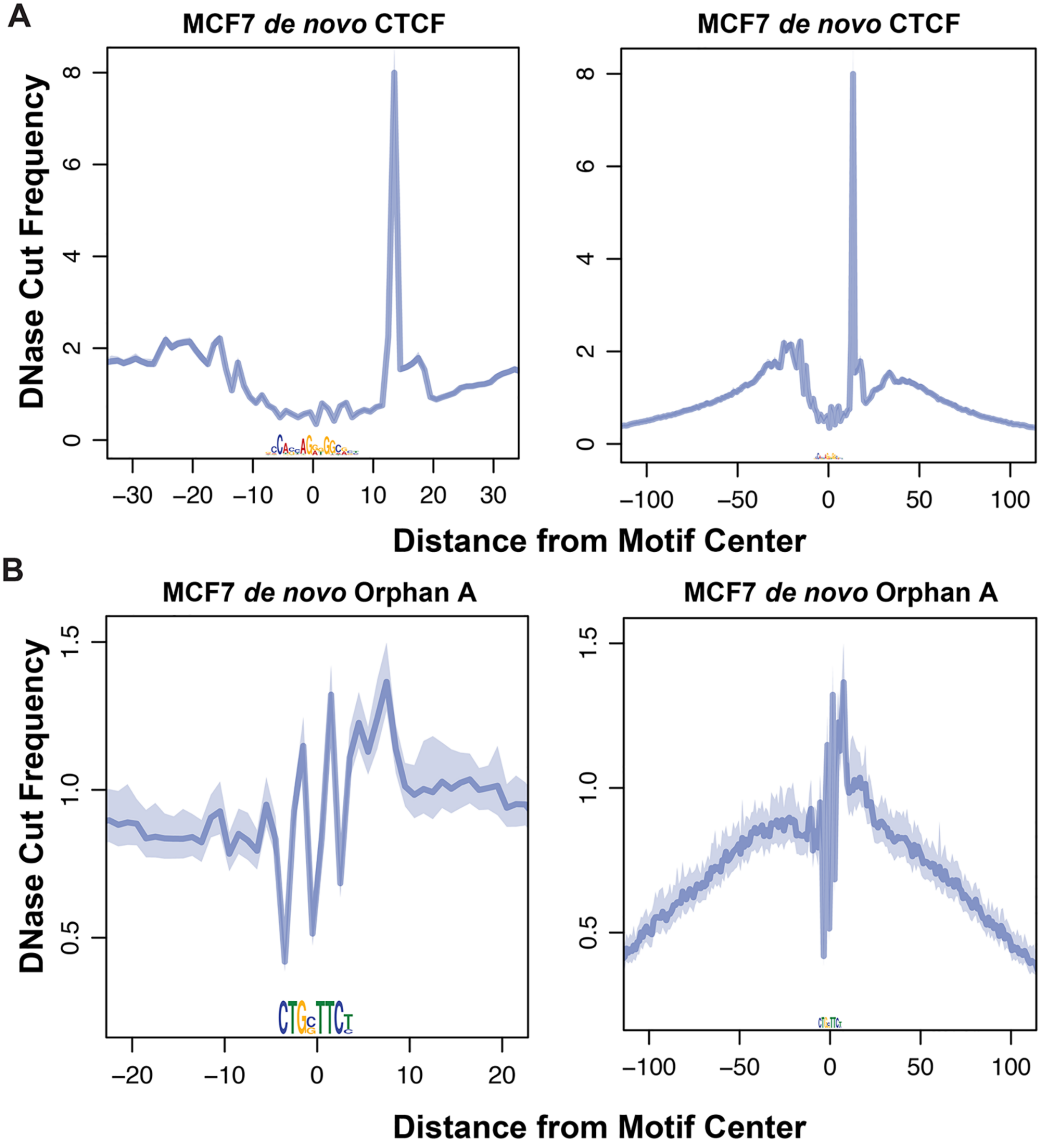
Composite footprints and directional patterns of enzyme accessibility indicate TF occupancy. (A) The CTCF motif exhibits one of the most striking composite footprints and directional patterns of accessibility among sequence-specific binding proteins. (B) We do not observe a composite footprint for this orphan motif; however, the enzyme accessibility pattern around this motif is directional, with higher degree of cleavage downstream from the motif.

We performed iterative rounds of *de novo* motif analysis using the sequence underlying enzymatic hypersensitivity peaks to identify overrepresented TF recognition sites in each data set (S1 File). All but one of the motifs we identified were previously characterized and described in databases [43–47]. These motifs found in hypersensitive peaks are, on average, evolutionarily conserved (Fig 2). Additionally, we identified a potential TF recognition sequence that has no known cognate TF binding partner; we refer to this sequence element as an *orphan motif* (Fig 2B). This orphan motif is evolutionarily conserved, as measured by phastCons [48] and phyloP [49] scores, in hypersensitivity peaks. Hypersensitivity footprints result from protection of the DNA by a bound TF [50]; however, approximately half of all TF-bound motifs do not exhibit composite footprints [31, 51] because the TF dissociates during the nuclei isolation procedure. We corrected the DNase data for intrinsic sequence bias [33], but we do not observe a composite footprint for this orphan motif (Fig 3B). However, the hypersensitivity pattern surrounding the motif is not uniform—the region downstream of this orphan motif is more hypersensitive than upstream. This directional pattern of enzyme accessibility is common with many TFs [29], including CTCF (Fig 3A). We hypothesize that this orphan motif is functional and directs chromatin accessibility by serving as a recognition site for an uncharacterized TF.

**Fig 4:**
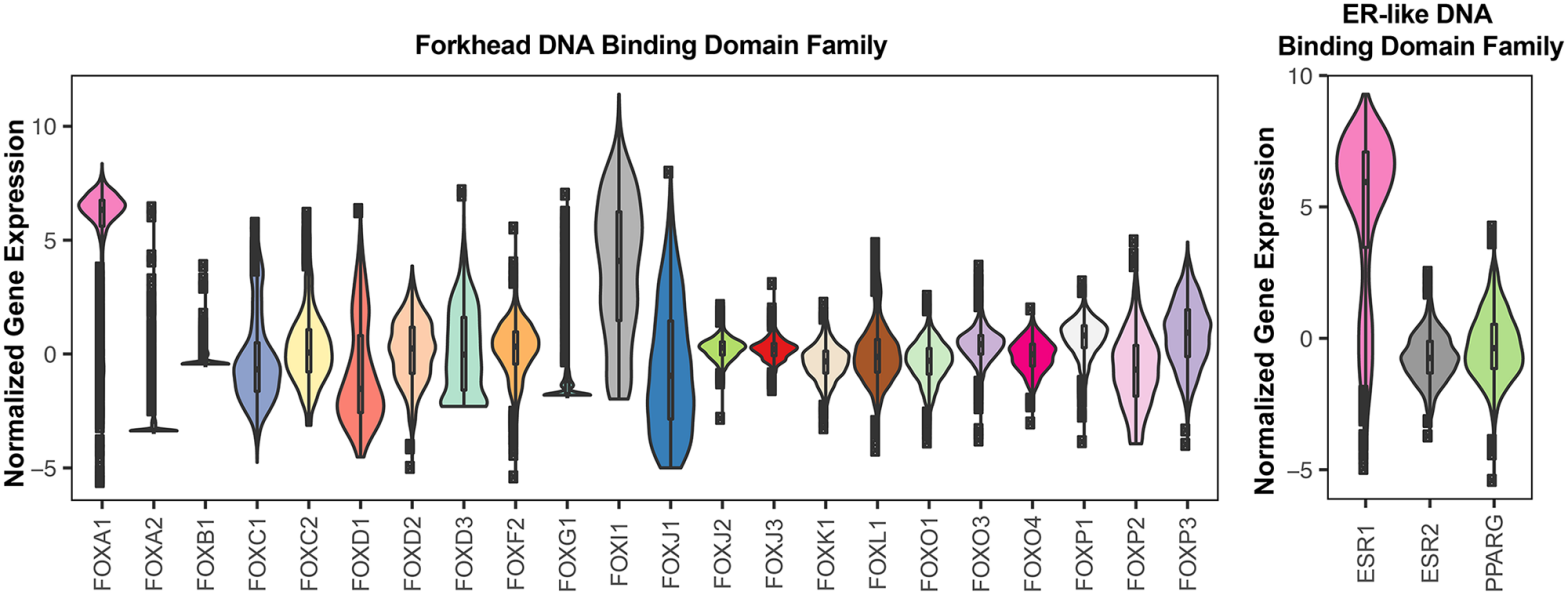
The most highly expressed TFs with paralogous DNA binding domains are most relevant to breast cancer. The relative expression of TFs that recognize the same sequence motif can identify the top candidate functional TFs. FOXA1 and ESR1 are the highest expressed TF in each of their TF families. We quantified gene expression using TCGA breast cancer patient solid tumor samples [57].

In addition to this orphan motif, we identified hundreds of position-specific weight matrices (PSWM) from our exhaustive analyses, many were redundant between cell lines and found in multiple rounds of motif analysis in the same cells. To reduce the complexity of these data, we mapped the comprehensive set of motifs found in MCF7, MCF10A, T47D, HMEC and vHMEC into distinct non-redundant motif families. This operation resulted in identification of 37 sequence motif classes across the five breast cancer-relevant cell lines and tissue (S1 Fig). Many TFs share paralogous DNA binding domains and these TFs often recognize the same sequence motifs. We defined the full set of TFs that recognize each motif by using known TF/sequence interaction data from ChIP-seq [43, 45, 52], protein binding microarrays [53, 54], and SELEX [46, 47] data. We identified 23 TF families in at least two cell lines/breast tissue (Table 1). Fourteen TF families were uniquely found in one cell line or tissue (Table 1). These 37 TF families represent the binding motifs for 235 distinct TFs (Table 1). However, these TFs are not all expressed in breast tissue. We identified the TFs that are most likely candidates for maintaining open chromatin and the gene regulatory expression profiles in breast-relevant cells by examining the relative expression of all of the TFs in each family using TCGA expression data (Fig 4 and S2 Fig). For example, ESR1, ESR2, and PPAR-*γ* contain paralogous DNA binding domains and they recognize indistinguishable sequence elements (S1 Fig). We find that ESR1 is the most highly expressed TF that recognizes the motif (Fig 4). This result is consistent with the biological role of ESR1 in the etiology of breast cancer and breast biology compared to ESR2 and PPAR-*γ*. Similarly, FOXA1 is the most well-charactered TF within the Forkhead Box family of TFs in terms of estrogen signaling and interplay with ER [55]. As expected, we find that FOXA1 is the most highly expressed TF in the family of 22 Forkhead Box TFs (Fig 4). We find that many of these highly expressed TFs correlate with breast cancer survival time in a subtype-specific manner (S3 Fig). Silencing of IRF7 pathways in breast cancer cells promotes breast cancer metastasis, and high expression of the IRF7-regulated genes with breast cancer is associated with prolonged survival [56]. Similarly, we find that high expression of IRF7 is correlated (*P* = 0.029) with positive breast cancer patient outcome in Luminal A subtype (S3 Fig). We find that high expression of BATF (*P* = 0.0035) and TP73 (*P* = 0.0077) is correlated with breast cancer patient survival in HER2+ and Basal-like subtypes, respectively (S3 Fig). Taken together, these data support the notion that TF expression levels may serve as biomarkers of patient outcome.

**Table 1:** Iterative *de novo* motif analysis identified a set of 37 overrepresented motif families within the regulatory elements in MCF7, MCF10A, T47D, HMEC and vHMEC cells. TFs that recognize similar regulatory sequences are clustered into families. Each row contains one TF family and we denote the cell lines/tissues with each identified TF family by check mark.

TF family	MCF7	MCF10A	T47D	HMEC	vHMEC
HNF4A HNF4G NR1H2 NR2C2 NR2F1 NR2F6 PPARG RXRA RXRB RXRG	✓				
BCL6B E2F1 E2F3 E2F4 E2F6 EGR1 KLF1 KLF12 KLF13 KLF14 KLF16 KLF4 KLF5 KLF7 SP1 SP2 SP3 SP4 SP8 ZNF410	✓	✓	✓	✓	✓
ARNT ARNTL BHLHE40 BHLHE41 CLOCK CREB3L2 MAX MITF MITF MLX MLXIPL MYCN NPAS2 RBPJ SREBF1 SREBF2 TCFL5 TFE3 TFEB TFEC USF1 USF2	✓				
ELK1 ELK3 ELK4 ERF ERG ETS1 ETV1 ETV2 ETV3 ETV4 ETV5 ETV6 FEV FLI1 GABPA	✓	✓	✓	✓	✓
MAZ PAX4 RREB1 RUNX1 RUNX2 ZNF263 ZNF281 ZNF740	✓	✓	✓	✓	✓
ATF1 ATF2 ATF3 ATF7 BATF3 CREB1 CREB3 CREB5 JDP2 JUN JUND MAFB XBP1	✓	✓	✓	✓	
ESRRA ESRRB ESRRG NR1H3 NR2F1 NR2F2 NR4A1 NR4A2 NR5A2 RARA RARB RARG RXRA	✓		✓	✓	
SOX10 SOX2 SOX21 SOX3 SOX4 SOX6 SOX9 SRY		✓			
TFAP2A TFAP2B TFAP2C	✓		✓	✓	
NF1 NFIA NFIB NFIC NFIX TLX1	✓		✓	✓	
FOXA1 FOXA2 FOXB1 FOXC1 FOXC1 FOXC2 FOXD1 FOXD2 FOXD3 FOXF2 FOXG1 FOXG1 FOXI1 FOXJ1 FOXJ2 FOXJ2 FOXJ3 FOXJ3 FOXK1 FOXL1 FOXL1 FOXO1 FOXO3 FOXO4 FOXP1 FOXP2 FOXP3	✓		✓	✓	
EHF ELF1 ELF3 ELF4 ELF5 ETS1 SPDEF SPI1		✓			
RFX1 RFX2 RFX3 RFX4 RFX5 RFX7	✓		✓		✓
ZBTB33	✓		✓	✓	✓
CEBPA CEBPB	✓			✓	
IRF1 IRF2 IRF3 IRF4 IRF5 IRF6 IRF7 IRF8 IRF9 PRDM1 STAT1 STAT2			✓		
GRHL1 GRHL2 TFCP2 TFCP2L1	✓		✓	✓	
ZNF143	✓	✓	✓	✓	✓
ATF3 BATF FOS FOSL1 FOSL2 JDP2 JUN JUNB JUND NFAT5 NFATC1 NFATC3 NFE2	✓	✓			
ATF4 DDIT3	✓				
NFYA NFYB	✓	✓	✓	✓	✓
NRF1	✓	✓	✓	✓	✓
YY1		✓			
NFKB1 NFKB2 REL RELA	✓				✓
TEAD1 TEAD2 TEAD3 TEAD4	✓		✓		
POU1F1 POU2F1 POU2F2 POU2F3 POU3F1 POU3F2 POU3F3 POU3F4 POU5F1 POU5F1B SOX2	✓				
SOX1 SOX10 SOX11 SOX14 SOX15 SOX17 SOX18 SOX2 SOX21 SOX3 SOX4 SOX7 SOX8 SOX9 SRY				✓	
ESR1 ESR2 PPARG			✓		
CTCF	✓	✓	✓	✓	✓
REST	✓		✓	✓	✓
BACH1 BACH2 MAF MAFA MAFB MAFF MAFG MAFK MAFK NFE2 NFE2L2 NRL	✓	✓	✓	✓	
TFAP2A TFAP2B TFAP2C TFAP2E					✓
EGR1 EGR2 EGR3 EGR4	✓	✓	✓		
TP53 TP63 TP73	✓			✓	✓
CEBPA					✓
HBP1			✓		
SMAD3		✓			

TF family found by iterative *de novo* motif analysis in a specific cell line/tissue is denoted by check mark (✓).

### Breast cancer-associated genetic variants affect TF binding

**Fig 5:**
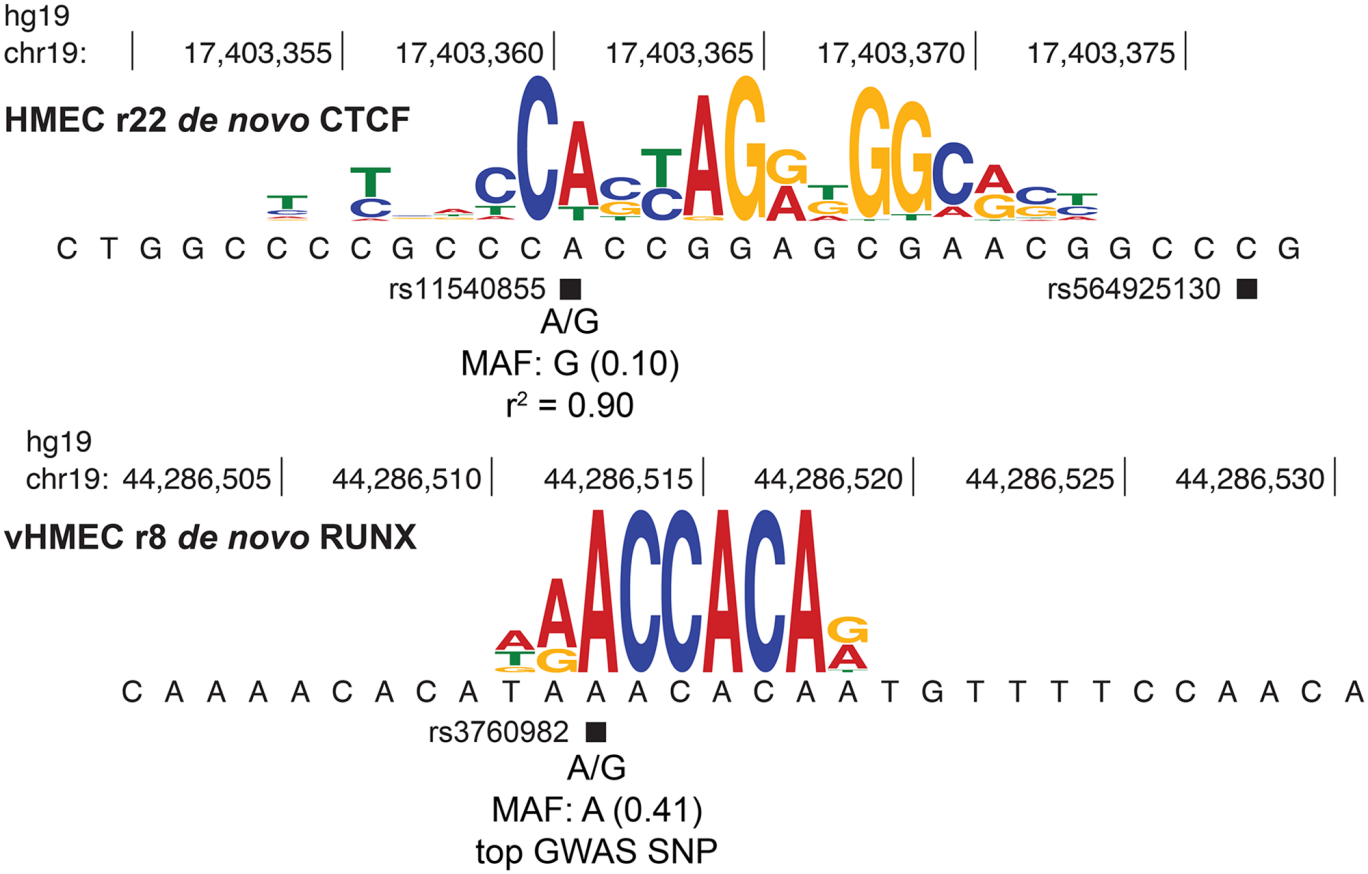
Two examples of SNPs that are in strong LD (*r*^2^ ≥ 0.8) with the most associated breast cancer GWAS SNP and are predicted to affect the binding of TFs in breast cancer-relevant tissue and cell lines. Reference SNP rs11540855 is in strong LD (*r*^2^ = 0.90) with the breast cancer GWAS SNP rs8170. The risk allele G of rs11540855 (MAF = 0.10) is predicted to compromise the binding of CTCF, which was identified in round 22 of *de novo* motif analysis from HMECs. Reference SNP rs3760982 is the most associated breast cancer GWAS SNP. Its risk allele A (MAF = 0.41) is predicted to enhance the binding of RUNX, which was identified in round 8 of *de novo* motif analysis from vHMEC.

A major goal of this study was to identify a set of plausible causal SNPs that modulate TF binding from a list of SNPs associated with breast cancer in GWAS-defined loci. We identified a total of 463 SNPs in strong LD (*r*^2^ ≥ 0.8) with the most associated breast cancer GWAS SNPs defined in 93 distinct genomic loci; these SNPs are predicted to affect the binding of TFs belonging to at least 30 TF families. Six examples of candidate causal breast cancer-associated SNPs are shown in Table 2. Transcription factors from the following TF families are predicted to have their binding affected: CTCF, GABPA, RUNX, GRHL2, USF1, ZBTB33, and ZNF143. For example, SNP rs11540855 (3′ UTR of ABHD8 on chromosome 19p13.11) is within a DNase-defined regulatory element in human mammary epithelial cells and should affect the binding of CTCF (Fig 5). rs11540855 is in strong LD with rs8170 (*r*^2^ = 0.98), which is associated with breast cancer risk [58–60]. Likewise, rs3760982 (1.1kb 5′ of KCNN4 on chromosome 19q13.31) is associated with breast cancer susceptibility [3] and we find that its A allele is predicted to enhance RUNX binding (Fig 5). More examples of candidate causal breast cancer-associated SNPs disrupting TF binding sites within breast cancer GWAS loci are shown in the supporting information (S4 Fig).

**Table 2:** Six examples of SNPs that are associated with breast cancer susceptibility and predicted to affect TF binding and gene expression regulation. Candidate SNPs are: 1) in strong LD (*r*^2^ ≥ 0.08) with the most associated breast cancer GWAS SNP; 2) within DNase/ATAC-seq defined regulatory region of MCF7, MCF10A, T47D, HMEC or vHMEC cell lines and tissue; 3) contain high information content (IC) in the TF binding PSWM (*IC* ≥ 0.5); and 4) are eQTLs in breast cancer patient solid tumor sample and GTEx breast tissue.

Candidate Causal SNP	Breast Cancer GWAS SNP	Affect Gene Expression	TF Affected Binding	eQTL P-value in Breast Cancer Tumor	eQTL P-value in Breast Tissue
rs11540855	rs8170 *r*^2^ = 0.90	ANKLE1	Affects CTCF binding	2.75E-10	9.40E-07
rs73509996	rs8170 *r*^2^ = 1.00	ANKLE1	Affects GABPA binding	4.35E-10	7.40E-07
rs3760982	rs3760982	ZNF404	Affects RUNX binding	3.16E-07	1.50E-06
rs11665924	rs3760982 *r*^2^ = 1.00	ZNF404	Affects GRHL2 binding	3.40E-07	1.50E-06
rs11669175	rs3760982 *r*^2^ = 1.00	ZNF404	Affects USF1 binding Affects ZBTB33 binding	1.13E-06	1.50E-06
rs4802200	rs3760982 *r*^2^ = 1.00	ZNF404	Affects ZNF143 binding	2.26E-06	3.00E-06

**Fig 6:**
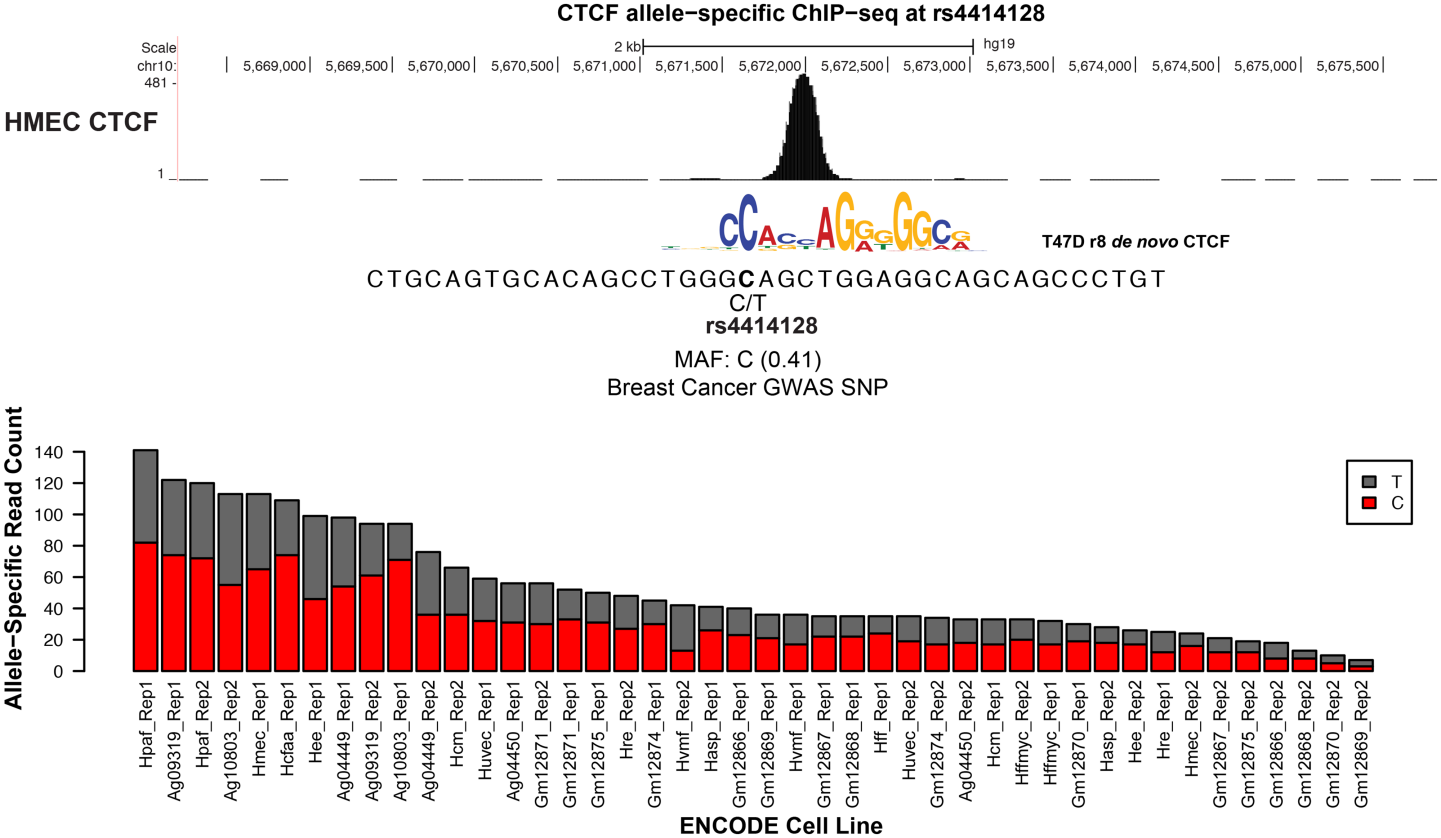
Reference SNP rs4414128 affects CTCF binding as measured by allele-specific ChIP-seq among many diploid, heterozygous cell lines. We analyzed allele-specific binding of all ENCODE cell lines with reported normal karyotype that are heterozygous at rs4414128. CTCF binding is unbalanced in favor of the C allele, which conforms more strongly to the consensus sequence.

Two SNPs (rs4414128 and rs8103622) are predicted to strongly affect CTCF binding (Fig 6 and S5 Fig); therefore, we tested our predictions of how the alleles would affect CTCF binding by analyzing ENCODE ChIP-seq data. The SNPs rs4414128 and rs8103622 showed allele-specific binding that was consistent with the direction predicted based on tolerated degeneracy from the consensus binding site (Fig 6 and S5 Fig). For rs4414128 (Fig 6), 34 of 44 cell types/replicates show the expected C preference with a range between 52% and 75%. Eight cell lines/replicates show modest allelic imbalance favoring the T allele (31–49% of the reads spanning the SNP); two experiments are balanced. Importantly, both replicates of human mammary epithelial cells (HMEC) show an allelic imbalance favoring C in 58% and 75% of the reads. Across 37 cell types (and replicates) that are heterozygous and normal karyotype, rs8103622 (S5 Fig) shows allele-specific preference of C as expected in 34 cell types/replicates with the range between 53% and 88%. The other three instances exhibit allelic balance, with allele frequencies between 48% and 52%.

### SNPs that modulate TF binding correlate with ZNF404 and ANKLE1 expression

**Fig 7:**
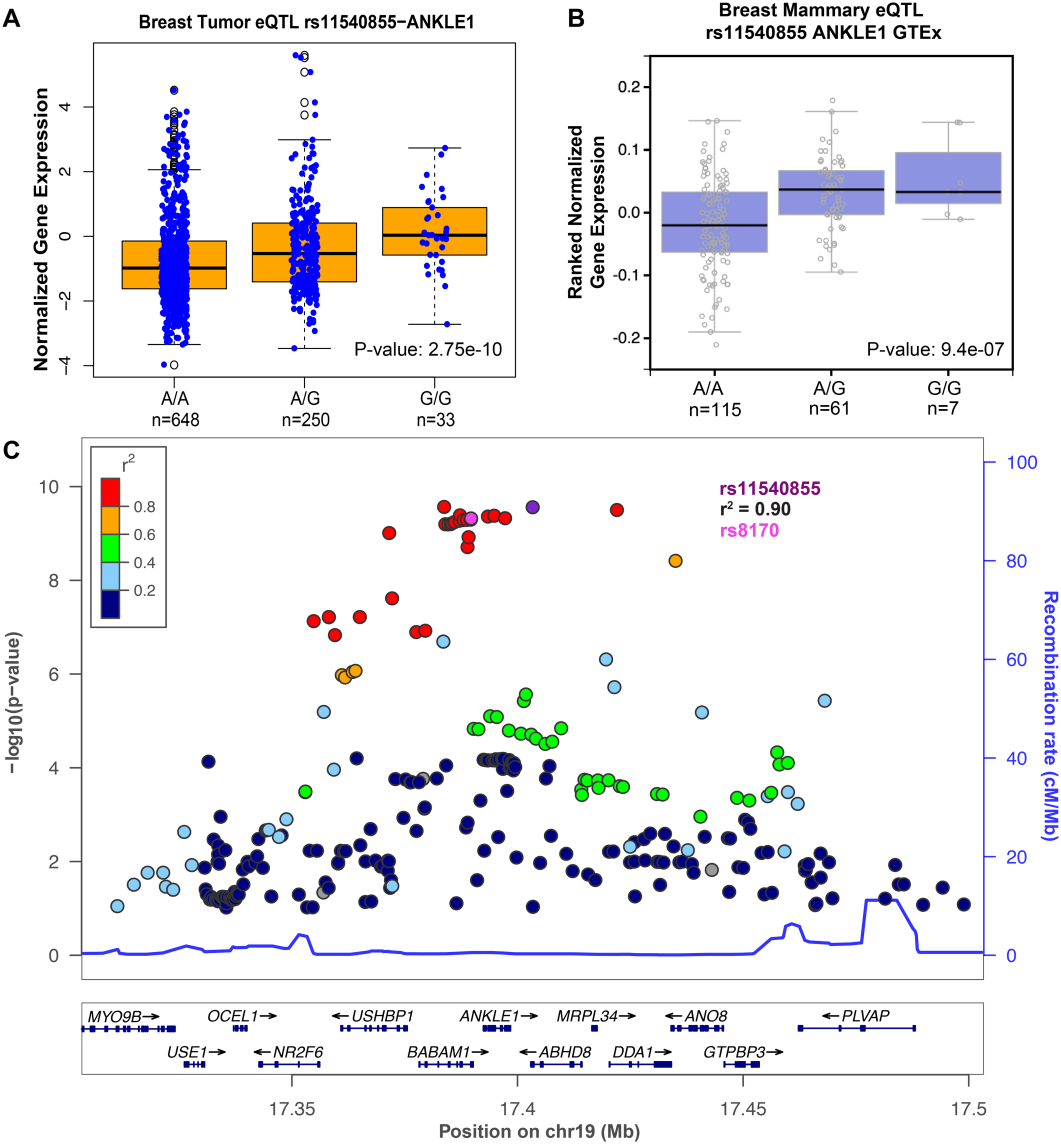
Candidate causal SNP rs11540855 is the strongest eQTL for ANKLE1. (A) rs11540855 genotypes are correlated with ANKLE1 expression. Genotype data was imputed from TCGA breast cancer patient blood sample and gene expression is from TCGA breast cancer patient solid tumor samples [57]. (B) GTEx data confirm the association of rs11540855 genotype with ANKLE1 expression using breast cancer tissue expression data. (C) SNP rs11540855 is the top eSNP in the same haloptype region. The SNP rs8170 (highlighted in pink) is the most associated GWAS hit for breast cancer susceptibility and rs8170 is in strong LD (*r*^2^ = 0.90) with rs11540855.

**Fig 8:**
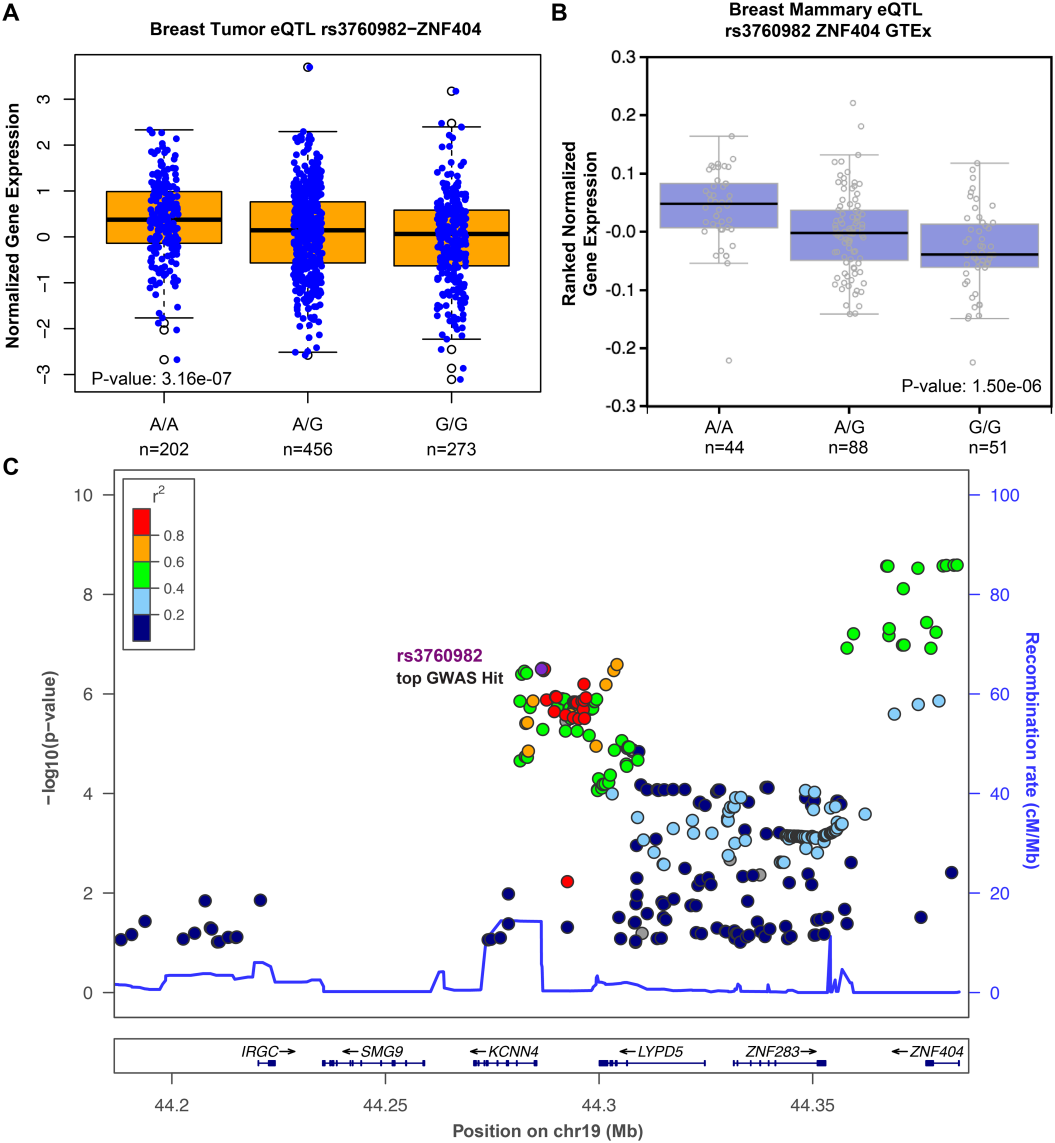
Candidate causal SNP rs3760982 is one of the top eQTLs for gene ZNF404. (A) The A/A genotype at rs3760982 is correlated with higher expression of ZNF404 in breast cancer patient solid tumor samples. (B) GTEx data confirm that the A/A genotype at rs3760982 is correlated with higher expression of ZNF404 in breast tissue. (C) rs3760982 is one of the top eQTL SNPs of ZNF404 and rs3760982 is the most associated GWAS hit for breast cancer susceptibility.

We identified rs11540855 as the most significant (P-value: 2.75E-10) eQTL SNP in the GWAS locus that is in strong LD (*r*^2^ = 0.90) with the most associated breast cancer GWAS SNP rs8170 [58] (Fig 7C). The G/G genotype at rs11540855 is predicted to compromise CTCF binding (Fig 5) and G/G individuals have, on average, higher expression levels of ANKLE1 (Fig 7A and 7B). The most significantly associated GWAS SNP in this locus (rs8170-T) increases the risk of ER-negative breast cancer with an odds ratio of 1.10 [60] and the T allele is in LD with the G allele of rs11540855. Therefore, higher expression of ANKLE1 (Fig 7A and 7B) is associated with increased breast cancer risk. We also identified rs3760982 as one of the most associated eQTL SNPs (P-value: 3.16E-07) and rs3760982 is correlated with ZNF404 expression (Fig 8C). The A/A genotype at rs3760982 is predicted to increase RUNX binding (Fig 5) and is correlated with higher expression of ZNF404 in breast cancer tumor samples (Fig 8A) and breast tissue (Fig 8B). The rs3760982-A allele is associated with an increased risk (odds ratio of 1.06) of breast cancer [3], thus higher expression of ZNF404 correlates with increased breast cancer risk. Therefore, we prioritized SNPs within GWAS loci that are predicted to affect transcription factor binding and module expression of ANKLE1 and ZNF404 to confer breast cancer risk.

## Discussion

GWAS have discovered more than 90 genetic loci and common genetic variants associated with breast cancer susceptibility [1–5], and the majority of SNPs in these loci are enriched in non-coding regions. Non-coding genetic variants can contribute to complex traits and diseases through many molecular mechanisms [12–14, 61, 62] including having an effect on TF binding affinities, which can result in differential gene expression. Herein, we describe an integrative genomics methodology to identify a near-comprehensive set of TFs that are actively maintaining open chromatin in a cell type. We identify which GWAS-relevant SNPs are predicted to modulate TF binding intensity and use ChIP-seq data to confirm our predictions. Lastly, we use eQTL data to identify the likely target genes of SNPs that affect TF binding affinity. Taken together, this approach can identify likely causal SNPs associated with breast cancer risk.

We found that rs3760982 variants are predicted to modulate RUNX binding (Fig 5) and we confirm previous work showing that rs3760982 is an eQTL for ZNF404 (Fig 8) [3, 63]. The A allele of rs3760982 conforms more stringently to the RUNX consensus sequence and this allele is predicted to enhance RUNX binding; allele-specific ChIP-seq in breast tissue, would test whether RUNX family TFs prefer binding the A allele *in vivo*. The RUNX family of TFs are canonical transcriptional activators [64–67], so we hypothesize that increased RUNX binding is a mechanism by which the ZNF404 is regulated. Testing this hypothesis would necessitate specific gene editing of rs3760982 and subsequent measurements of ZNF404 expression. While CRISPR-mediated [68] deletions of genetic elements is routine, precise changes of specific alleles remains a challenge. Deletion of the rs3760982 variant by CRISPR, followed by measuring ZNF404 expression would confirm or refute the role of rs3760982 variants in ZNF404 expression. One could also test allele-specific expression of alleles within transcription units that are phased with rs3760982 variants. We propose using precision global run-on sequencing [69] to measure nascent RNA expression to capture informative intronic SNPs. While genomic approaches are a means to develop novel hypotheses, the advent of genetic editing approaches permits hypothesis testing to define mechanisms by which genes and genetic variants contribute to disease risk.

We found that rs11540855 is an eQTL for ANKLE1 (Fig 7) and rs11540855 variants are predicted to affect CTCF binding (Fig 5). The rs11540855 SNP is in high LD (*r*^2^ = 0.90) with the breast cancer-associated GWAS SNP rs8170, which was first found as a modifier of breast cancer risk in BRCA1 mutation carriers [58]. Subsequently, this SNP was found to be associated with breast cancer susceptibility in ER-negative breast cancer [58–60]. ANKLE1 is an evolutionarily conserved non-membrane-bound LEM protein that harbors endonuclease activity, but its cellular functions remain uncharacterized [70, 71]. Future work will need to determine the allele-specificity of CTCF binding at rs11540855 and test the role that this site has upon ANKLE1 expression. These approaches will be able to define the relationship between TF binding and gene expression, but it is challenging to develop a physiologically relevant model of breast cancer risk that incorporates human genetic variation. GWAS-identified SNPs are common and typically confer relatively small differences in risk. Further, the cumulative affects of differential gene expression over the lifetime of an individual cannot be easily recapitulated in a controlled environment.

Our research identified a previously uncharacterized DNA sequence motif that is enriched in open chromatin, evolutionarily conserved, and is associated with directional hypersensitivity profiles (Figs 2 and 3). We hypothesize that this orphan motif is the recognition site for a previously uncharacterized transcription factor. Future work, such as DNA affinity chromatography [72], will be needed to identify this candidate TF.

Genomic approaches are ideally suited to address fundamental biological questions in a relatively unbiased manner. Integrative genomic approaches and analyses can clarify the null-hypothesis and permit the development of novel hypotheses that were previously inconceivable. These approaches serve as a first-step in understanding the biology of breast cancer risk and targeted experimental follow-up is necessary to define the mechanistic roles of genes and genetic variants in breast cancer susceptibility and disease progression.

## Materials and methods

### ATAC-seq library preparation

We cultured MCF10A cells in Dulbecco’s modified Eagle’s medium (Invitrogen) with 5% horse serum (Invitrogen), 1% penicillin/streptomycin (Invitrogen), 20 ng/ml EGF (Peprotech), 0.5 *μ*g/ml hydrocortisone (Sigma), 100 ng/ml cholera toxin (Sigma) and 10 *μ*g/ml insulin (Sigma) in a humidified incubator at 37°C with 5% CO_2_. The ATAC-seq library was prepared as previously described [73] with several modifications: 1) IGEPAL CA-630 was omitted from the lysis buffer; 2) we performed two additional wash steps with lysis buffer; and 3) we performed PCR-clean up using AMPure XP beads to select DNA <600 bp. The MCF10A ATAC-seq data were deposited in the Gene Expression Omnibus (GEO) database, with accession number GSE89013. We mapped reads to the hg38 human reference genome using Bowtie2 [74] and merged replicate aligned files. We used the merged data for all subsequent analysis; refer to S1 File for ATAC-seq data analysis details.

### Iterative *de novo* motif analysis from regulatory regions

We performed iterative rounds of *de novo* motif analysis using a 120-base pair window centered on the summit of hypersensitivity as defined by ATAC-seq or DNase-seq (S1 File). In each cell type we found hundreds of over-represented position specific weight matrices (PSWMs). We identified all instances of each PSWM within breast-specific regulatory elements, while accounting for the possibility that the reference genome contains variants that will conform more or less strictly to the PSWM. This approach allowed us to identify potential binding sites that contain SNPs, even if the reference allele does not match the queried PSWM.

### Clustering of *de novo* found motif PSWMs

Although we identified hundreds of distinct PSWMs, many PSWMs are similar to one another and are likely to represent redundant specificity of a single TF or TF family. To consolidate similar PSWMs into known TF families, we systematically classified several public PSWM repositories [43–47] into families with distinct features. PSWMs were first divided into clusters based on connectivity; connectivity between motif nodes was measured by negative log_10_ E-value as calculated by TOMTOM [75]. An edge was inferred between two motif nodes if their similarity exceeded a negative log_10_ E-value of 10. We defined a motif cluster as a connected set of nodes; connectivity is defined by the existence of a path between every pair of nodes. A fast greedy modularity algorithm [76] further divided each motif cluster into families.

### Identification of SNPs that affect binding of TFs

We downloaded a curated list of breast cancer associated SNPs from the GWAS catalog [77]. The SNP that exhibits the most statistically significant association with breast cancer in any locus may not be causal due to linkage disequilibrium (LD) and the sampling variation that interrogated the specific SNP. To better define the list of likely causal variants for each locus, we identified all SNPs satisfying the following three criteria: 1) SNPs that are in strong LD (*r*^2^ ≥ 0.8) with the most significant reported GWAS SNP; 2) SNPs that are located within putative TF binding sites identified by hypersensitivity assays; and 3) SNPs that are within critically important positions that affect TF binding affinity (Information Content ≥ 0.5).

### TF allele-specific binding preference analysis

We analyzed ENCODE CTCF ChIP-seq data for allele-specific preference of SNPs that are predicted to modulate CTCF binding affinity. All CTCF ChIP-seq data are provided within S1 File. We analyzed the highest intensity CTCF sites to ensure that sequencing reads would span the query SNP. We exclusively queried normal karyotype cell lines that had SNPs that were heterozygous within each locus to reduce the chances that copy number variations (i.e., aneuploidy) of alleles would skew our analyses.

### eQTL analysis

To identify putative causal genes whose expression may be affected by polymorphisms, we performed eQTL analysis using TCGA breast cancer data [57] with fastQTL [78]. We imputed the genotypes from TCGA SNP6 arrays that were hybridized with DNA extracted from the blood of patients with breast cancer. We retrieved genotype data from dbGaP (phs000178.v9.p8) and imputed genotypes using the Michigan Imputation Server [79] with the following parameters: 1000G Phase 1 v3 Shapeit2 Reference Panel, ShapeIT Phasing, Mixed Population, and Quality Control/Imputation Mode. Following imputation, we removed SNPs with the following features: Hardy-Weinberg Equilibrium *p* < 1 × 10^−6^ and minor allele frequency (MAF) < 5%. We used UCSC-curated TCGA RNA-seq data [57] from breast cancer patient solid tumor samples as the gene expression data to identify eQTLs. TCGA clinical data were incorporated as the covariates for eQTL analysis such as sample RNA concentration, RIN value, sex, and ethnicity. We performed Principal Component Analysis (PCA) on the quantitative variables from clinical data and used the first three principal components as covariates. We retained other qualitative variables as categorical covariates.

## Supporting information

S1 File A step-by-step guide to reproducing this publication’s analyses and results. In this vignette, we provide data sources for DNase-seq data, ChIP-seq data, TCGA RNA-seq data, TCGA genotype data, TCGA phenotype data, breast cancer GWAS catalog information, and PSWM databases used in our study. We provide detailed documentation of the computational analyses we performed.
(PDF)

S2 File A bundle of all the individual executable scripts we used in this publication. This zipped file contains the full set of R, Python, and Shell scripts used in this publication.
(ZIP)

S1 Fig All the motifs found by iterative *de novo* motif analysis in the five data sets mapped to 37 distinct motif families. (A) We clustered several publicly available PSWMs into families based on their similarities. In this illustration each node is a PSWM and the size of each node is proportional its number of edges. Contiguously connected nodes are colored uniquely and each family of PSWM is outlined by a distinct background color. We inferred edges using TOMTOM [75] to match PSWMs. The width of edges denote the similarity between two PSWMs and this width is proportional to the −log_10_ E-value of the match. (B) TFs are organized into families that recognize similar sequences and a representative motif for each regulatory sequence family is presented as a seqLogo. For example, three known TFs (ESR1, ESR2, and PPARG-*γ*) recognize the estrogen response element (ERE).
(TIF)

S2 Fig TF members in the same family have different levels of gene expression in the solid tumor sample of breast cancer patients. Each TF is numbered in accordance with its row number in Table 1. Normalized RNA-seq gene expression data are from TCGA breast cancer patient solid tumor samples [57].
(TIF)

S3 Fig TF expression correlates with patient outcome in a subtype-specific manner. High expression of IRF7, BATF, and TP73 is correlated with better breast cancer patient outcome in Luminal A, HER2+, and Basal-like subtype, respectively. TF expression groups are classified as either high or low expression and with the corresponding patient number is noted in the parentheses. We use FDR to correct the P-values for multiple testing. Kaplan-Meier analysis is performed using TCGA breast cancer patient solid tumor sample RNA-seq expression and overall breast cancer patient survival data [57].
(TIF)

S4 Fig Candidate SNPs are predicted to modulate the binding of breast cancer-relevant TFs. Each SNP is in strong LD (*r*^2^ ≥ 0.8) with the most associated breast cancer GWAS SNP and affects TF binding affinity. We report the minor allele frequency (MAF) and LD association (*r*^2^) of each SNP with the most associated breast cancer GWAS SNP.
(TIF)

S5 Fig Genotypic variants of rs8103622 affect CTCF binding in many cell types. ENCODE ChIP-seq data (top) indicates that CTCF is strongly bound in HMEC at rs8103622. The risk allele C of rs8103622 is predicted to increase CTCF binding, which was identified in round 33 of our *de novo* motif analysis from MCF7 (middle). By analyzing ENCODE ChIP-seq count data, we show that there is an allelic imbalance favoring C (denoted in red) versus T (denoted in black) in 34 out of 37 cell types/replicates that are diploid and heterozygous for the C/T allele at rs8103622.
(TIF)
