# Supplementary material for: Identification of breast cancer associated variants that modulate transcription factor binding
Source: PLoS Genet. 2017 Sep 28;13(9):e1006761. doi: 10.1371/journal.pgen.1006761 (PMC5619690; doi:10.1371/journal.pgen.1006761)
Supplement: S1 File — In this vignette, we provide data sources for DNase-seq data, ChIP-seq data, TCGA RNA-seq data, TCGA genotype data, TCGA phenotype data, breast cancer GWAS catalog information, and PSWM databases used in our study. We provide detailed documentation of the computational analyses we performed. (PDF) [file pgen.1006761.s001.pdf]

Identification of Breast Cancer Associated Variants That Modulate Transcription Factor Binding – Vignette

Yunxian (Fureya) Liu

2nd March 2017

Contents

1 *De novo* motif analysis of breast-relevant DNase-seq data **1**
1.1 Retrieving raw DNase-seq data . . . 2
1.1.1 broadPeak files . . . 2
1.1.2 bigWig files . . . 2
1.2 Pre-processing the data files . . . 2
1.2.1 broadPeak files . . . 2
1.2.2 Combine files for each condition together . . . 3
1.2.3 bigWig files . . . 4
1.3 Find the appropriate genome file . . . 4
1.4 *De novo* motif analysis with MEME Suite Software . . . 5
1.4.1 Find hotspots with highest intensity . . . 5
1.4.2 *De novo* motif analysis with one-stop iteration and MEME-ChIP . . . 5

2 *De novo* motif analysis of ATAC-seq data **6**
2.1 Producing broadPeak and bigWig files from Fastq files . . . 6
2.2 *De novo* motif analysis with MEME . . . 8

3 DNase composite foot printing analysis and composition conservation **8**
3.1 Retrieving raw FASTQ data sets . . . 9
3.1.1 Obtaining naked DNase-seq single nucleotide resolution data of human fibroblast (IMR-90) cells . . . 9
3.1.2 Obtaining ENCODE FASTQ data sets for breast cancer cells . . . 9
3.2 Aligning sequencing reads to reference hg19 genome sequences . . . 10
3.3 Producing bedGraph and bigWig files from SAM output files . . . 10
3.4 Retrieving conservation data sets . . . 11
3.5 Making composite plots for DNase-seq and conservation data . . . 11

4 Clustering *de novo* found regulatory sequences to unique transcription factor family **12**
4.1 Construction of motif families based on TOMTOM and HOMER motif databases . . 12
4.1.1 Construction of motif family with members connecting to each other or through the other motifs . . . 14
4.1.2 Construction of motif families by community detection algorithm . . . 14
4.2 Identification of unique transcription factor families for each cell line . . . 17
4.3 Identification of orphan motifs . . . 23
4.3.1 Normalize bigWig files - *seqOutBias* . . . 23
4.3.2 Make conservation plot and DNase footprint plot for the orphan motifs . . . 25
4.3.3 Rerun TOMTOM with a less stringent threshold . . . 28
4.3.4 Post-hoc processing the information and identify candidate orphan motifs . . 29

5 Defining single nucleotide variates that may affect transcription factor binding in breast cancer **29**
5.1 Find all regions that are in LD with the top GWAS SNPs 29
5.2 Identification of all potential transcription factor binding sites underlying DNase/ATAC-defined regulatory region and overlapping with the GWAS LD regions 29
5.3 Determination of the polymorphism of TFBS 33
5.4 Linking candidate causal variants to genes – eQTL analysis 37
5.4.1 Retrieving TCGA gene expression data 37
5.4.2 TCGA patients genotype data 37
5.4.3 Retrieving TCGA clinical information 38
5.4.4 eQTL analysis using fastQTL 39
5.5 Allele-specific TF binding 40
5.6 TFBS polymorphism and the expressions of breast cancer TCGA tissue phenotype . 47

6 Correlation analysis of patient survival data with TF expression from the Cancer Genome Atlas data **50**

Appendix A Bash shell script: add a function to the executable path **55**

Appendix B R script: calculate the probes with highest intensity **55**

Appendix C Bash shell script: *de novo* motif finding with one-stop iteration and MEME-ChIP **56**

Appendix D Python script: reformat MAST text file to BED file **62**

Appendix E Python script: MEME matrix wrapper **63**

Appendix F Python script: production of individual motif meme-format files form the motif database **64**

Appendix G Python script: converting Sam file to Bed file by ARQ **65**

Appendix H Python script: fixing the name for Bed file **67**

Appendix I Token file: parameters (tokens) used by Hotspot for calling hotspot **68**

Appendix J Hotspot executable file: running Hotspot program in the terminal **70**

Appendix K Python script: converting Sam file to Bed file **71**

Appendix L R script: production of composite plots for DNase-seq and conservation data with MAST output files **72**

Appendix M Python script: identification of motif families **77**

Appendix N R script: production of composite plots for DNase-seq and conservation data with orphan MAST output files **79**

Appendix O Python script: scramble motif from a minimal meme file **85**

Appendix P R script: production of composite plots for DNase-seq and conservation data with scrambled orphan MAST output files **86**

Appendix Q Python script: production of degenerate PSWM from a minimal meme file **93**

Appendix R `R` **script: obtain the information content in single nucleotide resolution with a designated threshold by André Martins** **95**

Appendix S Bash shell script: obtain the TCGA patient genotype data from birdseed files and imputation with Michigan Imputation Server **100**

Appendix T `R` **script: eQTL analysis of our candidate SNPs with genes within 1 Mb** **103**

Appendix U Bash shell script: zip and index the genotype file for eQTL analysis **113**

Appendix V Bash shell script: eQTL analysis by fastQTL **113**

References **116**

1 *De novo* motif analysis of breast-relevant DNase-seq data

DNase-seq identifies regulatory regions genome-wide based on their relative sensitivity to cleavage by the DNase I enzyme (Boyle *et al.*, 2008). Each DNase-seq data used in our study was obtained from UCSC ENCODE consortium (`https://genome.ucsc.edu/ENCODE/`) and NIH Roadmap Epigenomic consortium (`http://www.roadmapepigenomics.org/`).

- **ENCODE data**
  path: `http://hgdownload.cse.ucsc.edu/goldenPath/hg19/encodeDCC/wgEncodeUwDnase/`
  files:
  wgEncodeUwDnaseMcf7Estctrl0hHotspotsRep1.broadPeak.gz
  wgEncodeUwDnaseMcf7Estctrl0hHotspotsRep2.broadPeak.gz
  wgEncodeUwDnaseMcf7Est100nm1hHotspotsRep1.broadPeak.gz
  wgEncodeUwDnaseMcf7Est100nm1hHotspotsRep2.broadPeak.gz
  wgEncodeUwDnaseMcf7Estctrl0hRawRep1.bigWig
  wgEncodeUwDnaseMcf7Estctrl0hRawRep2.bigWig
  wgEncodeUwDnaseMcf7Est100nm1hRawRep1.bigWig
  wgEncodeUwDnaseMcf7Est100nm1hRawRep2.bigWig
  wgEncodeUwDnaseMcf7Est100nm1hRawDataRep1.fastq.tgz
  wgEncodeUwDnaseMcf7Est100nm1hRawDataRep2.fastq.tgz
  wgEncodeUwDnaseMcf7Estctrl0hRawDataRep1.fastq.tgz
  wgEncodeUwDnaseMcf7Estctrl0hRawDataRep2.fastq.tgz
  wgEncodeUwDnaseT47dHotspotsRep1.broadPeak.gz
  wgEncodeUwDnaseT47dHotspotsRep2.broadPeak.gz
  wgEncodeUwDnaseT47dRawRep1.bigWig
  wgEncodeUwDnaseT47dRawRep2.bigWig
  wgEncodeUwDnaseT47dRawDataRep1.fastq.tgz
  wgEncodeUwDnaseT47dRawDataRep2.fastq.tgz
  wgEncodeUwDnaseHmecHotspotsRep2.broadPeak.gz
  wgEncodeUwDnaseHmecRawRep2.bigWig
  wgEncodeUwDnaseHmecRawDataRep2.fastq.gz

- **Roadmap Epigenomic data**
  Contact Richard Sandstrom (sull@uw.edu) for access through the following site: `http://www.uwencode.org/`
  files: p1.vHMEC-DS18438.75.20.filtered-density.36.hg19.bw
  p1.vHMEC-DS18406.75.20.filtered-density.36.hg19.bw
  vHMEC-DS18406.peaks.fdr0.01.hg19.bed.gz
  vHMEC-DS18438.peaks.fdr0.01.hg19.bed.gz

We conducted *de novo* motif finding analysis on DNase-seq data collected from breast cancer-relevant cells or tissue including MCF7 cells, T47D cells, cultured human mammary epithelial cells (HMEC), and primary breast variant HMEC (vHMEC). We will use MCF7 Dnase-seq data as an example in the following sections. The treated MCF7 cells were incubated with 100 nM estradiol (in EtOH) for 1 hour; the control cells were incubated for 1 hour with EtOH. Two replicates were conducted for each condition. Many of the files were preprocessed by ENCODE using the hg19 assembly, so we continued to use hg19 for this entire work flow.

1.1 Retrieving raw DNase-seq data

Access the ENCODE production data from Human Genome Build 37 (hg19): http://genome.ucsc.edu/ENCODE/downloads.html. We chose the DNaseI Hypersensitivity by Digital DNaseI from ENCODE/University of Washington (UW DNaseI HS) in the chromatin accessibility category.

1.1.1 broadPeak files

First, download the corresponding broadPeak files. ENCODE broadPeak format is used to provide called regions of signal enrichment based on pooled, normalized data. You can find more information about this format from https://genome.ucsc.edu/FAQ/FAQformat.html#format13. ALternatively use *wget* as opposed to *curl*.

```
curl -OL http://hgdownload.cse.ucsc.edu/goldenPath/hg19/encodeDCC/\
   wgEncodeUwDnase/wgEncodeUwDnaseMcf7Estctrl0hHotspotsRep1.broadPeak.gz
curl -OL http://hgdownload.cse.ucsc.edu/goldenPath/hg19/encodeDCC/\
   wgEncodeUwDnase/wgEncodeUwDnaseMcf7Estctrl0hHotspotsRep2.broadPeak.gz
curl -OL http://hgdownload.cse.ucsc.edu/goldenPath/hg19/encodeDCC/\
   wgEncodeUwDnase/wgEncodeUwDnaseMcf7Est100nm1hHotspotsRep1.broadPeak.gz
curl -OL http://hgdownload.cse.ucsc.edu/goldenPath/hg19/encodeDCC/\
   wgEncodeUwDnase/wgEncodeUwDnaseMcf7Est100nm1hHotspotsRep2.broadPeak.gz
```

Unzip all data files.

```
gunzip wgEncodeUwDnaseMcf7Est100nm1hHotspotsRep1.broadPeak.gz
gunzip wgEncodeUwDnaseMcf7Est100nm1hHotspotsRep2.broadPeak.gz
gunzip wgEncodeUwDnaseMcf7Estctrl0hHotspotsRep1.broadPeak.gz
gunzip wgEncodeUwDnaseMcf7Estctrl0hHotspotsRep2.broadPeak.gz
```

1.1.2 bigWig files

Second, download the corresponding bigWig files. The bigWig format is for display of dense, continuous data that will be displayed in the Genome Browse as a graph. You can find more information about this format from https://genome.ucsc.edu/goldenPath/help/bigWig.html.

```
curl -OL http://hgdownload.cse.ucsc.edu/goldenPath/hg19/encodeDCC/\
   wgEncodeUwDnase/wgEncodeUwDnaseMcf7Estctrl0hRawRep1.bigWig
curl -OL http://hgdownload.cse.ucsc.edu/goldenPath/hg19/encodeDCC/\
   wgEncodeUwDnase/wgEncodeUwDnaseMcf7Estctrl0hRawRep2.bigWig
curl -OL  http://hgdownload.cse.ucsc.edu/goldenPath/hg19/encodeDCC/\
   wgEncodeUwDnase/wgEncodeUwDnaseMcf7Est100nm1hRawRep1.bigWig
curl -OL  http://hgdownload.cse.ucsc.edu/goldenPath/hg19/encodeDCC/\
   wgEncodeUwDnase/wgEncodeUwDnaseMcf7Est100nm1hRawRep2.bigWig
```

1.2 Pre-processing the data files

1.2.1 broadPeak files

- **Combine the replicates for each condition**
  Use *cat* function to combine the contents in two files into one.
  - Treatment groups

```
cat wgEncodeUwDnaseMcf7Est100nm1hHotspotsRep1.broadPeak \
   wgEncodeUwDnaseMcf7Est100nm1hHotspotsRep2.broadPeak > \
   E2_100nM_1hr_ Hotspots.broadPeak
```

– Control groups

```
cat wgEncodeUwDnaseMcf7Estctrl0hHotspotsRep1.broadPeak \
    wgEncodeUwDnaseMcf7Estctrl0hHotspotsRep2.broadPeak > \
    E2_0hr_Hotspots.broadPeak
```

- **Use bedtools (v2.25.0) to sort and then merge within each condition**
  Bedtools is a powerful toolset for operating on genomic intervals (http://bedtools.readthedocs.org/en/latest/). For example, bedtools allows one to *intersect, count, complement, and shuffle* genomic coordinates from multiple files using many file formats as input.

  By default, *sortBed* sorts a BED file by chromosome and then by start position in ascending order (http://bedtools.readthedocs.org/en/latest/content/tools/sort.html).

  mergeBed combines overlapping features in an interval file into a single feature which spans all of the combined features (http://bedtools.readthedocs.org/en/latest/content/tools/merge.html). *mergeBed* requires that you presort your data by chromosome and then by start position.

  – Treatment group

```
sortBed -i E2_100nM_1hr_Hotspots.broadPeak > E2_100nM_1hr_Hotspots.\
    sort.broadPeak
mergeBed -i E2_100nM_1hr_Hotspots.sort.broadPeak > \
    E2_100nM_1hr_Hotspots.merge.broadPeak
```

  – Control group

```
sortBed -i E2_0hr_Hotspots.broadPeak > E2_0hr_Hotspots.sort.\
    broadPeak
mergeBed -i E2_0hr_Hotspots.sort.broadPeak > E2_0hr_Hotspots.merge.\
    broadPeak
```

1.2.2 Combine files for each condition together

Again, use *cat* function to combine the contents in two files into one.

```
cat E2_0hr_Hotspots.merge.broadPeak E2_100nM_1hr_Hotspots.merge.broadPeak >\
     Hotspots_Either_E2_condition.cat.broadPeak
```

sortBed sorts a BED file by chromosome and then by start position in ascending order.

```
sortBed -i Hotspots_Either_E2_condition.cat.broadPeak > \
   Hotspots_Either_E2_condition.sort.broadPeak
```

mergeBed combines overlapping features in an interval file into a single feature which spans all of the combined features.

```
mergeBed -i Hotspots_Either_E2_condition.sort.broadPeak > \
   Hotspots_Either_E2_condition.merge.broadPeak
```

Or you can wrap cat, sort and merge into a pipe (use piping command |).

```
cat E2_0hr_Hotspots.merge.broadPeak E2_100nM_1hr_Hotspots.merge.broadPeak |\
    sort -k1,1 -k2,2n | mergeBed > Hotspots_Either_E2_condition.merge.pip.\
   broadPeak
```

1.2.3 bigWig files

- **Merge all bigWig files**
  You need to use *bigWigMerge* function from UCSC tools to merge bigWig files, a bedGraph file will be generated. *bigWigMerge* function can be downloaded from: `http://hgdownload.soe.ucsc.edu/admin/exe/macOSX.x86_64/bigWigMerge`. Command *chmod +x* made the file executable.

  ```
  chmod +x ~/bigWigMerge
  ```

  Make sure to add this function to your executable path (Appendix A).

  ```
  bigWigMerge wgEncodeUwDnaseMcf7Est100nm1hRawRep1.bigWig \
     wgEncodeUwDnaseMcf7Est100nm1hRawRep2.bigWig \
     wgEncodeUwDnaseMcf7Estctrl0hRawRep1.bigWig \
     wgEncodeUwDnaseMcf7Estctrl0hRawRep1.bigWig bigWig.merge.bedGraph
  ```

- **Modifying files for uploading into UCSC genome browser**
  sortBed sorts a BED file by chromosome and then by start position in ascending order.

  ```
  sortBed -i bigWig.merge.bedGraph > bigWig.merge.sort.bedGraph
  ```

  Add a header and then zip the bedGraph file.

  ```
  touch temp.txt
  name=Either_E2_condition_MCF7
  echo "track type=bedGraph name=$name" >> temp.txt
  cat temp.txt bigWig.merge.sort.bedGraph > $name.bedGraph
  rm temp.txt
  gzip $name.bedGraph
  ```

- **Prepare bigWig file for finding hotspots with highest intensity**

  ```
  gunzip Either_E2_condition_MCF7.bedGraph.gz
  ```

  Convert the file into a bigWig file using function *bedGraphToBigWig*, which can be downloaded at `http://hgdownload.soe.ucsc.edu/admin/exe/macOSX.x86_64/bedGraphToBigWig`. Make sure to add this function to the PATH variable (Appendix A).

  ```
  curl -OL http://genome.ucsc.edu/goldenpath/help/hg19.chrom.sizes
  bedGraphToBigWig $name.bedGraph  hg19.chrom.sizes $name.bigWig
  ```

1.3 Find the appropriate genome file

We want the hg19 file.

```
curl -OL http://hgdownload.soe.ucsc.edu/goldenPath/hg19/bigZips/hg19.2bit
```

We need to transform the data from .2bit format to .fa format.

```
twoBitToFa hg19.2bit hg19.fa
```

1.4 *De novo* motif analysis with MEME Suite Software

1.4.1 Find hotspots with highest intensity

Use the merged broadPeak file (made in sec 1.2.2) and bigwig file (made in sec 1.2.3) to find hotspots with highest intensity. Install the bigWig library: `https://github.com/andrelmartins/bigWig`. Use `R` to find the highest intensity coordinate of each DNase peak. Definition of function *calc.highest.probe* is provided in Appendix B. The output file is a BED file: "inten_highest_hotspot.bed". Please find more details about BED format at `https://genome.ucsc.edu/FAQ/FAQformat.html#format1`.

```
library(bigWig)
#set directory to the one containing following files
hotspots = 'Hotspots_Either_E2_condition.merge.broadPeak'
highest.hotspot = calc.highest.probe(read.table(hotspots), 'Either_E2_\
    condition\_MCF7.bigWig', window =60)
x = bed.region.probeQuery.bigWig(load.bigWig('Either_E2_condition_MCF7.\
    bigWig'), highest.hotspot)
inten.highest.hotspot = cbind(highest.hotspot,x)
colnames(inten.highest.hotspot) = c('chr','start','end','intensity')
write.table(inten.highest.hotspot, file = 'inten_highest_hotspot.bed', \
    quote=F, row.names=F, col.names=F, sep= '\t')
```

1.4.2 *De novo* motif analysis with one-stop iteration and MEME-ChIP

De novo motif analysis with one-stop iteration

We conducted iterative rounds of *de novo* motif analysis of the sequences surrounding the enzymatic accessibility peak summits with a 120-base pair window (produced in section 1.4) and identified a set of overrepresented motifs within the regulatory elements, which stopped when the input fasta sequence reaches the lowest 20K peaks using several tools (including meme, mast and tomtom) provided by The MEME Suite (version 4.10.2): `http://meme-suite.org/`. We refer this process as **one-stop iterative *de novo* motif analysis**.
MEME stands for "Multiple Em for Motif Elicitation". MEME discovers novel, ungapped motifs (recurring, fixed-length patterns) in nucleotide or protein sequences (`http://meme-suite.org/doc/meme.html?man_type=web`). MEME splits variable-length patterns into two or more separate motifs. MEME takes as input a group of DNA or protein sequences and outputs as many motifs as requested. MEME uses statistical modeling techniques to automatically choose the best width, number of occurrences, and description for each motif (Bailey *et al.*, 1994). Note that meme is computational demanding.
TOMTOM compares one or more nucleotide motifs against a database of known motifs (e.g., JASPAR) (`http://meme-suite.org/doc/tomtom.html`). Motif databases could be downloaded from the *MEME Suite website* (`http://meme-suite.org/doc/download.html?man_type=web`). TOMTOM ranks the motifs in the database and produce an alignment for each significant match (Gupta *et al.*, 2007).
The name MAST stands for "Motif Alignment and Search Tool" (`http://meme-suite.org/doc/mast.html?man_type=web`). The program searches sequences for matches to a set of nucleotide or protein motifs, and sorts the sequences by the best combined match to all motifs (Bailey and Gribskov, 1998). Motifs must be in MEME Motif Format (`http://meme-suite.org/doc/meme-format.html`).

De novo motif analysis with MEME-ChIP

Following the one-stop iteration, we masked all the peaks found by the one-stop iteration and got a new fasta file containing all the coordinates non-overlapping with all the motifs found by one-stop iteration. MEME-ChIP was further used to conduct exhaustive *de novo* motif finding. MEME-ChIP executes two different motif discovery algorithms (multiple EM for motif elicitation (MEME) and discriminative regular expression motif elicitation (DREME)) to discover novel sequence motifs (Ma *et al.*, 2014). We used MEME in this step to discover 50 more motifs. DREME discovers short, ungapped motifs (recurring, fixed-length patterns) that are relatively enriched in the input sequences compared with shuffled sequences (Bailey, 2011). We used DREME here to uncover all remaining short-length motifs (up to 8 positions). We provide the bash script in Appendix C. Several embedded python scripts are provided in Appendix D (MAST_bed_conversion.py), Appendix E (MEME_matrix_wrapper.py), Appendix F (MEME_individual_from_db.py).

2 *De novo* motif analysis of ATAC-seq data

MCF10A ATAC-seq data was generated in the Guertin Laboratory and deposited to Gene Expression Omnibus (GEO) with accession number GSE89013 at `https://www.ncbi.nlm.nih.gov/geo/query/acc.cgi?acc=GSE89013`.

2.1 Producing broadPeak and bigWig files from Fastq files

- Merge and rename relevant fastq files

```
cat 05202016-Guertin_ATACSeq-30582565/1_*/*R1*.gz > MCF10a_PE1_rep1.\
    fastq.gz
cat 05202016-Guertin_ATACSeq-30582565/1_*/*R2*.gz > MCF10a_PE2_rep1.\
    fastq.gz
cat 05202016-Guertin_ATACSeq-30582565/2_*/*R1*.gz > MCF10a_PE1_rep2.\
    fastq.gz
cat 05202016-Guertin_ATACSeq-30582565/2_*/*R2*.gz > MCF10a_PE2_rep2.\
    fastq.gz
cat 05202016-Guertin_ATACSeq-30582565/3_*/*R1*.gz > MCF10a_PE1_rep3.\
    fastq.gz
cat 05202016-Guertin_ATACSeq-30582565/3_*/*R2*.gz > MCF10a_PE2_rep3.\
    fastq.gz
cat 05202016-Guertin_ATACSeq-30582565/5_*/*R1*.gz > MCF10a_PE1_rep5.\
    fastq.gz
cat 05202016-Guertin_ATACSeq-30582565/5_*/*R2*.gz > MCF10a_PE2_rep5.\
    fastq.gz
cat 05202016-Guertin_ATACSeq-30582565/6_*/*R1*.gz > MCF10a_PE1_rep6.\
    fastq.gz
cat 05202016-Guertin_ATACSeq-30582565/6_*/*R2*.gz > MCF10a_PE2_rep6.\
    fastq.gz
```

- Align Fastq files with bowtie2 (hg38), calculate mitochondrial fraction of reads in Sam files, and convert Sam files to sorted Bam files

```
for fq in *PE1*.fastq.gz
do
        name=$(echo $fq | awk -F"/" '{print $NF}' | awk -F"_PE" '{print\
     $1}')
        suffix=$(echo $fq | awk -F"/" '{print $NF}' | awk -F"_rep" '{\
    print $2}')
        end=$(echo $fq | awk -F"_rep" '{print $NF}' | awk -F".fastq" '{\
    print $1}')
        echo name
```

```
        echo $name
        echo suffix
        echo $suffix
        echo end
        echo $end
        echo files
        name1=$(echo ${name}_PE1_rep${suffix})
        name2=$(echo ${name}_PE2_rep${suffix})
        echo $name1
        echo $name2
        bowtie2 -p 7 -x ~/hg38 -1 $name1 -2 $name2 -S ${name}_rep${end\
    }.sam
        all=$(grep -c 'chr*' ${name}_rep${end}.sam)
        mito=$(grep -c 'chrM' ${name}_rep${end}.sam)
        echo "$all" "$mito" | awk '{print "mito%", $2 * 100/ $1}'
        samtools view -bS ${name}_rep${end}.sam > ${name}_rep${end}.bam
        rm -rf ${name}_rep${end}.sam
        samtools sort ${name}_rep${end}.bam ${name}_rep${end}.sorted
        rm -rf ${name}_rep${end}.bam
done
```

- Merge all these Bam files and make a merged Sam file.

```
for i in MCF10a_rep1.sorted.bam
do
        name=$(echo $i | awk -F"/" '{print $NF}' | awk -F"_rep" '{print\
     $1}')
        echo ${name}_rep*.sorted.bam
        samtools merge -u ${name}_merged.bam ${name}_rep*.sorted.bam
        samtools view -h -o ${name}_merged.sam ${name}_merged.bam
done
```

- Generate smooth normalized BedGraphs files for UCSC genome browser visualization and bigWig file.
  Python script samToBed.py is provided in Appendix G. Python script BedFix.py is provided in Appendix H. MCF10a_merged.bigWig generated will be used for further analysis.

```
for i in *merged.sam
do
        name=$(echo $i | awk -F"/" '{print $NF}' | awk -F".sam" '{print\
     $1}')
        echo processing $name
        python ~/samToBed.py -s $i -t con > $name.concordant.bed
        python ~/BedFix.py $name.concordant.bed $name.fixed.concordant.\
    bed
        all=$(grep -c 'chr*' $i)
        mito=$(grep -c 'chrM' $i)
        echo "$all" "$mito" | awk '{print "mito%", $2 * 100/ $1}'
        sort -k1,1 -k2,2n $name.fixed.concordant.bed | grep -v chrM > \
    $name.concordant.sorted.bed
        uniq $name.concordant.sorted.bed > $name.concordant.uniq.bed
        rm -rf $name.concordant.bed
        rm -rf $name.fixed.concordant.bed
        reads=$(wc -l $name.concordant.uniq.bed | awk -F" " '{print $1\
    }')
        norm=$(echo 10000000/$reads | bc -l | xargs printf "%.*f\n" 3)
        genomeCoverageBed -bg -scale $norm -trackline -trackopts name=\
    $name -i $name.concordant.uniq.bed -g ~/hg38_indices/hg38.chrom.\
    sizes > $name.bedGraph
```

```
        wigToBigWig $name.bedGraph ~/hg38_indices/hg38.chrom.sizes \
    $name.bigWig
        #gzip $name.bedGraph
        echo reads resulting from sequencing the same molecule
        readsinc=$(wc -l $name.concordant.sorted.bed | awk -F" " '{\
    print $1}')
        count=$((readsinc-reads))
        echo $count
        echo fraction of total concordant reads are duplicate
        frac=$(echo $count/$readsinc | bc -l | xargs printf "%.*f\n" 3)
        echo $frac
        rm -rf $name.concordant.sorted.bed
        rm -rf $name.concordant.uniq.bed
done
```

- Peak calling with Hotspot program
  Hotspot (version 4.1) is a program for identifying regions of local enrichment of short-read sequence tags mapped to genome using a binomial distribution model. Regions flagged by the algorithm are called "hotspots" (http://www.uwencode.org/proj/hotspot/) (John *et al.*, 2011). The file MCF10a_merged.tokens.txt (This file is provided in Appendix I) contains parameters (tokens) for calling hotspot. Type ./MCF10a_merged.runhotspot (This file is provided in Appendix J) in the terminal to run Hotspot. Note that you will need to edit the file paths defined in MCF10a_merged.tokens.txt to match the locations on your own file system. You will need to change paths in MCF10a_merged.runhotspot as well. MCF10a_merged.rmdup.fdr0.01.hot.bed generated by Hotspot will be used as the BroadPeak file for further analysis.

2.2 *De novo* motif analysis with MEME

Please refer to 1.4. Note that human genome hg38 was used for MCF10A data.

3 DNase composite foot printing analysis and composition conservation

Conservation scores analysis of *de novo* found motifs. *De novo* motif analysis approach has inherent biases and although we used 3-mer background frequencies in our analysis models, we cannot account for the over-representation of all k-mers in the genome and some motifs that we identify are inherently false positives. To abrogate this bias and confirm the likelihood that each motif is identified due to its importance in TF binding, we determined whether the conservation scores (as measured by phastCons Siepel *et al.* (2005) and phyloP Pollard *et al.* (2010) scores) of the motif in regulatory elements were greater than in the 1Kb surrounding area by comparing the distribution of conservation scores.

Composite footprint analysis of *de novo* found motifs. We determined whether the average composite profile of the motif in regulatory elements contained a footprint. Note that we previously showed that many TFs do not exhibit a composite footprint Sung *et al.* (2014), so this was a qualitative metric for determining TF binding and we pursued motifs without composite footprints that were evolutionary conserved.

We also will use MCF7 cells as an example here.

3.1 Retrieving raw FASTQ data sets

FASTQ files are submitted as they come off the sequencing instrument to allow for maximal decision making of downstream users (`https://genome.ucsc.edu/ENCODE/fileFormats.html#FASTQ`).

3.1.1 Obtaining naked DNase-seq single nucleotide resolution data of human fibroblast (IMR-90) cells

Download data set (SRA accession SRX247626) containing a large sample of individual, nucleotide-resolution cleavage events across the genome (Lazarovici *et al.*, 2013) and zip it.

```
curl -OL ftp://ftp-trace.ncbi.nlm.nih.gov/sra/sra-instant/reads/ByRun/sra/\
   SRR/SRR769/SRR769954/SRR769954.sra
fastq-dump SRR769954.sra
# Make the file more descriptive
mv SRR769954.fastq IMR90_Naked_DNase.fastq
gzip IMR90_Naked_DNase.fastq
```

Make a new directory "UW_DNase" for the compressed FASTQ files and move all compressed files inside.

```
mkdir ~/UW_DNase
mv IMR90_Naked_DNase.fastq.gz ~/UW\_DNase
```

3.1.2 Obtaining ENCODE FASTQ data sets for breast cancer cells

Experimental treatments for MCF7 cells were described in Section 1. We downloaded the corresponding FASTQ files.

```
curl -OL http://hgdownload.cse.ucsc.edu/goldenPath/hg19/encodeDCC/\
   wgEncodeUwDnase/wgEncodeUwDnaseMcf7Est100nm1hRawDataRep1.fastq.tgz
curl -OL http://hgdownload.cse.ucsc.edu/goldenPath/hg19/encodeDCC/\
   wgEncodeUwDnase/wgEncodeUwDnaseMcf7Est100nm1hRawDataRep2.fastq.tgz
curl -OL http://hgdownload.cse.ucsc.edu/goldenPath/hg19/encodeDCC/\
   wgEncodeUwDnase/wgEncodeUwDnaseMcf7Estctrl0hRawDataRep1.fastq.tgz
curl -OL http://hgdownload.cse.ucsc.edu/goldenPath/hg19/encodeDCC/\
   wgEncodeUwDnase/wgEncodeUwDnaseMcf7Estctrl0hRawDataRep2.fastq.tgz
```

To extract the tar.gz compressed archive, you can use the following command.

```
tar -xvf wgEncodeUwDnaseMcf7Estctrl0hRawDataRep2.fastq.tgz
tar -xvf wgEncodeUwDnaseMcf7Estctrl0hRawDataRep1.fastq.tgz
tar -xvf wgEncodeUwDnaseMcf7Est100nm1hRawDataRep2.fastq.tgz
tar -xvf wgEncodeUwDnaseMcf7Est100nm1hRawDataRep1.fastq.tgz
```

Use *cat* function to combine all FASTQ files corresponding to each sample and make the file names more descriptive. Next, compress all FASTQ files with *gzip* function.

```
cat UwStam_MCF7-DS18021*fastq > wgEncodeUwDnaseMcf7Estctrl0hRawDataRep2.\
   fastq
cat UwStam_MCF7-DS18267*fastq > wgEncodeUwDnaseMcf7Estctrl0hRawDataRep1.\
   fastq
cat UwStam_MCF7_ER-DS18025*fastq > wgEncodeUwDnaseMcf7Est100nm1hRawDataRep2\
   .fastq
cat UwStam_MCF7_ER-DS18271*fastq > wgEncodeUwDnaseMcf7Est100nm1hRawDataRep1\
   .fastq
gzip wgEncodeUwDnaseMcf7*fastq
```

Again, combine all compressed FASTQ files by *cat* and make the file name more descriptive. Then move it into the UW_DNase folder created earlier in Section 3.1.1.

```
cat wgEncodeUwDnaseMcf7*fastq.gz > UW_MCF7_both.fastq.gz
mv UW_MCF7_both.fastq.gz ~/UW_DNase
```

Additionally, save all FASTQ files in a newly created folder "wgEncodeUwDnaseMcf7Est".

```
mkdir wgEncodeUwDnaseMcf7Est
mv wgE*.fastq.gz wgEncodeUwDnaseMcf7Est
```

3.2 Aligning sequencing reads to reference hg19 genome sequences

First, we built a Bowtie (version 2.2.6) index from a set of DNA sequences by function *bowtie2-build* (`http://bowtie-bio.sourceforge.net/bowtie2/manual.shtml#the-bowtie2-build-indexer`). *bowtie2-build* outputs a set of 6 files with suffixes .1.bt2, .2.bt2, .3.bt2, .4.bt2, .rev.1.bt2, and .rev.2.bt2. These files together constitute the index and they are all that is needed to align reads to that reference.

Bowtie 2 aligns sequencing reads to long reference sequences (`http://bowtie-bio.sourceforge.net/bowtie2/manual.shtml`). Function *bowtie2* takes a Bowtie 2 index and a set of sequencing read files and outputs a set of alignments in SAM format (Langmead and Salzberg, 2012). SAM (Sequence Alignment/Map) format is a generic format for storing large nucleotide sequences alignments (Li *et al.*, 2009). Refer to SAM format specification (`http://samtools.github.io/hts-specs/SAMv1.pdf`) for more details.

```
bowtie-build hg19.fa hg19
cd ~/UW_DNase
for fq in *.fastq.gz
do
        name=$(echo $fq | awk -F"/" '{print $NF}' | awk -F".fastq.gz" '{\
    print $1}')
        echo $name
        bowtie2 -p 4 -x ~/iterative/encode/hg19 -U $fq -S $name.sam
done
```

3.3 Producing bedGraph and bigWig files from SAM output files

SAM format contains all the information needed to make a BED entry for each aligned read. Convert Sam file to Bed file using a python script written by Michael Guertin (Please address any regarding question to *mjg7y@virginia.edu*). The script "samToBed_MJG_ChIP.py" is provided in Appendix K.

genomeCoverageBed is a function provided by bedTools (`http://bedtools.readthedocs.org/en/latest/content/tools/genomecov.html`). It converts bed file to bedGraph file, allowing display of continuous-valued data in track format. bigWig files are created by *wigToBigWig* function from bedGraph files (`http://genome.ucsc.edu/goldenpath/help/bigWig.html`). We download function *wigToBigWig* at `http://hgdownload.cse.ucsc.edu/admin/exe/macOSX.x86_64/`, made this function executable and added it to the PATH variable (Appendix A).

A more efficient way would be to use `seqOutBias` to convert a bam file directly into a scaled single-nucleotide resolution bigWig file (section 4.3.1). However, this software was not developed at the time of this analysis.

```
for i in *.sam
do
```

```
        name=$(echo $i | awk -F"/" '{print $NF}' | awk -F".sam" '{print $1\
    }')
        python ~/pyscripts/samToBed_MJG_ChIP.py $i $name.bed
        sort -k1,1 -k2,2n -o $name.bed $name.bed
        genomeCoverageBed -bg -trackline -trackopts name=$name -i $name.bed\
     -g ~/iterative/hg19.chrom.sizes > $name.bedGraph
        wigToBigWig $name.bedGraph ~/hg19.chrom.sizes $name.bigWig
done
```

3.4 Retrieving conservation data sets

hg19.100way.phastCons.bw (http://hgdownload.cse.ucsc.edu/goldenpath/hg19/phastCons100way/) contains compressed phastCons scores for multiple alignments of 99 vertebrate genomes to the human genome in bigWig format. Refer to (Siepel *et al.*, 2005) for phastCons details.

hg19.100way.phyloP100way.bw (http://hgdownload.cse.ucsc.edu/goldenPath/hg19/phyloP100way/) contains conservation scores as measured by phyloP (phylogenetic p-values) from the PHAST (http://compgen.cshl.edu/phast/) package for multiple alignments of 99 vertebrate genomes to the human genome in bigWig format. Refer to (Pollard *et al.*, 2010) for phyloP details.

```
mkdir ~/conservation
cd ~/conservation
curl -OL http://hgdownload.cse.ucsc.edu/goldenpath/hg19/phastCons100way/\
    hg19.100way.phastCons.bw
curl -OL http://hgdownload.cse.ucsc.edu/goldenpath/hg19/phyloP100way/hg19\
    .100way.phyloP100way.bw
mv ~/conservation/hg19.100way.phastCons.bw ~/conservation/hg19.100way.\
    phastCons.bigWig
mv ~/conservation/hg19.100way.phyloP100way.bw ~/conservation/hg19.100way.\
    phyloP100way.bigWig
```

Note that conservation files for the hg38 assembly can be downloaded from the following weblink.

hg38.phastCons100way.bw
http://hgdownload.cse.ucsc.edu/goldenPath/hg38/phastCons100way/hg38.phastCons100way.bw

hg38.100way.phyloP100way.bw
http://hgdownload.cse.ucsc.edu/goldenPath/hg38/phyloP100way/hg38.phyloP100way.bw

3.5 Making composite plots for DNase-seq and conservation data

Composite analysis was incorporated into iteratively *de novo* motif finding analysis in section 1.4.2. In order to make composite plots, we need three kinds of input files: MAST.txt (generated in Section 1.4.2, bigWig files (generated in Section 3.3 and Section 3.4) and Hotspots_Either_E2_condition.merge.broadPeak (generated in Section 1.2.2). This process was automated using R, the script is provided in Appendix L.

4 Clustering *de novo* found regulatory sequences to unique transcription factor family

4.1 Construction of motif families based on TOMTOM and HOMER motif databases

We tailored a motif databases combining jolma2013.meme, JASPAR_CORE_REDUNDANT_2016_vertebrate.meme, uniprobe_mouse.meme and HOMER motif database (MEME databases: `http://meme-suite.org/meme-software/Databases/motifs/motif_databases.12.9.tgz`; HOMER database (downloaded Oct 20, 2015): `http://homer.salk.edu/homer/custom.motifs`). Create a new folder for individual motif meme file and run a Python script to generate individual motif files from the database constructed. The script was written by Michael Guertin (Please address any regarding question to *mjg7y@virginia.edu*). The script "MEME_individual_from_db.py" is provided in Appendix F.

Align individual motif to the motif database and produce an alignment for each significant match. An E-value of less than 0.01 was used as the threshold to denote the significance by running TOMTOM:

```
#!/bin/bash
#submit the script on the terminal
#sh ~/individual_motif.sh
dir=~/motif_databases_tomtom
subdir=individual_motif

for file in $dir/$subdir/*
do
echo $file
filename=${file%_meme.txt}
echo $filename
tomtom -no-ssc -o $filename.tomtom_output -verbosity 1 -min-overlap 5 -mi 1\
    -dist pearson -evalue -thresh 0.01 $file $dir/combined_motif_db.txt
done
```

For each motif, we got a list of its mapped motifs with rank by the following script. Their degree of match increases as the mapping distances decreases.

```
#!/bin/bash
#submit the script on the terminal
#sh ~/individual_motif_all_id.sh
dir=~/motif_databases_tomtom
subdir=individual_motif
map=mapping_list
j=1
for file in $dir/$subdir/*tomtom_output/*.txt
do
echo $file
#motifid=$(awk 'FNR == 2 {print $1}' $file)
motifid=${file%*.tomtom_output/tomtom.txt}
motifid=${motifid##*/}
echo motifid_$motifid
echo $motifid >> $dir/$map/motifidlist_$motifid.txt

linenum=$(awk 'END {print NR}' $file)
first=3
i=$first
while [[  $i -le $linenum ]]
```

```
do
head -$i $file | tail -1 > lastline
mapid[$i]=$(awk 'END {print $2}' lastline)
echo mapid[$i]_${mapid[$i]}
echo ${mapid[$i]} >> $dir/$map/motifidlist_$motifid.txt
((i = i + 1))
done
(( j = j + 1 ))
done
```

Next, we used R to build a tab delimited text file of the motif information. Each line corresponds to a motif and its partners.

```
file_list <- list.files()
for (file in file_list){
# if the merged dataset doesn't exist, create it
if (!exists("dataset")){
dataset <- read.table(file, header=F,stringsAsFactors = F)
}

# if the merged dataset does exist, append to it
if (exists("dataset")){
temp_dataset <-read.table(file, header=F,stringsAsFactors = F)
dataset<-c(dataset, temp_dataset)
rm(temp_dataset)
}
}

dataset=dataset[-1]

length.dataset = length(dataset)

i=1
motif.length=1
for (i in 1:length(dataset))
{
motif.length=max(motif.length,length(as.character(unlist(dataset[i]))))
}
print(motif.length)

dataset.df = data.frame(matrix('a', ncol = motif.length, nrow = 1),\
    stringsAsFactors=F)

i=1
for (i in 1:length(dataset))
{
singledata=as.character(unlist(dataset[i]))
length(singledata)=motif.length
dataset.df=rbind(dataset.df,singledata)
}

dataset.df=dataset.df[-1,]

write.table(dataset.df,"maplist.txt",quote=F,sep="\t",row.names=F,col.names\
    =F,na='')
```

Filter out anything that is Arabidopsis Drosophila, cElegans, SacCer, SeqBias, (in the row name).

```
grep -v 'Drosophila\|Arabidopsis\|SacCer\|cElegans\|SeqBias' $dir/maplist.\
    txt > $dir/maplist_subset.txt
```

4.1.1 Construction of motif family with members connecting to each other or through the other motifs

We define a motif family as the motifs connecting directly to each other or through the other motifs. An edge was inferred between two motif nodes if their similarity exceeded a negative $\log_{10}$ E-value of 10. As shown in Figure 1, each family is denoted with a different color from the other motif families. All the motifs in one family are connected to each other or through the other motifs in the family. The size of the node is proportional to the number of nodes connecting to this node. The width of each edge denotes the connectivity ($-log10E.val$) between two nodes. We can identify all the motif family using a python script: "TOMTOM_motif_families_nonrecursive.py" is provided in Appendix M.

4.1.2 Construction of motif families by community detection algorithm

As shown in Figure 2, each motif family could be further divided into different communities by "Fast Modularity" Community Structure Inference Algorithm (Clauset *et al.*, 2004). First, generate a three-column file as the input for this algorithm:

```
dir=~/motif_database_tomtom/individual_motif
for file in $dir/*
do
cut -f1,2,5 $file/tomtom.txt >> 3_col_combined_motif_db_pre.txt
done
grep -v '#' 3_col_combined_motif_db_pre.txt > 3_col_combined_motif_db.txt
rm 3_col_combined_motif_db_pre.txt
```

The first column refers to the all the motifs in our database. The motif in the second column is mapped to the motif in the first column at each row, the significant of the mapping is denoted by an E-value (obtained from TOMTOM) displaying in the third column. Further, we can use `R` (coded is attached below) to obtain the communities using the three-column file as the input file. The output file is a text file, each row corresponds to a community.

```
rm(list=ls())
setwd("~/visualization_motif_families")
threecol=read.csv("3_col_combined_motif_db.txt",
header=F,stringsAsFactors = F,sep='\t')
colnames(threecol)=c('from','to','e_value')
threecol$weight=abs(log(threecol$e_value))
library(igraph)
#create the graph variable
g=graph.data.frame(threecol,directed=F)
g=simplify(g)
#commnity detection
comm.g = fastgreedy.community(g)

#save the motif communites to a text file
length=1
for (i in 1:length(comm.g))
{
length=max(length,length(as.character(unlist(comm.g[i]))))
}
print(length)
```

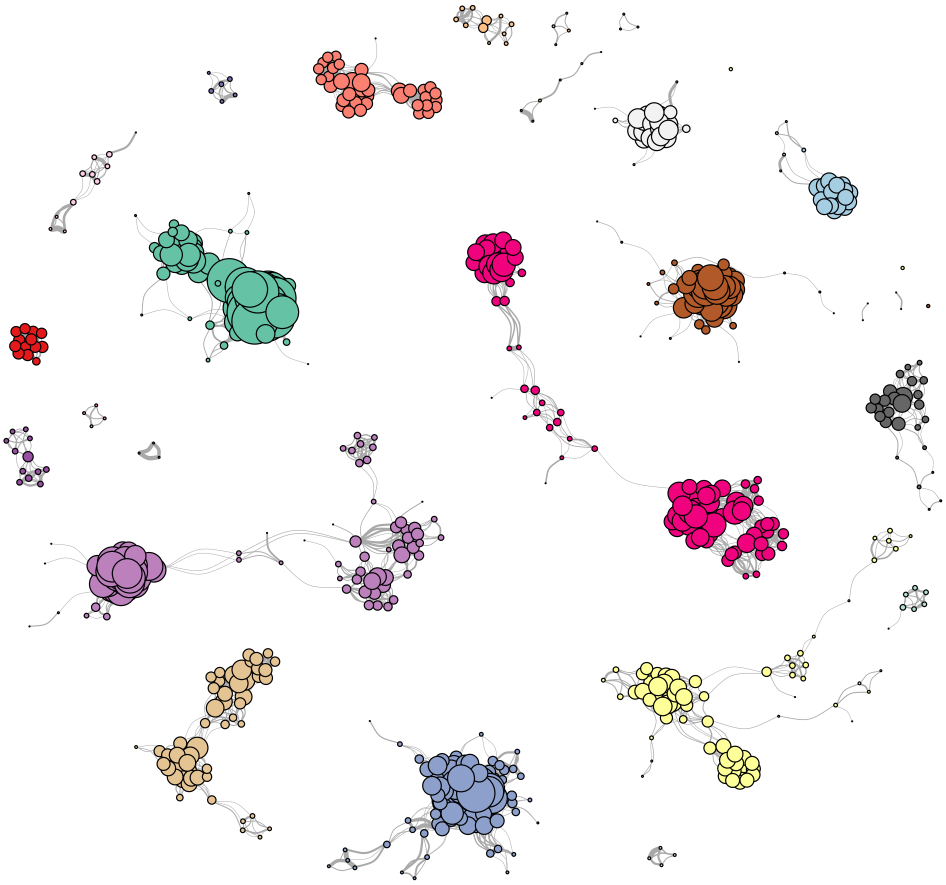

Figure 1: Visualization of 28 motif families

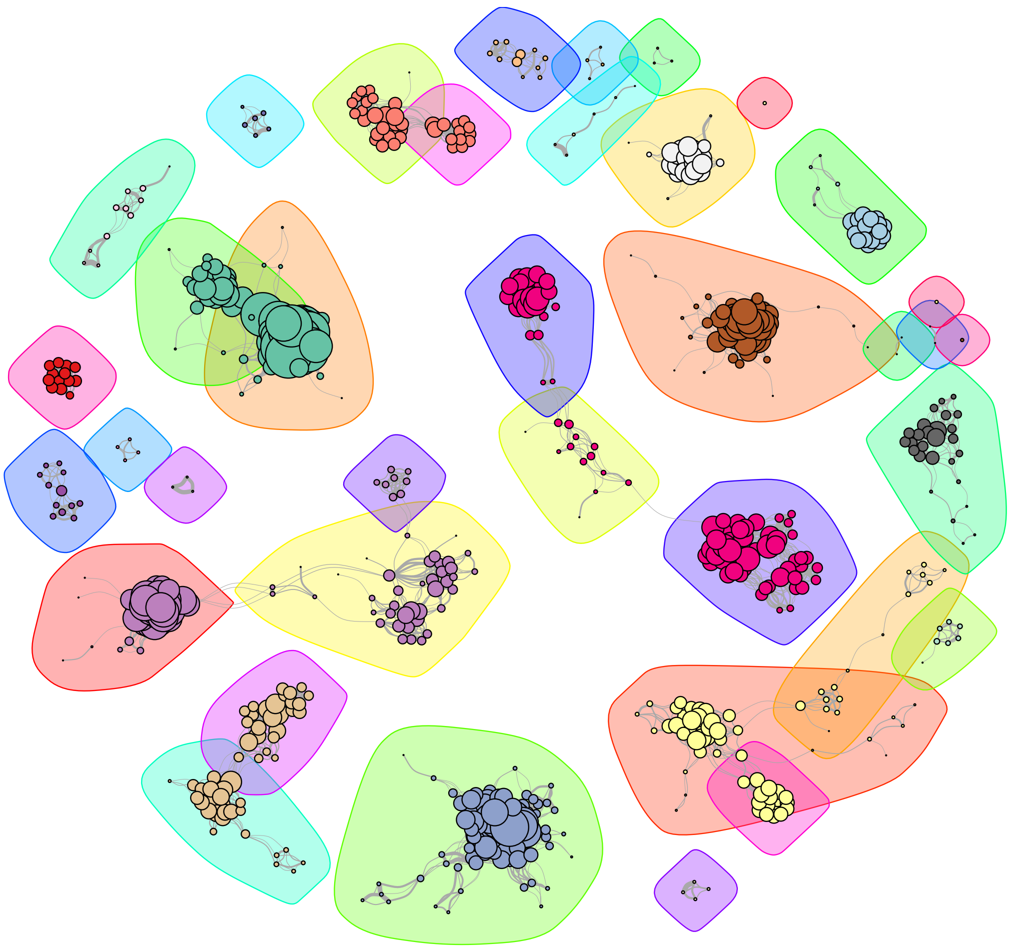

Figure 2: Visualization of 37 motif communities

```
cluster.df = data.frame(matrix('a', ncol = length, nrow = 1),\
    stringsAsFactors=F)
for (i in 1:length(comm.g))
{
community=as.character(unlist(comm.g[i]))
length(community)=length
cluster.df=rbind(cluster.df,community)
}
cluster.df=cluster.df[-1,]
write.table(cluster.df,"motif_405_communities_combined_motif_db.txt",quote=\
    F,sep="\t",row.names=F,col.names=F,na='')
```

4.2 Identification of unique transcription factor families for each cell line

We rerun TOMTOM with a threshold of E-value less than 0.01 for each cell lines and get a new TOMTOM output folder, a motif list, an orphan motif list, and the corresponding motif families/communities list. Orphan motif refers to the *de novo* found motif, which is not occurring in our motif database (the motif databases was constructed in section 4.1). We describe in more detail about how to identify and verify orphan motifs in the next subsection 4.3.

```
celine=<name of the cell line in upper case>
celine_lc=<name of the cell line in lower case>
dir=~/Desktop/Iterate_${celine}_hotspot_onestop/iteration
#run the script:
#sh $dir/rerun_tomtom_${celine_lc}.sh

tomtomdatabase=~/combined_motif_db.txt
family=~/motif_405_communities_combined_motif_db.txt
j=1

cd ~
#########onestop_iteration#########
withmotif=$dir/withmotif
for file in $withmotif/*
do
echo $file
before=${file%.txt}
tomtom -verbosity 1 -o $before.tomtom_output -min-overlap 5 -dist pearson -\
    evalue -thresh 0.01 -no-ssc $file $tomtomdatabase
tomtomtxt=$before.tomtom_output/tomtom.txt
motifid=$(awk 'FNR == 2 {print $2}' $tomtomtxt)
echo motifid_$motifid

file1=${file#*minimal_meme_r}
num=${file1%%_*}

if [[ $motifid == "" ]]
then
motifid=orphan$j
echo motifid_$motifid
echo minimal_meme_r${num}_${motifid} >> $withmotif/orphans.txt
((j = j + 1))
else
echo motifid_$motifid
echo $motifid >> $withmotif/motifs.txt
fi
```

```
newfile=minimal_meme_r${num}_${motifid}.txt
mv $file $withmotif/$newfile

for file in $before.tomtom_output/*
do
file1=${file%.*}
file2=${file##*.}
mv $file ${file1}_r${num}_${motifid}.${file2}
done
done
cat $withmotif/motifs.txt | while read LINE; do grep -n $LINE $family | cut\
     -d : -f 1 >> $withmotif/motifs_family.txt; done

mkdir $withmotif/minimal_meme_orphan
mv $withmotif/minimal_meme*orphan*.txt $withmotif/minimal_meme_orphan/

for file in $withmotif/minimal_meme*.txt
do
filenametxt=${file#${withmotif}/}
filename=${filenametxt%.txt}
mkdir $dir/mast_files_for_conservation_plots/${filename}.mast_output
mast $file $dir/inten_highest_hotspot.fasta -hit_list -m 1 > $dir/\
    mast_files_for_conservation_plots/${filename}.mast_output/\
    mast_hotspots_${filename}.txt
python ~/iterative/scripts/MAST_bed_conversion.py -i $dir/\
    mast_files_for_conservation_plots/${filename}.mast_output/\
    mast_hotspots_${filename}.txt
done

############mememchip-dreme
dreme=$dir/memechip/memechip_dreme_individual
for file in $dreme/*
do
echo $file
before=${file%.txt}
tomtom -verbosity 1 -o $before.tomtom_output -min-overlap 5 -dist pearson -\
    evalue -thresh 0.01 -no-ssc $file $tomtomdatabase
tomtomtxt=$before.tomtom_output/tomtom.txt
motifid=$(awk 'FNR == 2 {print $2}' $tomtomtxt)
echo motifid_$motifid

if [[ $motifid == "" ]]
then
motifid=orphan$j
echo motifid_$motifid
echo ${motifid} >> $dreme/orphans.txt
((j = j + 1))
else
echo motifid_$motifid
echo $motifid >> $dreme/motifs.txt
fi

newfile=motif_${motifid}_dreme.txt
mv $file $dreme/$newfile

for file in $before.tomtom_output/*
do
file1=${file%.*}
```

```
file2=${file##*.}
mv $file ${file1}_${motifid}_dreme.${file2}
done
done
cat $dreme/motifs.txt | while read LINE; do grep -n $LINE $family | cut -d \
    : -f 1 >> $dreme/motifs_family.txt; done

mkdir $dreme/motif_orphans_dreme
mv $dreme/motif*orphan*dreme.txt $dreme/motif_orphans_dreme

for file in $dreme/motif*dreme.txt
do
filenametxt=${file#${dreme}/}
filename=${filenametxt%.txt}
mkdir $dir/mast_files_for_conservation_plots/${filename}.mast_output
mast $file $dir/inten_highest_hotspot.fasta -hit_list -m 1 > $dir/\
    mast_files_for_conservation_plots/${filename}.mast_output/\
    mast_hotspots_${filename}.txt
python ~/iterative/scripts/MAST_bed_conversion.py -i $dir/\
    mast_files_for_conservation_plots/${filename}.mast_output/\
    mast_hotspots_${filename}.txt
done

###########meme-chip-meme
meme=$dir/memechip/memechip_meme_individual
for file in $meme/*
do
echo $file
before=${file%.txt}
tomtom -verbosity 1 -o $before.tomtom_output -min-overlap 5 -dist pearson -\
    evalue -thresh 0.01 -no-ssc $file $tomtomdatabase
tomtomtxt=$before.tomtom_output/tomtom.txt
motifid=$(awk 'FNR == 2 {print $2}' $tomtomtxt)
echo motifid_$motifid

if [[ $motifid == "" ]]
then
motifid=orphan$j
echo motifid_$motifid
echo ${motifid} >> $meme/orphans.txt
((j = j + 1))
else
echo motifid_$motifid
echo $motifid >> $meme/motifs.txt
fi

newfile=motif_${motifid}_meme.txt
mv $file $meme/$newfile

for file in $before.tomtom_output/*
do
file1=${file%.*}
file2=${file##*.}
mv $file ${file1}_${motifid}_meme.${file2}
done
done
cat $meme/motifs.txt | while read LINE; do grep -n $LINE $family | cut -d :\
     -f 1 >> $meme/motifs_family.txt; done
```

```
mkdir $meme/motif_orphans_meme
mv $meme/motif*orphan*meme.txt $meme/motif_orphans_meme

for file in $meme/motif*meme.txt
do
filenametxt=${file#${meme}/}
filename=${filenametxt%.txt}
mkdir $dir/mast_files_for_conservation_plots/${filename}.mast_output
mast $file $dir/inten_highest_hotspot.fasta -hit_list -m 1 > $dir/\
    mast_files_for_conservation_plots/${filename}.mast_output/\
    mast_hotspots_${filename}.txt
python ~/iterative/scripts/MAST_bed_conversion.py -i $dir/\
    mast_files_for_conservation_plots/${filename}.mast_output/\
    mast_hotspots_${filename}.txt
done
```

The following bash code will identify the unique TF community numbers found in each cell line. It will also report the number of unique TF communities across all five BrCa cell lines.

```
#!/bin/bash

#submit the script on the terminal
#bash ~/motif_communities_5_brca_celines.sh

dir=~/visualization_motif_families

#hmec
hmec_onestop=~/Desktop/Iterate_HMEC_hotspot_onestop/iteration/withmotif/\
    motifs.txt
hmec_memechipmeme=~/Desktop/Iterate_HMEC_hotspot_onestop/iteration/memechip\
    /memechip_meme_individual/motifs.txt
hmec_memechipdreme=~/Desktop/Iterate_HMEC_hotspot_onestop/iteration/\
    memechip/memechip_dreme_individual/motifs.txt
#mcf7
mcf7_onestop=~/Desktop/Iterate_MCF7_hotspot_onestop/iteration/withmotif/\
    motifs.txt
mcf7_memechipmeme=~/Desktop/Iterate_MCF7_hotspot_onestop/iteration/memechip\
    /memechip_meme_individual/motifs.txt
mcf7_memechipdreme=~/Desktop/Iterate_MCF7_hotspot_onestop/iteration/\
    memechip/memechip_dreme_individual/motifs.txt
#t47d
t47d_onestop=~/Desktop/Iterate_T47D_hotspot_onestop/iteration/withmotif/\
    motifs.txt
t47d_memechipmeme=~/Desktop/Iterate_T47D_hotspot_onestop/iteration/memechip\
    /memechip_meme_individual/motifs.txt
t47d_memechipdreme=~/Desktop/Iterate_T47D_hotspot_onestop/iteration/\
    memechip/memechip_dreme_individual/motifs.txt
#vhmec
vhmec_onestop=~/Desktop/Iterate_vHMEC_hotspot_onestop/iteration/withmotif/\
    motifs.txt
vhmec_memechipmeme=~/Desktop/Iterate_vHMEC_hotspot_onestop/iteration/\
    memechip/memechip_meme_individual/motifs.txt
vhmec_memechipdreme=~/Desktop/Iterate_vHMEC_hotspot_onestop/iteration/\
    memechip/memechip_dreme_individual/motifs.txt
#mcf10a
mcf10a_onestop=~/Desktop/Iterate_MCF10A_hotspot_onestop/iteration/withmotif\
    /motifs.txt
mcf10a_memechipmeme=~/Desktop/Iterate_MCF10A_hotspot_onestop/iteration/\
    memechip/memechip_meme_individual/motifs.txt
```

```
mcf10a_memechipdreme=~/Desktop/Iterate_MCF10A_hotspot_onestop/iteration/\
    memechip/memechip_dreme_individual/motifs.txt

sort -u ${hmec_onestop} ${hmec_memechipmeme} ${hmec_memechipdreme} | grep -\
    v -e '^$' > $dir/hmec_unique_motifs.txt
sort -u ${mcf7_onestop} ${mcf7_memechipmeme} ${mcf7_memechipdreme} | grep -\
    v -e '^$' > $dir/mcf7_unique_motifs.txt
sort -u ${t47d_onestop} ${t47d_memechipmeme} ${t47d_memechipdreme} | grep -\
    v -e '^$' > $dir/t47d_unique_motifs.txt
sort -u ${vhmec_onestop} ${vhmec_memechipmeme} ${vhmec_memechipdreme} | \
    grep -v -e '^$' > $dir/vhmec_unique_motifs.txt
sort -u ${mcf10a_onestop} ${mcf10a_memechipmeme} ${mcf10a_memechipdreme} | \
    grep -v -e '^$' > $dir/mcf10a_unique_motifs.txt

#merge all the unique motifs found together in five cell lines
sort -u $dir/hmec_unique_motifs.txt $dir/mcf7_unique_motifs.txt  $dir/\
    t47d_unique_motifs.txt $dir/vhmec_unique_motifs.txt $dir/\
    mcf10a_unique_motifs.txt > $dir/5_brca_celines_unique_motifs.txt

###find the corresponding communities
com_405=/~/motif_405_communities_combined_motif_db.txt

#5 BrCa cell lines
grep -n -f $dir/5_brca_celines_unique_motifs.txt $com_405 | cut -d : -f 1 >\
     $dir/5_brca_celines_405communities_num.txt

#hmec
grep -n -f $dir/hmec_unique_motifs.txt $com_405 | cut -d : -f 1 > $dir/\
    hmec_405communities_num.txt

#mcf7
grep -n -f $dir/mcf7_unique_motifs.txt $com_405 | cut -d : -f 1 > $dir/\
    mcf7_405communities_num.txt

#t47d
#get the corresponding communities number out of the original 405 community
grep -n -f $dir/t47d_unique_motifs.txt $com_405 | cut -d : -f 1 > $dir/\
    t47d_405communities_num.txt

#vhmec
grep -n -f $dir/vhmec_unique_motifs.txt $com_405 | cut -d : -f 1 > $dir/\
    vhmec_405communities_num.txt

#mcf10a
#get the corresponding communities number out of the original 405 community
grep -n -f $dir/mcf10a_unique_motifs.txt $com_405 | cut -d : -f 1 > $dir/\
    mcf10a_405communities_num.txt
```

Then, we visualize the TF families and communities using R, as shown in Figure 1 and Figure 2. Note that "3_col_combined_motif_db.txt" was produced in Section 4.1.2.

```
rm(list=ls())
setwd("~/visualization_motif_families_151207_db")

threecol=read.csv('3_col_combined_motif_db.txt',
header=F,stringsAsFactors = F,sep='\t')
colnames(threecol)=c('from','to','e_value')
threecol$weight=abs(log(threecol$e_value))
```

```
library(igraph)
#create the graph variable
g=graph.data.frame(threecol,directed=F)
g=simplify(g)

cluster=clusters(g)

#next
comm.g = fastgreedy.community(g)

#plot 36 communities only
i=read.table("5_brca_celines_405communities_num.txt")
i=as.numeric(t(i))
j <- comm.g$membership %in% i
g1 = subgraph(g,V(g)[j])

#note: please change the number of colors depending on how many of \
    communities you got
mycol=c("#7570B3", "#E5C494", "#FB8072", "#B15928", "#F2F2F2", "#E5D8BD", "\
    #FFFF99", "#A65628", "#FFD92F", "#66C2A5", "#BC80BD", "#8DA0CB",
"#FC8D62", "#FED9A6", "#FCCDE5", "#FFFF99", "#666666", "#B3E2CD", "#984EA3"\
    , "#FDBF6F", "#F781BF", "#F0027F", "#CCCCCC", "#A6CEE3",
"#FFFFB3", "#FDC086", "#E31A1C", "#D9D9D9", "#6A3D9A", "#999999", "#D95F02"\
    , "#7FC97F", "#FF7F00", "#A6D854", "#BF5B17", "#FFED6F",
"#A6761D", "#E78AC3","#FFFF33")

l=layout.fruchterman.reingold(g1)
l=layout.norm(l,-1,1,-1,1)
cluster1=clusters(g1)
#note that the communities increase to 39 after sub-graphing
comm.g1 = fastgreedy.community(g1)

length(comm.g1)#returns the number of communities
sizes(comm.g1)#returns the community sizes, in the order of their ids
membership(comm.g1)#gives the division of the vertices, into communities.

#save the motif communites to a text file
length=1
for (i in 1:length(comm.g1))
{
length=max(length,length(as.character(unlist(comm.g1[i]))))
}
print(length)

cluster.df = data.frame(matrix('a', ncol = length, nrow = 1),\
    stringsAsFactors=F)
for (i in 1:length(comm.g1))
{
community=as.character(unlist(comm.g1[i]))
length(community)=length
cluster.df=rbind(cluster.df,community)
}

cluster.df=cluster.df[-1,]

write.table(cluster.df,"5_brca_celines_39_communities_newly_clustered.txt",\
    quote=F,sep="\t",row.names=F,col.names=F,na='')

pdf(paste0('family','_all_no_comm','.pdf'),width=10,height=10)
```

```
plot(g1,layout=l,rescale=F,vertex.label.cex=.5,xlim=range(l[,1]),  ylim=\
   range(l[,2]),
edge.width=E(g1)$weight/20,vertex.size=degree(g1,mode='out')/5,
edge.curved=T,vertex.label=NA,vertex.color=mycol[cluster1$membership],
margin=0,asp=0)
dev.off()

pdf(paste0('family','_all_commu','.pdf'),width=10,height=10)
plot(g1,layout=l,rescale=F,vertex.label.cex=.5,xlim=range(l[,1]),  mark.\
   groups = communities(comm.g1), ylim=range(l[,2]),
edge.width=E(g1)$weight/20,vertex.size=degree(g1,mode='out')/5,
edge.curved=T,vertex.label=NA,vertex.color=mycol[cluster1$membership],
margin=0,asp=0)
dev.off()

detach(igraph)
```

4.3 Identification of orphan motifs

4.3.1 Normalize bigWig files - *seqOutBias*

Compilation and Installation of *seqOutBias*

```
#install the most recent rust version
curl -sSf https://static.rust-lang.org/rustup.sh | sh
#install genometools
brew install homebrew/science/genometools
#unzip seqOutBias
tar xzf seqOutBias_1.0.1_src.tgz
#compile
cd seqOutBias_1.0.1
cargo build --release
```

seqOutBias program, developed by André Martins and Michael Guertin (Please address any regarding questions to *mjg7y@virginia.edu*), is used to normalize sequence biases resulting from enzymatic sequence cut preferences. Please refer to the vignette of *seqOutBias* program for detailed installation and usage of this program. Note that rust, *wigToBigWig* utility, and genometools are pre-requested. To install Rust and Cargo, visit `https://www.rust-lang.org/en-US/`, *seqOutBias* should compile with Rust 1.11.0 or later. You'll also need to install genome tools (`http://genometools.org/`), following the instruction on that page. You will also need to build the wigToBigWig utility from the UCSC Genome Browser source code (`http://genome.ucsc.edu/admin/git.html`), following the instructions included with the Genome Browser source code. This command is required to obtain bigWig files after scaling. After compilation, copy the *seqOutBias* binary (~/seqOutBias_1.0.1/target/release/seqOutBias) to a folder in your PATH, for example /usr/local/bin.

Normalize bigWig files

```
#bash ~/Desktop/normalized_bigWig/enzcut_normalize_bigwig.sh
#note that all the bam file needs to be sorted by coordinates first!!!!!

cd ~/Desktop/normalized_bigWig/
#normalize IMR90_Naked_DNase
seqOutBias ~/iterative/hg19.fa ~/UW_DNase/UW_DNase_MCF7/IMR90_Naked_DNase.\
   bam --kmer-size=6 --bw=Naked_DNA_6-mer.bigWig --plus-offset=3 --minus-\
   offset=3 --shift-counts --skip-bed
```

```
#normalize MCF7
seqOutBias ~/iterative/hg19.fa ~/UW_DNase/UW_DNase_MCF7/UW_MCF7_both.bam --\
    kmer-size=6 --bw=MCF7_Chromatin_6-mer.bigWig --plus-offset=3 --minus-\
    offset=3 --shift-counts --skip-bed

#normalize T47D
#covert sam file to bam file
#reference: http://davetang.org/wiki/tiki-index.php?page=SAMTools#\
    Converting_a_SAM_file_to_a_BAM_file
samtools view -Sb ~/UW_DNase_T47D/UW_T47D.sam > ~/UW_DNase_T47D/UW_T47D.bam
#first check the bam file is sorted or not
samtools view -H ~/UW_DNase/UW_DNase_T47D/UW_T47D.bam
#since it is also unsorted, sort the bam file first
samtools sort ~/UW_DNase/UW_DNase_T47D/UW_T47D.bam ~/UW_DNase/UW_DNase_T47D\
    /UW_T47D.sorted
#seqOutBias
seqOutBias ~/iterative/hg19.fa ~/UW_DNase/UW_DNase_T47D/UW_T47D.sorted.bam \
    --kmer-size=6 --bw=T47D_Chromatin_6-mer.bigWig --plus-offset=3 --minus-\
    offset=3 --shift-counts --skip-bed

#normalize HMEC
#covert sam file to bam file
samtools view -Sb ~/UW_DNase/UW_DNase_HMEC/hmec.sam > ~/UW_DNase/\
    UW_DNase_HMEC/hmec.bam
#first check the bam file is sorted or not
samtools view -H ~/UW_DNase/UW_DNase_HMEC/hmec.bam
#since it is also unsorted, sort the bam file first
samtools sort ~/UW_DNase/UW_DNase_HMEC/hmec.bam ~/UW_DNase/UW_DNase_HMEC/\
    hmec.sorted
#seqOutBias
seqOutBias ~/iterative/hg19.fa ~/UW_DNase/UW_DNase_HMEC/hmec.sorted.bam --\
    kmer-size=6 --bw=HMEC_Chromatin_6-mer.bigWig --plus-offset=3 --minus-\
    offset=3 --shift-counts --skip-bed

#normalize vHMEC
#covert sam file to bam file
samtools view -Sb ~/UW_DNase/UW_DNase_vHMEC/vhmec_conservation.sam > ~/\
    UW_DNase/UW_DNase_vHMEC/vhmec_conservation.bam
#first check the bam file is sorted or not
samtools view -H ~/UW_DNase/UW_DNase_vHMEC/vhmec_conservation.bam
#since it is also unsorted, sort the bam file first
samtools sort ~/UW_DNase/UW_DNase_vHMEC/vhmec_conservation.bam ~/UW_DNase/\
    UW_DNase_vHMEC/vhmec_conservation.sorted
#seqOutBias
seqOutBias ~/iterative/hg19.fa ~/UW_DNase/UW_DNase_vHMEC/vhmec_conservation\
    .sorted.bam --kmer-size=6 --bw=vHMEC_Chromatin_6-mer.bigWig --plus-\
    offset=3 --minus-offset=3 --shift-counts --skip-bed

#normalize MCF10A
#use seqOutBias_1.0.1_src.tgz, updated on 160831
#naked DNA
cd ~/hg38_seqOutBias
seqOutBias hg38.fa IMR90_Naked_DNase.bam --kmer-size=6 --bw=Naked_DNA_6-mer\
    .bigWig --plus-offset=3 --minus-offset=3 --shift-counts --skip-bed
MCF10A=~/Desktop/Iterate_MCF10A_hotspot/orginial_files_ninadnw
seqOutBias hg38.fa $MCF10A/MCF10a_rep1.sorted.bam  $MCF10A/MCF10a_rep2.\
    sorted.bam $MCF10A/MCF10a_rep3.sorted.bam $MCF10A/MCF10a_rep5.sorted.bam\
     $MCF10A/MCF10a_rep6.sorted.bam --kmer-mask NXNXXNXNXNXCNXNXXXNNN --bw=\
    MCF10A_Chromatin_NXNXXNXNXNXCNXNXXXNNN-mer.bigWig --shift-counts --skip-\
```

```
    bed --pdist=50:400 --only-paired --read-size=76
```

4.3.2 Make conservation plot and DNase footprint plot for the orphan motifs

A text file in MEME minimal motif format was generated for all the candidate orphans (motifs with TOMTOM E-value larger than 1.0) found through MEME one-stop iteration and MEME-ChIP step. First, make mast bed file from the MEME minimal motif text files. In this script, "MAST_bed_conversion.py" is provided in Appendix D.

```
#bash ~/Desktop/Iterate_MCF7_hotspot_onestop/iteration/\
    generate_mast_bed_orphan_mcf7.sh
celine=MCF7
celine_lc=mcf7
dir=~/Desktop/Iterate_${celine}_hotspot_onestop/iteration
withmotif=$dir/withmotif

mkdir $dir/mast_files_for_conservation_plots_orphan
#mast for the orphans found by onestop iteration
for file in $withmotif/minimal_meme_orphan/minimal_meme*.txt
do
filenametxt=${file#${withmotif}/minimal_meme_orphan/}
filename=${filenametxt%.txt}
echo $filename
mkdir $dir/mast_files_for_conservation_plots_orphan/${filename}.mast_output
mast $file $dir/inten_highest_hotspot.fasta -hit_list -m 1 > $dir/\
    mast_files_for_conservation_plots_orphan/${filename}.mast_output/\
    mast_hotspots_${filename}.txt
python ~/iterative/scripts/MAST_bed_conversion.py -i $dir/\
    mast_files_for_conservation_plots_orphan/${filename}.mast_output/\
    mast_hotspots_${filename}.txt
done

#mast for the orphans found by memechip-dreme
dreme=$dir/memechip/memechip_dreme_individual
for file in $dreme/motif_orphans_dreme/motif*dreme.txt
do
filenametxt=${file#${dreme}/motif_orphans_dreme/}
filename=${filenametxt%.txt}
echo $filename
mkdir $dir/mast_files_for_conservation_plots_orphan/${filename}.mast_output
mast $file $dir/inten_highest_hotspot.fasta -hit_list -m 1 > $dir/\
    mast_files_for_conservation_plots_orphan/${filename}.mast_output/\
    mast_hotspots_${filename}.txt
python ~/iterative/scripts/MAST_bed_conversion.py -i $dir/\
    mast_files_for_conservation_plots_orphan/${filename}.mast_output/\
    mast_hotspots_${filename}.txt
done

#mast for the orphans found by memechip-meme
meme=$dir/memechip/memechip_meme_individual
for file in $meme/motif_orphans_meme/motif*meme.txt
do
filenametxt=${file#${meme}/motif_orphans_meme/}
filename=${filenametxt%.txt}
echo $filename
mkdir $dir/mast_files_for_conservation_plots_orphan/${filename}.mast_output
mast $file $dir/inten_highest_hotspot.fasta -hit_list -m 1 > $dir/\
    mast_files_for_conservation_plots_orphan/${filename}.mast_output/\
```

```
    mast_hotspots_${filename}.txt
python ~/iterative/scripts/MAST_bed_conversion.py -i $dir/\
    mast_files_for_conservation_plots_orphan/${filename}.mast_output/\
    mast_hotspots_${filename}.txt
done

#delete mast folder containing a size 0 bed file
find $dir/mast_files_for_conservation_plots_orphan -size 0 | while read \
    line; do folder=${line%/mast_hotspots*}&& echo $folder && rm -r $folder;\
     done
```

Once mast bed files are generated for the candidate orphan motifs, we can make conservation plots and composite DNase footprint plots. See the script in Appendix N. The input files are a folder containing all the orphan mast files ('~/Desktop/Iterate_MCF7_hotspot_onestop/iteration/mast_files_for_conservation_plots_orphan'), a folder containing the normalized bigWig file for both chromatin and naked DNA ('~/Desktop/normalized_bigWig/normalized_bigwig/MCF7', produced in 4.3.1), and merged hotspot file ('~/iterative/Hotspots_Either_E2_condition.merge.broadPeak', produced in 1.2.2).

Scrambled orphan motifs

Randomly shuffle the information content for every nucleotide in each orphan motif, and the output MEME minimal motif is defined as a **scrambled orphan motif**. We randomly scrambled each orphan motif for 20 times. Python script "meme_scramble_order.py" is used to scramble the motif, provided in Appendix O. And then generate the mast bed file for all the scrambled orphan motif. In this script, "MAST_bed_conversion.py" is provided in Appendix D.

```
#bash ~/Desktop/Iterate_MCF7_hotspot_onestop/iteration/\
    generate_scramble_mast_bed_orphan_mcf7.sh
celine=MCF7
celine_lc=mcf7
dir=~/Desktop/Iterate_${celine}_hotspot_onestop/iteration
withmotif=$dir/withmotif

mkdir $dir/scramble_mast_files_for_conservation_plots_orphan
#mast for the orphans found by onestop iteration
for file in $withmotif/minimal_meme_orphan/minimal_meme*.txt
do
filenametxt=${file#${withmotif}/minimal_meme_orphan/}
filename=${filenametxt%.txt}
echo $filename
python ~/iterative/scripts/meme_scramble_order.py -i $file
mkdir $dir/scramble_mast_files_for_conservation_plots_orphan/${filename}\
    _scramble.mast_output
mast $withmotif/minimal_meme_orphan/${filename}_scramble.txt $dir/\
    inten_highest_hotspot.fasta -hit_list -m 1 > $dir/\
    scramble_mast_files_for_conservation_plots_orphan/${filename}_scramble.\
    mast_output/mast_hotspots_${filename}_scramble.txt
python ~/iterative/scripts/MAST_bed_conversion.py -i $dir/\
    scramble_mast_files_for_conservation_plots_orphan/${filename}_scramble.\
    mast_output/mast_hotspots_${filename}_scramble.txt
done

#mast for the orphans found by memechip-dreme
dreme=$dir/memechip/memechip_dreme_individual
for file in $dreme/motif_orphans_dreme/motif*dreme.txt
do
filenametxt=${file#${dreme}/motif_orphans_dreme/}
filename=${filenametxt%.txt}
echo $filename
```

```
python ~/iterative/scripts/meme_scramble_order.py -i $file
mkdir $dir/scramble_mast_files_for_conservation_plots_orphan/${filename}\
    _scramble.mast_output
mast $dreme/motif_orphans_dreme/${filename}_scramble.txt $dir/\
    inten_highest_hotspot.fasta -hit_list -m 1 > $dir/\
    scramble_mast_files_for_conservation_plots_orphan/${filename}_scramble.\
    mast_output/mast_hotspots_${filename}_scramble.txt
python ~/iterative/scripts/MAST_bed_conversion.py -i $dir/\
    scramble_mast_files_for_conservation_plots_orphan/${filename}_scramble.\
    mast_output/mast_hotspots_${filename}_scramble.txt
done

#mast for the orphans found by memechip-meme
meme=$dir/memechip/memechip_meme_individual
for file in $meme/motif_orphans_meme/motif*meme.txt
do
filenametxt=${file#${meme}/motif_orphans_meme/}
filename=${filenametxt%.txt}
echo $filename
python ~/iterative/scripts/meme_scramble_order.py -i $file
mkdir $dir/scramble_mast_files_for_conservation_plots_orphan/${filename}\
    _scramble.mast_output
mast $meme/motif_orphans_meme/${filename}_scramble.txt $dir/\
    inten_highest_hotspot.fasta -hit_list -m 1 > $dir/\
    scramble_mast_files_for_conservation_plots_orphan/${filename}_scramble.\
    mast_output/mast_hotspots_${filename}_scramble.txt
python ~/iterative/scripts/MAST_bed_conversion.py -i $dir/\
    scramble_mast_files_for_conservation_plots_orphan/${filename}_scramble.\
    mast_output/mast_hotspots_${filename}_scramble.txt
done

#delete mast folder containing a size 0 bed file
find $dir/scramble_mast_files_for_conservation_plots_orphan -size 0 | while\
     read line; do folder=${line%/mast_hotspots*}&& echo $folder && rm -r \
    $folder; done
```

We can make conservation plots with the input scrambled mast bed files. The script is provided in Appendix P. Merge the conservation plots for the original orphan motifs and the scramble ones, and plot the them side by side in R.

```
#Rscript ~/Desktop/normalized_bigWig/conservation_composite_footprint/MCF7/\
    orphan/scramble/merge_original_scramble.R
celine='MCF7'
dir=paste0("~/Desktop/normalized_bigWig/conservation_composite_footprint/",
celine,
"/orphan/scramble")
setwd(dir)
load(paste0(celine,
"_conservation_composite_footprint_orphan_scramble.RData"))
all.composites.conservation.scramble=all.composites.conservation
all.composites.dnase.scramble=all.composites.dnase
rm(all.composites.conservation,all.composites.dnase)
load(paste0("~/Desktop/normalized_bigWig/conservation_composite_footprint/"\
    ,
celine,"/orphan/",celine,
"_conservation_composite_footprint_orphan.RData"))
conservation=rbind2(all.composites.conservation,all.composites.conservation\
    .scramble)
conservation.sort=conservation[order(conservation$grp),]
```

```
composites.func.panels.conservation.x(conservation.sort, fact = 'merge', \
   summit = 'Motif', num=25)
#create two conservation plots, because the original one is too long, that \
   illustrator cannot read all of those plots at once
conservation.sort.1=conservation.sort[c(1:100800),]
conservation.sort.2=conservation.sort[c(100801:201600),]
composites.func.panels.conservation.x(conservation.sort.1, fact = 'merge1',\
    summit = 'Motif', num=25)
composites.func.panels.conservation.x(conservation.sort.2, fact = 'merge2',\
    summit = 'Motif', num=25)
```

4.3.3 Rerun TOMTOM with a less stringent threshold

We rerun TOMTOM with threshold E-value of 10.0 to ensure that the candidate motif we found is an orphan using less stringent matching criteria. First create a folder called "orphans_tomtom", and copy-paste all the candidate orphan minimal MEME motif files there.

```
#bash ~/Desktop/Iterate_MCF7_hotspot_onestop/iteration/orphans_tomtom.sh
cd ~/Desktop/Iterate_MCF7_hotspot_onestop/iteration/orphans_tomtom

for file in *.txt
do
echo $file
filename=${file%.txt}
tomtom -verbosity 1 -o $filename.tomtom_output -min-overlap 5 -dist pearson\
    -evalue -thresh 10 -no-ssc $file ~/Desktop/Iterate_MCF7_hotspot/\
   motif_databases_tomtom/151207_combined_motif_db_JASPAR2016.txt
tomtomtxt=$filename.tomtom_output/tomtom.txt
motifid=$(awk 'FNR == 2 {print $2}' $tomtomtxt)
evalue=$(awk 'FNR == 2 {print $5}' $tomtomtxt)
#covert the scientific notation to float number
evaluefloat=$(printf "%f" "$evalue")
#convert float number to the closest interger below it
evaluefloor=${evaluefloat%.*}
echo evaluefloat:$evaluefloat
echo evaluefloor:$evaluefloor
echo motifid_$motifid
if [[ $motifid == "" ]]
then
echo $file >> orphansE10.txt
else
echo motifid_$motifid
echo $file >> nonorphansE10.txt
for tomtom in $filename.tomtom_output/*
do
echo $tomtom
file1=${tomtom%.*}
file2=${tomtom##*.}
mv $tomtom $filename.tomtom_output/${motifid}.${file2}
echo $motifid >> nonorphansE10.motif.txt
done
if  [[ $evaluefloor -ge 1 ]]
then
echo $file >> orphansE1.0.txt
fi
fi
done
```

Output file "orphansE10.txt" contains all the motifs deeming as orphans under a very stringent threshold of E=10. File "orphansE1.0.txt" contains all the motifs deeming as orphans with a less stringent threshold of E=1.

4.3.4 Post-hoc processing the information and identify candidate orphan motifs

Go through the conservation plots one by one and choose the orphan motifs with an obvious peak around the center of the motif. Double check whether the selected motif is a real orphan by looking into the TOMTOM results with a threshold of E=10. And then check the scrambled conservation plots, and make sure that no obvious peak exists around the center of the scrambled motif. Lastly, the motif with a DNase footprint or showing polarity in the footprint plots will be the top candidate orphan motif. Please refer to Figure 3 for an example. Note that this step was not automated at the time of the analysis.

5 Defining single nucleotide variates that may affect transcription factor binding in breast cancer

5.1 Find all regions that are in LD with the top GWAS SNPs

We identified all regions that are in linkage disequilibrium (LD) ($r^2 > 0.8$) with the top single nucleotide polymorphism (SNP) defined using the genome-wide association study (GWAS) catalog (HaploReg) focusing on breast cancer: `http://www.ebi.ac.uk/gwas/search?query=breast%20cancer`. Note that the output files (large_LD_regions_BRCA_GWAS_hg38.bed and SNVs_5column_BRCA_GWAS_hg38.bed) have coordinates in hg38. We need to lift over the coordinates to hg19.

```
#download software liftover from: http://hgdownload.soe.ucsc.edu/admin/exe/\
   macOSX.x86_64/liftOver
chmod +x liftOver
sudo mv liftOver /usr/local/bin/ucsc/
#download liftOver chain: http://hgdownload.cse.ucsc.edu/goldenPath/hg38/\
   liftOver/hg38ToHg19.over.chain.gz
#run liftOver on those two bed files
cd <dir>
liftOver -bedPlus=5 large_LD_regions_BRCA_GWAS_hg38.bed hg38ToHg19.over.\
   chain.gz -bedPlus=5 large_LD_regions_BRCA_GWAS_hg19.bed -bedPlus=5 \
   large_LD_regions_BRCA_GWAS_unmapped_hg38tohg19.bed
liftOver -bedPlus=5 SNVs_5column_BRCA_GWAS_hg38.bed hg38ToHg19.over.chain.\
   gz -bedPlus=5 SNVs_5column_BRCA_GWAS_hg19.bed -bedPlus=5 \
   SNVs_5column_BRCA_GWAS_unmapped_hg38tohg19.bed
#convert the bedtofasta file
fastaFromBed -fi <dir>/hg19.fa -bed large_LD_regions_BRCA_GWAS_hg19.bed -fo\
    large_LD_regions_BRCA_GWAS_hg19.fasta
fastaFromBed -fi <dir>/hg19.fa -bed SNVs_5column_BRCA_GWAS_hg19.bed -fo \
   SNVs_5column_BRCA_GWAS_hg19.fasta
```

5.2 Identification of all potential transcription factor binding sites underlying DNase/ATAC-defined regulatory region and overlapping with the GWAS LD regions

We scanned the overlapping regions of GWAS LD region and DNase/ATAC-defined regulatory region (peak region) found in all breast cancer cell lines for the PSWM using MAST with a threshold of

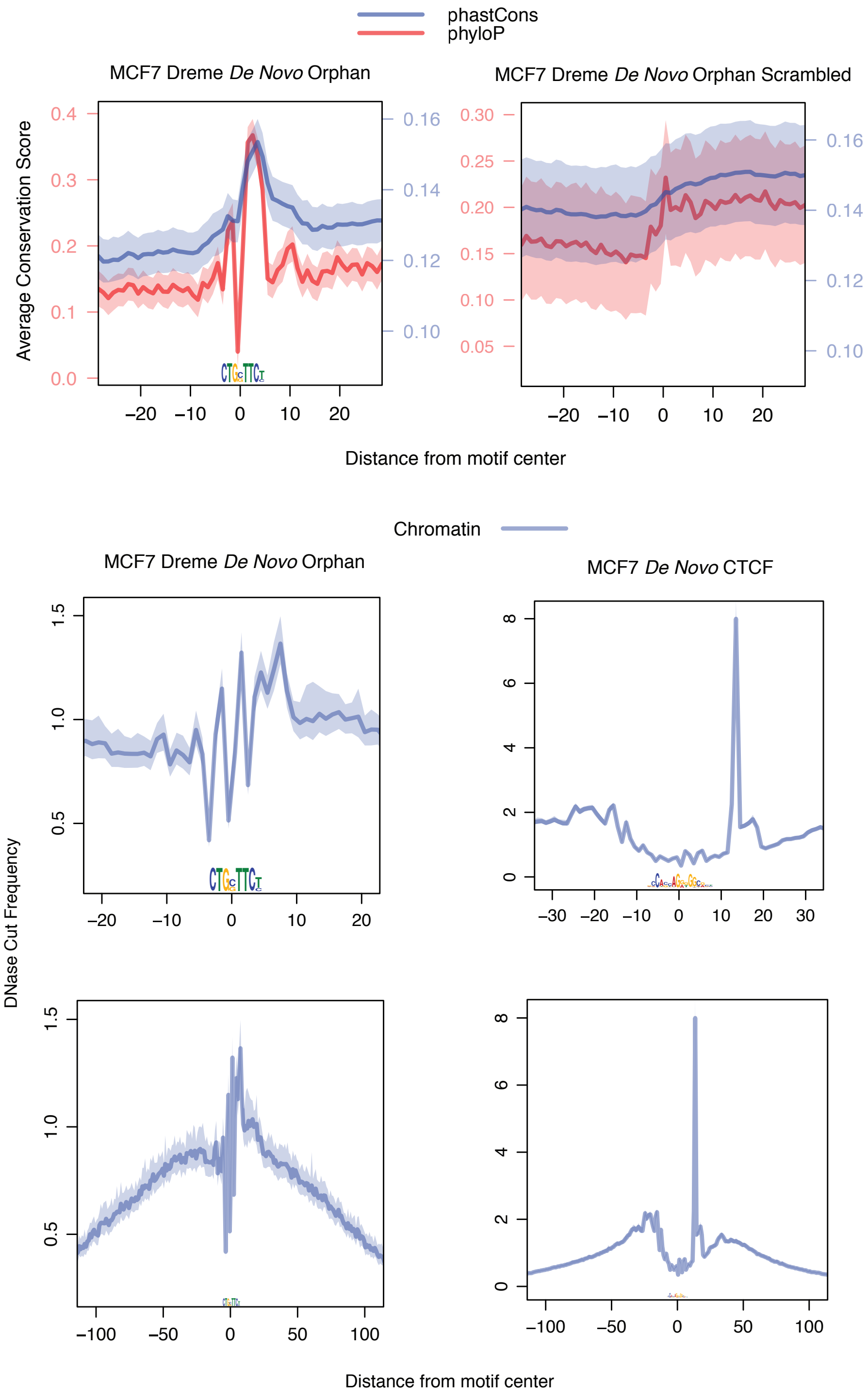

Figure 3: Orphan motif identified from MCF7

1E-4. For each PSWM, it is important to generate a PSWM that allows for complete degeneracy at each position in the PSWM. For example, for a PSWM with 10 positions, one would consider the original PSWM, and the 9 other PSWMs with a degeneracy at each position. We provide a Python script (MEME_full_degeneracy.py) here that is able to generate a degenerate MEME file concatenating all degenerate PSWM from one minimal MEME file in Appendix Q.

We automated the scanning process using the following bash script:

```
#run this script locally
#bash ~/BrCa_mast1e-4_ic0.5_automate_spreadsheet.sh

start=`date +%s`
cd ~
brca=<dir>
GWAS=$brca/GWAS
dir=$brca/GWAS/mast1e-4_ic0.5
script=<dir>

#overlap the merged snr bed file with the original broadpeak files of four \
    celllines
#generate celinehotspot_large_LD_regions_BRCA_GWAS_hg19_broadpeak.fasta

cat $brca/Iterate_HMEC_hotspot/Hotspots_hmec.sort.broadPeak | cut -f1-3 > \
    ~/Desktop/Iterate_HMEC_hotspot/Hotspots_hmec.sort_3col.broadPeak
HMEChotspot=$brca/Iterate_HMEC_hotspot_onestop/Hotspots_hmec.sort_3col.\
    broadPeak
MCF7hotspot=$brca/Iterate_MCF7_hotspot_onestop/Hotspots_Either_E2_condition\
    .merge.broadPeak
T47Dhotspot=$brca/Iterate_T47D_hotspot_onestop/t47d_hotspots.broadPeak
vHMEChotspot=$brca/Iterate_vHMEC_hotspot_onestop/vHMEC.bed
#liftover the MCF10A bed file from hg38 to hg19
liftOver $brca/Iterate_MCF10A_hotspot/MCF10a_merged.rmdup.fdr0.01.hot.bed \
    $brca/GWAS/hg38ToHg19.over.chain.gz  $brca/Iterate_MCF10A_hotspot/\
    MCF10a_merged.rmdup.fdr0.01.hg19.hot.bed $brca/Iterate_MCF10A_hotspot/\
    MCF10a_merged.rmdup.fdr0.01.liftoverunmapped.hot.bed
cat $brca/Iterate_MCF10A_hotspot_onestop/MCF10a_merged.rmdup.fdr0.01.hg19.\
    hot.bed | cut -f1-3 > $brca/Iterate_MCF10A_hotspot_onestop/MCF10a_merged\
    .rmdup.fdr0.01.hg19_3col.hot.bed
MCF10Ahotspot=$brca/Iterate_MCF10A_hotspot_onestop/MCF10a_merged.rmdup.fdr0\
    .01.hg19_3col.hot.bed

cat $HMEChotspot $MCF7hotspot $T47Dhotspot $vHMEChotspot $MCF10Ahotspot | \
    sort -k1,1 -k2,2n | mergeBed > $GWAS/celinehotspot_broadpeak.bed

fastaFromBed -fi <dir>/hg19.fa -bed $GWAS/celinehotspot_broadpeak.bed -fo \
    $GWAS/celinehotspot_broadpeak.fasta

allhotspot=$GWAS/celinehotspot_broadpeak.fasta

declare -a celinearray=("MCF7" "vHMEC" "HMEC" "T47D" "MCF10A")
declare -a celinelcarray=("mcf7" "vhmec" "hmec" "t47d" "mcf10a")

#run mast with a threshold of 1e-4
i=0
for celine in "${celinearray[@]}"
do
echo $celine
celine_lc=${celinelcarray[$i]}
echo ${celine_lc}
```

```
#make several folders for this cell line
mkdir $dir/${celine}_minimal_meme_files
mkdir $dir/${celine}_minimal_meme_degenerate

##from one-stop iteration
#first move all the orphan motifs into a seperate folder
mkdir $brca/Iterate_${celine}_hotspot_onestop/iteration/withmotif/\
    minimal_meme_orphan
mv $brca/Iterate_${celine}_hotspot_onestop/iteration/withmotif/*_.txt $brca\
    /Iterate_${celine}_hotspot_onestop/iteration/withmotif/\
    minimal_meme_orphan
#then copy the rest minimal meme files into a sperate folder created before
cp $brca/Iterate_${celine}_hotspot_onestop/iteration/withmotif/*.txt $dir/$\
    {celine}_minimal_meme_files

##from memechip_meme
#first move all the orphan motifs into a seperate folder
mkdir $brca/Iterate_${celine}_hotspot_onestop/iteration/memechip/\
    memechip_meme_individual/motif_orphans_meme
mv $brca/Iterate_${celine}_hotspot_onestop/iteration/memechip/\
    memechip_meme_individual/motif_orphan*_meme.txt $brca/Iterate_${celine}\
    _hotspot_onestop/iteration/memechip/memechip_meme_individual/\
    motif_orphans_meme
#then copy the rest minimal meme files into a seperate folder created \
    before
cp $brca/Iterate_${celine}_hotspot_onestop/iteration/memechip/\
    memechip_meme_individual/motif_*_meme.txt $dir/${celine}\
    _minimal_meme_files

##from memechip_dreme
mkdir $brca/Iterate_${celine}_hotspot_onestop/iteration/memechip/\
    memechip_dreme_individual/motif_orphans_dreme
mv $brca/Iterate_${celine}_hotspot_onestop/iteration/memechip/\
    memechip_dreme_individual/motif_orphan*_dreme.txt $brca/Iterate_${celine\
    }_hotspot_onestop/iteration/memechip/memechip_dreme_individual/\
    motif_orphans_dreme
#then copy the rest minimal meme files into a seperate folder created \
    before
cp $brca/Iterate_${celine}_hotspot_onestop/iteration/memechip/\
    memechip_dreme_individual/motif_*_dreme.txt $dir/${celine}\
    _minimal_meme_files

##make the degenerate files
cd $dir/${celine}_minimal_meme_files
for file in *
do
before=${file%.txt}
python $script/MEME_full_degeneracy.py -i $file -o $before
done

cd ~

#move all the degenerate file to the degenerate folder
mv $dir/${celine}_minimal_meme_files/*degenerate.txt $dir/${celine}\
    _minimal_meme_degenerate/

cd $dir/${celine}_minimal_meme_degenerate/
for file in *degenerate.txt
do
```

```
before=${file%.txt}
mkdir ${before}_1mtE-4.mast_output
filename=${before##*/}
mast $file $allhotspot -hit_list > ${before}_1mtE-4.mast_output/${filename\
    }.txt
python $script/MAST_bed_conversion.py -i  ${before}_1mtE-4.mast_output/${\
    filename}.txt
done
(( i = i +1 ))
done
```

5.3 Determination of the polymorphism of TFBS

We obtained the information content of each single nucleotide and keep the SNV with information content higher than 0.5. An R script (tf_ic_track.R) was developed by André Martins for this purpose, which is provided in Appendix R.

We determined if any of those potential TF binding site overlaps with a SNP in LD block with a top GWAS SNP. The bash script is provided below.

```
#run this script locally
#bash BrCa_mast1e-4_ic0.5_automate_spreadsheet.sh
#change: mast with the mergedbed file of hotspot fasata file and LD fasta \
    file

cd ~
brca=<dir>
GWAS=$brca/GWAS
dir=$brca/GWAS/mast1e-4_ic0.5
script=<dir>

declare -a celinearray=("MCF7" "vHMEC" "HMEC" "T47D" "MCF10A")
declare -a celinelcarray=("mcf7" "vhmec" "hmec" "t47d" "mcf10a")

cd ~
i=0
for celine in "${celinearray[@]}"
do
echo $celine
celine_lc=${celinelcarray[$i]}
echo ${celine_lc}

SNR=$dir/${celine}_SNR
mkdir $SNR
cp $dir/${celine}_minimal_meme_files/* $SNR
cp $dir/${celine}_minimal_meme_degenerate/*1mtE-4.mast_output/*bed $SNR

for file in $SNR/*.txt
do
filenametxt=${file##*/}
filename=${filenametxt%.txt}
echo generate snr bed file:
#note that the threshold cutoff for IC=0.5
Rscript $script/tf_ic_track.R $file $SNR/${filename}_degenerate.bed --\
    thresh=0.5 -o $SNR/${filename}_snr.bed
echo $SNR/${filename}_snr.bed
done
```

```
sort -k1,1 -k2,2n $SNR/*_snr.bed | mergeBed > $dir/${celine}_snr.bed

(( i = i +1 ))
done

sort -k1,1 -k2,2n $dir/*snr.bed | mergeBed > $dir/snr_merged.bed

#changed here
intersectBed  -wb -a $dir/snr_merged.bed -b $GWAS/celinehotspot_broadpeak.\
    bed > $dir/snr_merged_hotspot.bed

intersectBed  -wa -a $dir/snr_merged_hotspot.bed -b $GWAS/BRCA_GWAS/\
    large_LD_regions_BRCA_GWAS_hg19.bed > $dir/snr_merged_hotspot_ld.bed

#overalp the hotspot file with the SNVs bed file
intersectBed -wb -a $dir/snr_merged_hotspot_ld.bed -b $GWAS/BRCA_GWAS/\
    SNVs_5column_BRCA_GWAS_hg19.bed > $dir/snr_merged_hotspot_ld_snvs.bed

sort -u $dir/snr_merged_hotspot_ld_snvs.bed | grep -v -e '^$'> $dir/\
    snr_merged_hotspot_ld_snvs_unique.bed

cp $dir/snr_merged_hotspot_ld_snvs_unique.bed $dir/\
    snr_merged_hotspot_snvs_final_hg19.bed

#liftover the bed file from hg19 back to hg38
liftOver -bedPlus=3 $dir/snr_merged_hotspot_snvs_final_hg19.bed <dir>/\
    hg19ToHg38.over.chain.gz -bedPlus=3 $dir/\
    snr_merged_hotspot_snvs_final_hg38.bed -bedPlus=3 $dir/\
    snr_merged_hotspot_snvs_final_unmapped_hg19tohg38.bed

#keep the first 13 columns of the SNVs_BRCA_GWAS.bed
cat $GWAS/BRCA_GWAS/SNVs_BRCA_GWAS.bed | cut -f1-13,34 > $GWAS/BRCA_GWAS/\
    SNVs_BRCA_GWAS_14_cols_pre.bed
#remove the duplicates
sort -u $GWAS/BRCA_GWAS/SNVs_BRCA_GWAS_14_cols_pre.bed > $GWAS/BRCA_GWAS/\
    SNVs_BRCA_GWAS_14_cols.bed
#for this 14_cols spreadsheet, the last col is the top SNP

#automatically generate the spreadsheet
cat $dir/snr_merged_hotspot_snvs_final_hg38.bed | cut -f1-3,7- > $dir/\
    snr_merged_hotspot_snvs_final_hg38_auto.bed
cat $dir/snr_merged_hotspot_snvs_final_hg38_auto.bed | while read line
do
topsnp=$(cut -f8 <<< "$line")
echo topsnp:$topsnp

ldsnp=$(cut -f7 <<< "$line")
echo ldsnp:$ldsnp
#The -v option can be used to pass shell variables to awk command.
r2=$(awk -v t=$topsnp '$14 == t' $GWAS/BRCA_GWAS/SNVs_BRCA_GWAS_14_cols.bed\
     | awk -v s=$ldsnp '$7 == s' | cut -f4)
echo r2:$r2
echo $r2 >> $dir/r2.txt
AFR=$(awk -v t=$topsnp '$14 == t' $GWAS/BRCA_GWAS/SNVs_BRCA_GWAS_14_cols.\
    bed | awk -v s=$ldsnp '$7 == s' | cut -f10)
echo AFR:$AFR
echo $AFR >> $dir/AFR.txt
AMR=$(awk -v t=$topsnp '$14 == t' $GWAS/BRCA_GWAS/SNVs_BRCA_GWAS_14_cols.\
    bed | awk -v s=$ldsnp '$7 == s' | cut -f11)
```

```
echo AMR:$AMR
echo $AMR >> $dir/AMR.txt
ASN=$(awk -v t=$topsnp '$14 == t' $GWAS/BRCA_GWAS/SNVs_BRCA_GWAS_14_cols.\
    bed | awk -v s=$ldsnp '$7 == s' | cut -f12)
echo ASN:$ASN
echo $ASN >> $dir/ASN.txt
EUR=$(awk -v t=$topsnp '$14 == t' $GWAS/BRCA_GWAS/SNVs_BRCA_GWAS_14_cols.\
    bed | awk -v s=$ldsnp '$7 == s' | cut -f13)
echo EUR:$EUR
echo $EUR >> $dir/EUR.txt

cor=$(cut -f5 <<< "$line")
echo cor:$cor

chr=$(cut -f1 <<< "$line")
echo chr:$chr

#make the following process stop at the first match, there might be \
    downside with it
risk_allele=$(grep -o $topsnp-[A-Z] $GWAS/BRCA_GWAS/BRCA_GWAS.txt.tsv | \
    head -1 | cut -d "-" -f2)
echo risk_allele:$risk_allele
echo $risk_allele >> $dir/risk_allele.txt
#first find the row corresponding to the topsnp, and then find the column \
    number corresponding to the risk_allele
risk_col=$(grep $topsnp $GWAS/BRCA_GWAS/SNVs_BRCA_GWAS_3col.bed | awk -v \
    val=$risk_allele '{for (i=1;i<=NF;i++) if ($i==val) {print i} }')
echo risk_col:$risk_col
#v2 add-on starts here
if  [  "$risk_col" == ""  -a  "$risk_allele" != ""  ]
then
echo risk_allele is on the reverse strand
if [ "$risk_allele" == "A" ]
then
risk_allele=T
elif [ "$risk_allele" == "T" ]
then
risk_allele=A
elif [ "$risk_allele" == "G" ]
then
risk_allele=C
elif [ "$risk_allele" == "C" ]
then
risk_allele=G
fi
fi
risk_col=$(grep $topsnp $GWAS/BRCA_GWAS/SNVs_BRCA_GWAS_3col.bed | awk -v \
    val=$risk_allele '{for (i=1;i<=NF;i++) if ($i==val) {print i} }')
echo risk_col:$risk_col
alt_col=$((5-risk_col))
echo alt_col:$alt_col
ld_allele=$(grep $ldsnp $GWAS/BRCA_GWAS/SNVs_BRCA_GWAS_3col.bed | cut -\
    f$risk_col)
echo ld_allele:$ld_allele
echo $ld_allele >> $dir/ld_allele.txt
alt_allele=$(grep $ldsnp $GWAS/BRCA_GWAS/SNVs_BRCA_GWAS_3col.bed | cut -\
    f$alt_col)
echo alt_allele:$alt_allele
echo $alt_allele >> $dir/alt_allele.txt
```

```
/bin/echo '' >> $dir/enhance_binding.txt
/bin/echo '' >> $dir/compromise_binding.txt
/bin/echo '' >> $dir/cannot_predict_binding.txt
#continue ld_allele is identified, that is the not an empty string
if [ "$ld_allele" != "" ]
then
#keep col6 in the following, which contain the plus or minus strand \
    information
#I need to first grep on the "chr" information, and then grep on the "cor" \
    information
#add -w for grep, so that I will get an exact match
grep -r -w $cor $dir/*_SNR/*_snr.bed | cut -f1,2,4,5,6 |
while read line
do
file=$(cut -f1 <<< "$line")
starpos=$(cut -f2 <<< "$line")
allele=$(cut -f3 <<< "$line")
ic=$(cut -f4 <<< "$line")
strand=$(cut -f5 <<< "$line")
#note, you need to change here if using a different ic threshold
after=${file#*_ic0.5/}
before=${after%_snr.bed*}
motif=${before%_num*}
echo $motif
outputchr=${file##*:}
echo outputchr:$outputchr

if [ "$outputchr" == "$chr" ]
then
if [ "$starpos" == "$cor" -a "$allele" == "$ld_allele" ]
then
echo enhance binidng: $motif
echo $file
/bin/echo $motif\($allele,$ic,$strand\),'\c' >> $dir/enhance_binding.txt
elif [ "$starpos" == "$cor" -a "$allele" == "$alt_allele" ]
then
echo comprise binding: $motif
echo $file
/bin/echo $motif\($allele,$ic,$strand\),'\c' >> $dir/compromise_binding.txt
elif [ "$starpos" == "$cor" -a "$allele" != "$ld_allele" -a "$allele" != "\
    $alt_allele" ]
then
echo cannot predict: $motif
echo $file
/bin/echo $motif\($allele,$ic,$strand\),'\c' >> $dir/cannot_predict_binding\
    .txt
fi
fi
done
fi
done

paste $dir/enhance_binding.txt $dir/compromise_binding.txt $dir/\
    cannot_predict_binding.txt | tail -n +2 > $dir/merge.txt
paste $dir/snr_merged_hotspot_snvs_final_hg38_auto.bed $dir/risk_allele.txt\
     $dir/ld_allele.txt $dir/alt_allele.txt $dir/r2.txt $dir/AFR.txt $dir/\
    AMR.txt $dir/ASN.txt $dir/EUR.txt $dir/merge.txt > $dir/mast1e-4_ic0.5\
    _automate_spreadsheet_pre.txt
#add header to this file
```

```
echo $'CHR\tHG38 START COR\tHG38 END COR\tCHR\t HG19 START COR\tHG19 END \
    COR\tLD SNP\tTOP GWAS SNP\tRISK ALLELE\tLD ALLELE\tALT ALLELE\tLD (R^2)\\
    tALT Allele AFR\tALT Allele AMR\tALT Allele ASN\tALT Allele EUR\tENHANCE\
     TF BINDING\tCOMPROMISE TF BINDING\tCANNOT PREDICT TF BINDING' | cat - \
    $dir/mast1e-4_ic0.5_automate_spreadsheet_pre.txt > $dir/mast1e-4_ic0.5\
    _automate_spreadsheet.txt
rm $dir/enhance_binding.txt $dir/compromise_binding.txt $dir/\
    cannot_predict_binding.txt $dir/risk_allele.txt $dir/ld_allele.txt $dir/\
    alt_allele.txt $dir/r2.txt $dir/AFR.txt $dir/AMR.txt $dir/ASN.txt $dir/\
    EUR.txt $dir/merge.txt $dir/mast1e-4_ic0.5_automate_spreadsheet_pre.txt
```

5.4 Linking candidate causal variants to genes – eQTL analysis

Expression quantitative trait locus (eQTL) refers to a genetic locus where the genotype of a variant is significantly associated with gene expression levels of one or more genes. The causal variant may be any type of DNA variants, including SNPs, indels or copy number variants. Our study focused on *cis*-eQTL, which is a genetic variant that influences the expression levels of a proximal gene (within 1MB upstream and downstream of the genetic variant) on the same chromosome in an allele-specific manner (Ardlie *et al.*, 2015).

5.4.1 Retrieving TCGA gene expression data

Gene expression was quantified from TCGA curated data for breast cancer, we obtained the data from Cancer Genome Atlas. The Cancer Genomics Browser currently hosts 575 public datasets from genome-wide analyses of over 227,000 samples, including datasets from TCGA, CCLE, Connectivity Map and TARGET (Goldman *et al.*, 2014). Search for "Breast Cancer" on the Cancer Genomics Browser (`https://genome-cancer.ucsc.edu/proj/site/hgHeatmap/?datasetSearch=breast+cancer`). Download following data set: TCGA_BRCA_exp_HiSeqV2_PANCAN-2015-02-24.tgz.

5.4.2 TCGA patients genotype data

Genotype for the TCGA patient were obtained from TCGA SNP6 arrays that were hybridized with DNA extracted from blood. We processed the corresponding TCGA birdseed files (`http://www.ncbi.nlm.nih.gov/projects/gap/cgi-bin/study.cgi?study_id=phs000178.v9.p8`) and imputed using the Michigan Imputation Server: 1000G Phase v3 Shapeit2 Refernece Panel, SHAPEIT Phasing; Mixed Population; Quality Control and Imputation Mode. The bash script is provided in Appendix S.

We obtained 22 VCF files for 22 chromosomes. Variant Call Format (VCF) file is a text file format (most likely stored in a compressed manner). It contains meta-information lines, a header line, and then data lines each containing information about a position in the genome (`http://www.1000genomes.org/wiki/Analysis/Variant%20Call%20Format/vcf-variant-call-format-version-40`).

Variant sites were filtered for a minor allele frequency (MAF) of at least 5%. Assessed sites satisfied threshold of 1E-6 for Hardy-Weinberg Equilibrium (HWE). Tested variants were within 1 Mb of the our top candidate SNPs that are within DNase-defined regulatory regions of breast-derived cell lines or tissues and also in LD region with the top GWAS SNPs. This process utilized VCFtools (version 0.1.14): `http://vcftools.sourceforge.net/` (Danecek *et al.*, 2011).

```
#!/usr/local/Cellar/bash/4.3.42/bin/bash
#run the bash script locally
#bash <dir>/tcga_patients_genotype_data.sh
```

```
dir=<dir>
imputation=<dir>/imputation
automate=<dir>/mast1e-4_ic0.5_automate_spreadsheet.txt

cd $dir

#read in the chromosome, cordinates, and snp name information from the file
readarray -t chrarray < <(cut -f1 $automate | tail -n+2)
#those cordinates are in hg19
readarray -t corarray < <(cut -f5 $automate | tail -n+2)
readarray -t snparray < <(cut -f7 $automate | tail -n+2)

i=0
for chr in "${chrarray[@]}"
do
    echo $chr
 #newly added command
    chrnum=${chr#chr}
    echo $chrnum
    cor=${corarray[$i]}
    echo $cor
    snp=${snparray[$i]}
    echo $snp
    mb=1000000

firstcor=$(($cor-$mb))
echo firstcor:$firstcor
lastcor=$(($cor+$mb))
echo lastcor:$lastcor

vcfgz=$imputation/imputation_results/chr_$chrnum/chr$chrnum.dose.vcf.gz
vcftools --gzvcf $vcfgz --out $dir/chr${chrnum}_${snp}_filter --chr $chrnum\
     --from-bp $firstcor --to-bp $lastcor --maf 0.05 --hwe 1e-6 --remove-\
    filtered-geno-all --recode

(( i = i +1 ))
done
```

5.4.3 Retrieving TCGA clinical information

TCGA clinical data include clinical information about the participant such as age, gender, and information about the how participant samples (biospecimens) were processed by the TCGA Biospecimen Core Resource Center (BCR): `https://wiki.nci.nih.gov/display/TCGA/Clinical+Data+Overview`. We retrieved TCGA clinical data for breast cancer patient with RTCGA package from Bioconductor using `R`: `https://www.bioconductor.org/packages/release/bioc/manuals/RTCGA/man/RTCGA.pdf`.

Detailed `R` code is included "create_covariant_file" section of Appendix T.

In short, we selected 33 categories of clinical information as the covariates for eQTL analysis such as RNA concentration, quantity, rinvalue, rna.ratio_28s_18s, patient.ethnicity, and patient.gender. We performed Principal Component Analysis (PCA) on the quantitative variables and took the first three principal components, we retained all the other qualitative variables. Note we retrieved the patient population structure information with PLINK (v1.07) from the merged TCGA vcf files obtained in 5.4.2: `http://pngu.mgh.harvard.edu/~purcell/plink/strat.shtml`. The bash code obtaining population structure data is provided below:

```
#convert vcf to PLINK
```

```
vcf-concat $(ls -1 TCGA_BrCA_chr*merged.vcf.gz | perl -pe 's/\n/ /g') | \
    bgzip -c > TCGA_BrCa_all_chr_merged.vcf.gz
plink --vcf TCGA_BrCa_all_chr_merged.vcf.gz --make-bed --out \
    TCGA_BrCa_all_chr_merged
plink --bfile TCGA_BrCa_all_chr_merged --map3 --no-fid --no-parents --no-\
    sex --no-pheno --compound-genotypes  --mind 0.03 --geno 0.05 --maf 0.01 \
    --hwe 1e-6 --make-bed --out TCGA_BrCa_all_chr_merged_filtered

plink --file TCGA_BrCa_all_chr_merged_filtered --cluster --K 4 -out \
    TCGA_BrCa_all_chr_merged_filtered
```

5.4.4 eQTL analysis using fastQTL

Discovery of eQTLs in our candidate SNPs were asspcoated with genes within plus/minus 1 Mb using a mixed linear regression model in fastQTL (v2.0): `http://fastqtl.sourceforge.net/` (Ongen *et al.*, 2015). We also subset those genes by focusing on the genes measured by TCGA curated data (please refer to section 5.4.1). We provide the `R` script to perform eQTL analysis in appendix T. Several embedded bash scripts for this `R` script are provided in appendix U (fastQTL_geno_bgzip.sh) and V (fastQTL.sh).

Note that we obtained the start and end coordinates of genes within plus/minus 1 Mb of our candidate SNPs from UCSC genome browser: `https://genome.ucsc.edu/training/vids/index.html#vid01`. First, generate a three-column bed file ("snps_defined_region_for_ucsc_genome_browser.txt") specifying the chromosome number, start position, and the end position covering 1Mb region upstream and downstream of each candidate SNP.

```
#!/usr/local/Cellar/bash/4.3.42/bin/bash
#run the bash script locally
#bash ~/snp_location_ucsc_browser.sh

dir=<dir>
automate=<dir>/mast1e-4_ic0.5_automate_spreadsheet.txt

cd $dir

#read in the chromosome, cordinates, and snp name information from the file
readarray -t chrarray < <(cut -f1 $automate | tail -n+2)
#those cordinates are in hg19
readarray -t corarray < <(cut -f5 $automate | tail -n+2)
readarray -t snparray < <(cut -f7 $automate | tail -n+2)

i=0
for chr in "${chrarray[@]}"
do
echo chr:$chr
#newly added command
chrnum=${chr#chr}
echo $chrnum
snp=${snparray[$i]}
echo snp:$snp
cor=${corarray[$i]}
echo cor:$cor
mb=1000000

firstcor=$(($cor-$mb))
echo firstcor:$firstcor
lastcor=$(($cor+$mb))
```

```
echo lastcor:$lastcor

if [[ "$firstcor" -lt "0" ]]
then
echo firstcor is negative:$firstcor
firstcor=1
fi

echo position: $chr $firstcor $lastcor
echo $chr $firstcor $lastcor >> snps_defined_region_for_ucsc_genome_browser\
    .txt

(( i = i +1 ))
done
```

Then go to the "Table Browser (https://genome.ucsc.edu/cgi-bin/hgTables)" section of UCSC genome browser, and obtain "1mb_snps.txt" containing the all the SNPs within 1MB of identified candidate causal BrCa SNPs. Select the following parameters:
clade - Mammal; genome - Human; assembly - Feb. 2009 (GRCh37/hg19); group - Variation; track - Common SNPs(147); table - snp147Common; region - defined regions (upload and submit "snps_defined_region_for_ucsc_genome_browser.txt"); outputformat - selected fields from primary and related tables; output file - "1mb_snps.txt"; file type returned - plain text
Click on "get output" and tick the following output parameters: chrom, chromStart, chromEnd, name, alleles, and alleleFreqs.

We visualized eQTL results using LocusZoom (Version 1.1) (Pruim *et al.*, 2010). Directly upload the LocusZoom-formated text file (e.g., chr19_rs4802200_ZNF404_locuszoom.txt) from the eQTL analysis R script (Appendix T) to http://locuszoom.sph.umich.edu/locuszoom/genform.php?type=yourdata, specify SNP Reference Name (e.g., rs4802200) and flanking size (e.g., 100 Kb), and click "Plot Data".

We also corroborated the eQTL results by referring to GTEx (Consortium *et al.*, 2015), the version of GTEx used at the time of analysis was GTEX Analysis v6 (dbGAP Accession phs000424.v6.p1). The dataset is available at http://www.gtexportal.org/home/datasets. Note that it is different from GTEx Analysis V6p, which is a patch for the V6 release, and provides new gene-level expression quantifications and eQTL results based on an improved gene-level annotation derived from GENCODE v19.

5.5 Allele-specific TF binding

We analyzed ENCODE CTCF ChIP-seq data for allele-specific preference for rs4414128 and rs8103622 because CTCF has been profiled by ChIP-seq more than any other sequence-specific binding protein and these candidate SNPs are the two highest intensity CTCF binding sites across cell types. 37 cell types (and replicated) that are heterozygous at rs8103622 and normal karyotype were investigated. 44 cell types (and replicated) that are heterozygous at rs4414128 and normal karyotype were investigated.

The following ENCODE CTCF ChIP-seq data can be downloaded from: http://hgdownload.cse.ucsc.edu/goldenpath/hg19/encodeDCC/wgEncodeUwTfbs/.
wgEncodeUwTfbsAg04449CtcfStdAlnRep1.bam.bai
wgEncodeUwTfbsAg04449CtcfStdAlnRep2.bam
wgEncodeUwTfbsAg04449CtcfStdAlnRep2.bam.bai
wgEncodeUwTfbsAg04450CtcfStdAlnRep1.bam
wgEncodeUwTfbsAg04450CtcfStdAlnRep1.bam.bai
wgEncodeUwTfbsAg04450CtcfStdAlnRep2.bam
wgEncodeUwTfbsAg04450CtcfStdAlnRep2.bam.bai

wgEncodeUwTfbsAg09309CtcfStdAlnRep1.bam
wgEncodeUwTfbsAg09309CtcfStdAlnRep1.bam.bai
wgEncodeUwTfbsAg09309CtcfStdAlnRep2.bam
wgEncodeUwTfbsAg09309CtcfStdAlnRep2.bam.bai
wgEncodeUwTfbsAg09319CtcfStdAlnRep1.bam
wgEncodeUwTfbsAg09319CtcfStdAlnRep1.bam.bai
wgEncodeUwTfbsAg09319CtcfStdAlnRep2.bam
wgEncodeUwTfbsAg09319CtcfStdAlnRep2.bam.bai
wgEncodeUwTfbsAg10803CtcfStdAlnRep1.bam
wgEncodeUwTfbsAg10803CtcfStdAlnRep1.bam.bai
wgEncodeUwTfbsAg10803CtcfStdAlnRep2.bam
wgEncodeUwTfbsAg10803CtcfStdAlnRep2.bam.bai
wgEncodeUwTfbsAoafCtcfStdAlnRep1.bam
wgEncodeUwTfbsAoafCtcfStdAlnRep1.bam.bai
wgEncodeUwTfbsAoafCtcfStdAlnRep2.bam
wgEncodeUwTfbsAoafCtcfStdAlnRep2.bam.bai
wgEncodeUwTfbsBjCtcfStdAlnRep1.bam
wgEncodeUwTfbsBjCtcfStdAlnRep1.bam.bai
wgEncodeUwTfbsBjCtcfStdAlnRep2.bam
wgEncodeUwTfbsBjCtcfStdAlnRep2.bam.bai
wgEncodeUwTfbsGm06990CtcfStdAlnRep1.bam
wgEncodeUwTfbsGm06990CtcfStdAlnRep1.bam.bai
wgEncodeUwTfbsGm06990CtcfStdAlnRep2.bam
wgEncodeUwTfbsGm06990CtcfStdAlnRep2.bam.bai
wgEncodeUwTfbsGm12801CtcfStdAlnRep1.bam
wgEncodeUwTfbsGm12801CtcfStdAlnRep1.bam.bai
wgEncodeUwTfbsGm12864CtcfStdAlnRep1.bam
wgEncodeUwTfbsGm12864CtcfStdAlnRep1.bam.bai
wgEncodeUwTfbsGm12864CtcfStdAlnRep2.bam
wgEncodeUwTfbsGm12864CtcfStdAlnRep2.bam.bai
wgEncodeUwTfbsGm12864CtcfStdAlnRep3.bam
wgEncodeUwTfbsGm12864CtcfStdAlnRep3.bam.bai
wgEncodeUwTfbsGm12865CtcfStdAlnRep1.bam
wgEncodeUwTfbsGm12865CtcfStdAlnRep1.bam.bai
wgEncodeUwTfbsGm12865CtcfStdAlnRep2.bam
wgEncodeUwTfbsGm12865CtcfStdAlnRep2.bam.bai
wgEncodeUwTfbsGm12865CtcfStdAlnRep3.bam
wgEncodeUwTfbsGm12865CtcfStdAlnRep3.bam.bai
wgEncodeUwTfbsGm12866CtcfStdAlnRep1.bam
wgEncodeUwTfbsGm12866CtcfStdAlnRep1.bam.bai
wgEncodeUwTfbsGm12866CtcfStdAlnRep2.bam
wgEncodeUwTfbsGm12866CtcfStdAlnRep2.bam.bai
wgEncodeUwTfbsGm12867CtcfStdAlnRep1.bam
wgEncodeUwTfbsGm12867CtcfStdAlnRep1.bam.bai
wgEncodeUwTfbsGm12867CtcfStdAlnRep2.bam
wgEncodeUwTfbsGm12867CtcfStdAlnRep2.bam.bai
wgEncodeUwTfbsGm12868CtcfStdAlnRep1.bam
wgEncodeUwTfbsGm12868CtcfStdAlnRep1.bam.bai
wgEncodeUwTfbsGm12868CtcfStdAlnRep2.bam
wgEncodeUwTfbsGm12868CtcfStdAlnRep2.bam.bai
wgEncodeUwTfbsGm12869CtcfStdAlnRep1.bam
wgEncodeUwTfbsGm12869CtcfStdAlnRep1.bam.bai
wgEncodeUwTfbsGm12869CtcfStdAlnRep2.bam
wgEncodeUwTfbsGm12869CtcfStdAlnRep2.bam.bai

wgEncodeUwTfbsGm12870CtcfStdAlnRep1.bam
wgEncodeUwTfbsGm12870CtcfStdAlnRep1.bam.bai
wgEncodeUwTfbsGm12870CtcfStdAlnRep2.bam
wgEncodeUwTfbsGm12870CtcfStdAlnRep2.bam.bai
wgEncodeUwTfbsGm12871CtcfStdAlnRep1.bam
wgEncodeUwTfbsGm12871CtcfStdAlnRep1.bam.bai
wgEncodeUwTfbsGm12871CtcfStdAlnRep2.bam
wgEncodeUwTfbsGm12871CtcfStdAlnRep2.bam.bai
wgEncodeUwTfbsGm12872CtcfStdAlnRep1.bam
wgEncodeUwTfbsGm12872CtcfStdAlnRep1.bam.bai
wgEncodeUwTfbsGm12872CtcfStdAlnRep2.bam
wgEncodeUwTfbsGm12872CtcfStdAlnRep2.bam.bai
wgEncodeUwTfbsGm12872CtcfStdAlnRep3.bam
wgEncodeUwTfbsGm12872CtcfStdAlnRep3.bam.bai
wgEncodeUwTfbsGm12873CtcfStdAlnRep1.bam
wgEncodeUwTfbsGm12873CtcfStdAlnRep1.bam.bai
wgEncodeUwTfbsGm12873CtcfStdAlnRep2.bam
wgEncodeUwTfbsGm12873CtcfStdAlnRep2.bam.bai
wgEncodeUwTfbsGm12873CtcfStdAlnRep3.bam
wgEncodeUwTfbsGm12873CtcfStdAlnRep3.bam.bai
wgEncodeUwTfbsGm12874CtcfStdAlnRep1.bam
wgEncodeUwTfbsGm12874CtcfStdAlnRep1.bam.bai
wgEncodeUwTfbsGm12874CtcfStdAlnRep2.bam
wgEncodeUwTfbsGm12874CtcfStdAlnRep2.bam.bai
wgEncodeUwTfbsGm12875CtcfStdAlnRep1.bam
wgEncodeUwTfbsGm12875CtcfStdAlnRep1.bam.bai
wgEncodeUwTfbsGm12875CtcfStdAlnRep2.bam
wgEncodeUwTfbsGm12875CtcfStdAlnRep2.bam.bai
wgEncodeUwTfbsGm12878CtcfStdAlnRep1.bam
wgEncodeUwTfbsGm12878CtcfStdAlnRep1.bam.bai
wgEncodeUwTfbsGm12878CtcfStdAlnRep2.bam
wgEncodeUwTfbsGm12878CtcfStdAlnRep2.bam.bai
wgEncodeUwTfbsHacCtcfStdAlnRep1.bam
wgEncodeUwTfbsHacCtcfStdAlnRep1.bam.bai
wgEncodeUwTfbsHacCtcfStdAlnRep2.bam
wgEncodeUwTfbsHacCtcfStdAlnRep2.bam.bai
wgEncodeUwTfbsHaspCtcfStdAlnRep1.bam
wgEncodeUwTfbsHaspCtcfStdAlnRep1.bam.bai
wgEncodeUwTfbsHaspCtcfStdAlnRep2.bam
wgEncodeUwTfbsHaspCtcfStdAlnRep2.bam.bai
wgEncodeUwTfbsHbmecCtcfStdAlnRep1.bam
wgEncodeUwTfbsHbmecCtcfStdAlnRep1.bam.bai
wgEncodeUwTfbsHbmecCtcfStdAlnRep2.bam
wgEncodeUwTfbsHbmecCtcfStdAlnRep2.bam.bai
wgEncodeUwTfbsHcfaaCtcfStdAlnRep1.bam
wgEncodeUwTfbsHcfaaCtcfStdAlnRep1.bam.bai
wgEncodeUwTfbsHcmCtcfStdAlnRep1.bam
wgEncodeUwTfbsHcmCtcfStdAlnRep1.bam.bai
wgEncodeUwTfbsHcmCtcfStdAlnRep2.bam
wgEncodeUwTfbsHcmCtcfStdAlnRep2.bam.bai
wgEncodeUwTfbsHcpeCtcfStdAlnRep1.bam
wgEncodeUwTfbsHcpeCtcfStdAlnRep1.bam.bai
wgEncodeUwTfbsHcpeCtcfStdAlnRep2.bam
wgEncodeUwTfbsHcpeCtcfStdAlnRep2.bam.bai

wgEncodeUwTfbsHeeCtcfStdAlnRep1.bam
wgEncodeUwTfbsHeeCtcfStdAlnRep1.bam.bai
wgEncodeUwTfbsHeeCtcfStdAlnRep2.bam
wgEncodeUwTfbsHeeCtcfStdAlnRep2.bam.bai
wgEncodeUwTfbsHffCtcfStdAlnRep1.bam
wgEncodeUwTfbsHffCtcfStdAlnRep1.bam.bai
wgEncodeUwTfbsHffmycCtcfStdAlnRep1.bam
wgEncodeUwTfbsHffmycCtcfStdAlnRep1.bam.bai
wgEncodeUwTfbsHffmycCtcfStdAlnRep2.bam
wgEncodeUwTfbsHffmycCtcfStdAlnRep2.bam.bai
wgEncodeUwTfbsHmecCtcfStdAlnRep1.bam
wgEncodeUwTfbsHmecCtcfStdAlnRep1.bam.bai
wgEncodeUwTfbsHmecCtcfStdAlnRep2.bam
wgEncodeUwTfbsHmecCtcfStdAlnRep2.bam.bai
wgEncodeUwTfbsHmfCtcfStdAlnRep1.bam
wgEncodeUwTfbsHmfCtcfStdAlnRep1.bam.bai
wgEncodeUwTfbsHmfCtcfStdAlnRep2.bam
wgEncodeUwTfbsHmfCtcfStdAlnRep2.bam.bai
wgEncodeUwTfbsHpafCtcfStdAlnRep1.bam
wgEncodeUwTfbsHpafCtcfStdAlnRep1.bam.bai
wgEncodeUwTfbsHpafCtcfStdAlnRep2.bam
wgEncodeUwTfbsHpafCtcfStdAlnRep2.bam.bai
wgEncodeUwTfbsHpfCtcfStdAlnRep1.bam
wgEncodeUwTfbsHpfCtcfStdAlnRep1.bam.bai
wgEncodeUwTfbsHpfCtcfStdAlnRep2.bam
wgEncodeUwTfbsHpfCtcfStdAlnRep2.bam.bai
wgEncodeUwTfbsHreCtcfStdAlnRep1.bam
wgEncodeUwTfbsHreCtcfStdAlnRep1.bam.bai
wgEncodeUwTfbsHreCtcfStdAlnRep2.bam
wgEncodeUwTfbsHreCtcfStdAlnRep2.bam.bai
wgEncodeUwTfbsHrpeCtcfStdAlnRep1.bam
wgEncodeUwTfbsHrpeCtcfStdAlnRep1.bam.bai
wgEncodeUwTfbsHrpeCtcfStdAlnRep2.bam
wgEncodeUwTfbsHrpeCtcfStdAlnRep2.bam.bai
wgEncodeUwTfbsHuvecCtcfStdAlnRep1.bam
wgEncodeUwTfbsHuvecCtcfStdAlnRep1.bam.bai
wgEncodeUwTfbsHuvecCtcfStdAlnRep2.bam
wgEncodeUwTfbsHuvecCtcfStdAlnRep2.bam.bai
wgEncodeUwTfbsHvmfCtcfStdAlnRep1.bam
wgEncodeUwTfbsHvmfCtcfStdAlnRep1.bam.bai
wgEncodeUwTfbsHvmfCtcfStdAlnRep2.bam
wgEncodeUwTfbsHvmfCtcfStdAlnRep2.bam.bai
wgEncodeUwTfbsNhdfneoCtcfStdAlnRep1.bam
wgEncodeUwTfbsNhdfneoCtcfStdAlnRep1.bam.bai
wgEncodeUwTfbsNhdfneoCtcfStdAlnRep2.bam
wgEncodeUwTfbsNhdfneoCtcfStdAlnRep2.bam.bai
wgEncodeUwTfbsNhekCtcfStdAlnRep1.bam
wgEncodeUwTfbsNhekCtcfStdAlnRep1.bam.bai
wgEncodeUwTfbsNhekCtcfStdAlnRep2.bam
wgEncodeUwTfbsNhekCtcfStdAlnRep2.bam.bai
wgEncodeUwTfbsNhlfCtcfStdAlnRep1.bam
wgEncodeUwTfbsNhlfCtcfStdAlnRep1.bam.bai
wgEncodeUwTfbsRptecCtcfStdAlnRep1.bam
wgEncodeUwTfbsRptecCtcfStdAlnRep1.bam.bai

wgEncodeUwTfbsRptecCtcfStdAlnRep2.bam
wgEncodeUwTfbsRptecCtcfStdAlnRep2.bam.bai
wgEncodeUwTfbsSaecCtcfStdAlnRep1.bam
wgEncodeUwTfbsSaecCtcfStdAlnRep1.bam.bai
wgEncodeUwTfbsSaecCtcfStdAlnRep2.bam
wgEncodeUwTfbsSaecCtcfStdAlnRep2.bam.bai
wgEncodeUwTfbsWi38CtcfStdAlnRep1.bam
wgEncodeUwTfbsWi38CtcfStdAlnRep1.bam.bai
wgEncodeUwTfbsWi38CtcfStdAlnRep2.bam
wgEncodeUwTfbsWi38CtcfStdAlnRep2.bam.bai

We analyzed the highest intensity CTCF sites to increase the chances that the sequencing reads span the SNP, so we can discriminate between bound alleles. We looked exclusively in normal-karyotype cell lines that were heterozygous at each locus to reduce the chances that copy-number variations (i.e. aneuploidy) of alleles would bias our analysis. In short, we took all reads that spanned the SNP and used samtools mpileup to call variants at the SNP. The code is provided here in `R` script.

```
samtools.mpileup <-function(functionstring="/usr/local/bin/samtools/bin/\
    samtools", snps= 'all_SNPS.txt' , bam = '\
    wgEncodeUwTfbsHbmecCtcfStdAlnRep1.bam', fasta = 'hg19.fa', opt.string="\
    mpileup -l") {
out = tempfile()
options(scipen =99) # not to use scientific notation when writing out

command=paste(functionstring, opt.string, snps, "-Bf", fasta, bam, ">", out\
    , sep=" ")
cat(command,"\n")
try(system(command))

res=read.table(out,header=F, comment.char='', sep = '\t')
unlink(out)
return(res)
}

all.bams <- function(path.dir = 'HMEC_bam',  snps= 'all_SNPS.txt', fasta = \
    'hg19.fa') {
vec.names = c('chr','pos','ref', 'coverage', 'alleles', 'qual', 'cells')
#create a data frame for the results
df = data.frame(matrix(ncol = 7, nrow = 0))
for (mod.bam in Sys.glob(file.path(path.dir, "*.bam"))) {
factor.name = strsplit(strsplit(mod.bam, "/")[[1]][length(strsplit(mod.bam,\
     "/")[[1]])], '\\.')[[1]][1]
factor.name = strsplit(factor.name, "wgEncodeUwTfbs")[[1]][2]
replicate.name = strsplit(factor.name, "Aln")[[1]][2]
factor.name = strsplit(factor.name, "Ctcf")[[1]][1]
factor.name = paste(factor.name, '_', replicate.name, sep ='')
print(factor.name)
sam.out = samtools.mpileup(bam = mod.bam, snps = snps)

x = cbind(sam.out, factor.name)
colnames(x) = vec.names
df = rbind(df, x)
}
t = apply(df, 1, function(df) string.counter(df[5], 'T') + string.counter(\
    df[5], 't') )
c = apply(df, 1, function(df) string.counter(df[5], 'C') + string.counter(\
    df[5], 'c') )
```

```
a = apply(df, 1, function(df) string.counter(df[5], 'A') + string.counter(\
    df[5], 'a') )
g = apply(df, 1, function(df) string.counter(df[5], 'G') + string.counter(\
    df[5], 'g') )
df[,8] = a
df[,9] = c
df[,10] = g
df[,11] = t
colnames(df) = c(colnames(df)[1:7], 'A','C','G','T')

for (i in 1:nrow(df)) {
if (df[i,3] == 'c' | df[i,3] == 'C') {
count = string.counter(df[i,5], '\\.') + string.counter(df[i,5], '\\,')
df[i,'C'] = count
}
if (df[i,3] == 'a' | df[i,3] == 'A') {
count = string.counter(df[i,5], '\\.') + string.counter(df[i,5], '\\,')
df[i,'A'] = count
}
if (df[i,3] == 't' | df[i,3] == 'T') {
count = string.counter(df[i,5], '\\.') + string.counter(df[i,5], '\\,')
df[i,'T'] = count
}
if (df[i,3] == 'g' | df[i,3] == 'G') {
count = string.counter(df[i,5], '\\.') + string.counter(df[i,5], '\\,')
df[i,'G'] = count
}
}

return(df)
}

string.counter<-function(strings, pattern){
counts<-NULL
for(i in 1:length(strings)){
counts[i]<-length(attr(gregexpr(pattern,strings[i])[[1]], "match.length")[\
    attr(gregexpr(pattern,strings[i])[[1]], "match.length")>0])
}
return(counts)
}

geno = all.bams(snps = 'ctcf_rs_sllele_inputs.txt')

#one set
x= geno[(geno[,2] == 5672019 & geno$T > 1 & geno$C > 1) | (geno[,2] ==  \
    18572834 & geno$T > 1 & geno$C > 1),]
x$C.frac = x$C/(x$C + x$T)
x$T.frac = x$T/(x$C + x$T)

counts.rs4414128 = rbind(x[x[,2] == 5672019,]$C.frac, x[x[,2] == 5672019,]\
    $T.frac)
colnames(counts.rs4414128) = x[x[,2] == 5672019,]$cells

counts.rs4414128 = counts.rs4414128[,order(-counts.rs4414128[1,])]


pdf("barchart.CTCF.allele_rs4414128.pdf", width=14, height=6)
par(mar = c(17, 4, 2, 2) + 0.2)
print(barplot(counts.rs4414128, main="CTCF allele-specific ChIP-seq at \
```

```
    rs4414128",
xlab="ENCODE Cell Line", col=c("red","grey40"), las = 2, legend = c("C", "T\
    ")
))
dev.off()

x= geno[(geno[,2] == 5672019 & geno$T > 1 & geno$C > 1) | (geno[,2] ==  \
    18572834 & geno$T > 1 & geno$C > 1),]

counts.rs4414128 = rbind(x[x[,2] == 5672019,]$C, x[x[,2] == 5672019,]$T)
colnames(counts.rs4414128) = x[x[,2] == 5672019,]$cells

counts.rs4414128 = counts.rs4414128[,order(-(counts.rs4414128[1,] + counts.\
    rs4414128[2,]))]


pdf("barchart.CTCF.allele_rs4414128_raw.pdf", width=14, height=6)
par(mar = c(17, 4, 2, 2) + 0.2)
print(barplot(counts.rs4414128, main="CTCF allele-specific ChIP-seq at \
    rs4414128",
xlab="ENCODE Cell Line", ylab = "allele-specific read count", col=c("red","\
    grey40"), las = 2, legend = c("C", "T")
))
dev.off()

#next set
x= geno[(geno[,2] == 18572834 & geno$T > 1 & geno$C > 1) | (geno[,2] ==  \
    18572834 & geno$T > 1 & geno$C > 1),]
x$C.frac = x$C/(x$C + x$T)
x$T.frac = x$T/(x$C + x$T)

counts.rs8103622 = rbind(x[x[,2] == 18572834,]$C.frac, x[x[,2] == \
    18572834,]$T.frac)
colnames(counts.rs8103622) = x[x[,2] == 18572834,]$cells

counts.rs8103622 = counts.rs8103622[,order(-counts.rs8103622[1,])]


pdf("barchart.CTCF.allele_rs8103622.pdf", width=14, height=6)
par(mar = c(17, 4, 2, 2) + 0.2)
print(barplot(counts.rs8103622, main="CTCF allele-specific ChIP-seq at \
    rs8103622",
xlab="ENCODE Cell Line", col=c("red","grey40"), las = 2, legend = c("C", "T\
    ")
))
dev.off()

x= geno[(geno[,2] == 18572834 & geno$T > 1 & geno$C > 1) | (geno[,2] ==  \
    18572834 & geno$T > 1 & geno$C > 1),]

counts.rs8103622 = rbind(x[x[,2] == 18572834,]$C, x[x[,2] == 18572834,]$T)
colnames(counts.rs8103622) = x[x[,2] == 18572834,]$cells

counts.rs8103622 = counts.rs8103622[,order(-(counts.rs8103622[1,]+counts.\
    rs8103622[2,]))]


pdf("barchart.CTCF.allele_rs8103622_raw.pdf", width=14, height=6)
par(mar = c(17, 4, 2, 2) + 0.2)
```

```
print(barplot(counts.rs8103622, main="CTCF allele-specific ChIP-seq at \
    rs8103622",
xlab="ENCODE Cell Line", ylab = "allele-specific read count", col=c("red","\
    grey40"), las = 2, legend = c("C", "T")
))
dev.off()
```

5.6 TFBS polymorphism and the expressions of breast cancer TCGA tissue phenotype

Based on the eQTL analysis, we picked several representative significant SNP-gene pairs and plotted boxplots to show their expression differences among genotype variants in the solid normal/tumor breast cancer tissue of patients. We pulled out the genotype of TCGA patient from the vcf files imputed from section 5.4.2. We retained only the allele information instead of the dosage from the following bash script.

```
#run the bash script locally
#sh ~/snps_egenes_boxplot_ref_alt.sh
dir=~/fastQTL

declare -a chrarray=("19" "19" "19" "19")
declare -a snparray=("rs4802200" "rs4808136" "rs7251653" "rs10402727")
declare -a corarray=("44289518" "18618867" "18608283" "52370379")

i=0
for chr in "${chrarray[@]}"
do
    echo chr:$chr
    snp=${snparray[$i]}
    echo snp:$snp
    cor=${corarray[$i]}
    echo $cor

    vcftools --gzvcf $dir/chr${chr}_${snp}_genotype.vcf.gz --out chr${chr}\
    _${snp}_single --chr $chr --from-bp $cor --to-bp $cor --maf 0.05 --hwe 1\
    e-6 --remove-filtered-geno-all --recode
    ref=$(head chr${chr}_${snp}_single.recode.vcf | tail -1 | cut -f4)
    echo ref:$ref

    alt=$(head chr${chr}_${snp}_single.recode.vcf | tail -1 | cut -f5)
    echo alt:$alt

    length=$(head -1 chr${chr}_${snp}_single.recode.vcf | grep -w 'TCGA' | \
    awk -F '\t' '{print NF}')
    echo length:$length

    cat chr${chr}_${snp}_single.recode.vcf | cut -f2,10-$length  | sed -e "\
    s/0\/0:[0-9].[0-9][0-9][0-9]:[0-9].[0-9][0-9][0-9],[0-9].[0-9][0-9][0-\
    9],[0-9].[0-9][0-9][0-9]/$ref$ref/g" -e "s/0\/1:[0-9].[0-9][0-9][0-9]:[0\
    -9].[0-9][0-9][0-9],[0-9].[0-9][0-9][0-9],[0-9].[0-9][0-9][0-9]/$ref$alt\
    /g" -e "s/1\/0:[0-9].[0-9][0-9][0-9]:[0-9].[0-9][0-9][0-9],[0-9].[0-9][0\
    -9][0-9],[0-9].[0-9][0-9][0-9]/$ref$alt/g" -e "s/1\/1:[0-9].[0-9][0-9][0\
    -9]:[0-9].[0-9][0-9][0-9],[0-9].[0-9][0-9][0-9],[0-9].[0-9][0-9][0-9]/\
    $alt$alt/g" | sed "s/-/\./g" > chr${chr}_${snp}_single_nodosage.vcf


      (( i = i +1 ))
```

```
done
```

Then we were able to plot the boxplots in R.

```
rm(list=ls())
sessionInfo()
setwd("~/snp_gene_pair")

chr=list(19,19,19,19)
snps=list('rs4802200','rs4808136','rs7251653','rs10402727')
genes=list('ZNF404','PGPEP1','PGPEP1','ZNF577')
cor=list(44289518,18618867,18608283,52370379)

x = read.csv('~/TCGA_BRCA_exp_HiSeqV2_PANCAN-2015-02-24/genomicMatrix', sep\
    ='\t')
row.names(x) = x$sample
x = x[,2:ncol(x)]
t.x=data.frame(t(x))

#tumor patients
t.x1=t.x[grep('.01$',row.names(t.x)),]
row.names(t.x1)=sapply(strsplit(row.names(t.x1),'.01$'),function(x) x[[1]])
t.x1$ID=rownames(t.x1)

#normal patients
t.x2=t.x[grep('.11$',row.names(t.x)),]
row.names(t.x2)=sapply(strsplit(row.names(t.x2),'.11$'),function(x) x[[1]])
t.x2$ID=rownames(t.x2)

for (i in 1:length(snps))
{
  snp=read.table(paste0('chr',chr[i],'_',snps[i],'_single_nodosage.vcf'),\
    header=T,comment.char="")[,-1]
  snp=data.frame(t(snp))

  egene=t.x1[names(t.x1)%in%unlist(genes[i])]

  snp.egene=merge(snp,egene,by='row.names')

  pdf(paste0('chr',chr[i],'_',snps[i],'_',genes[i],'_tumor','.pdf'),width\
    =6,height=5)
  b=boxplot(snp.egene[,3]~snp.egene[,2],plot=0)

  boxplot(snp.egene[,3]~snp.egene[,2],xlab='Genotype',ylab='Gene Expression\
    ',
          main=unlist(genes[i]))
  text(1:length(b$n), b$stats[5,]+1, paste("n=", b$n))

  stripchart(snp.egene[,3]~snp.egene[,2], vertical = TRUE,
             method = "jitter", add = TRUE, pch = 20, cex=0.5,col = 'blue')
  mtext(snps[i],side=4)
  dev.off()

  egene=t.x2[names(t.x2)%in%unlist(genes[i])]

  snp.egene=merge(snp,egene,by='row.names')

  pdf(paste0('chr',chr[i],'_',snps[i],'_',genes[i],'_normal','.pdf'),width\
    =6,height=5)
```

```
  boxplot(snp.egene[,3]~snp.egene[,2],xlab='Genotype',ylab='Gene Expression\
    ',
          main=unlist(genes[i]))
  stripchart(snp.egene[,3]~snp.egene[,2], vertical = TRUE,
              method = "jitter", add = TRUE, pch = 20, cex=0.5,col = 'blue')
  mtext(snps[i],side=4)
  dev.off()

}
```

We also show the R code for rs11540855-ANKLE1 boxplot here.

```
rm(list=ls())
sessionInfo()

snp=read.table('chr19_rs11540855_single_nodosage.vcf',header=T,comment.char\
    ="")[,-1]
snp=data.frame(t(snp))

x = read.csv('~/TCGA_BRCA_exp_HiSeqV2_PANCAN-2015-02-24/genomicMatrix', sep\
    ='\t')
row.names(x) = x$sample
x = x[,2:ncol(x)]
t.x=data.frame(t(x))
#tumor patients
t.x1=t.x[grep('.01$',row.names(t.x)),]
row.names(t.x1)=sapply(strsplit(row.names(t.x1),'.01$'),function(x) x[[1]])
t.x1$ID=rownames(t.x1)

egene=t.x1[names(t.x1)%in%c('ANKLE1')]
snp.egene=merge(snp,egene,by='row.names')

snp.egene[,2]=gsub('AA','A/A',snp.egene[,2])
snp.egene[,2]=gsub('AG','A/G',snp.egene[,2])
snp.egene[,2]=gsub('GG','G/G',snp.egene[,2])
font=1
#reorder genotype
snp.egene[,2]=factor(snp.egene[,2],levels=c("A/A","A/G","G/G"))

b=boxplot(snp.egene[,3]~snp.egene[,2],plot=0)

pdf('rs11540855_ANKLE1_boxplot.pdf',width=6,height=5)
op <- par(mar=c(5, 6, 4, 2) + 0.1)
boxplot(snp.egene[,3]~snp.egene[,2],xlab="",ylab="Normalized Gene \
    Expression",
        main="eQTL rs11540855-ANKLE1",col="orange",cex.axis=font,cex.lab=\
    font,cex.main=font)
mtext(text=paste0("n=",b$n),side = 1,at=c(1,2,3),line=3,cex=font)
stripchart(snp.egene[,3]~snp.egene[,2], vertical = TRUE,
            method = "jitter", add = TRUE, pch = 20, cex=1,col = 'blue')
par(op)
dev.off()
```

6 Correlation analysis of patient survival data with TF expression from the Cancer Genome Atlas data

Kaplan-Meier plots are visual estimates of the survival of different groups of patients over time. Percent survival is on the Y-axis and time is on the X-axis; the steeper curve, the worse the survival outcome is over time. Kaplan-Meier plots can be generated by an automated process using R for each breast cancer subtype: "Luminal A", "Luminal B", "Her2+", "Normal", "Basal-like" subtype, and other unknown subtype. TF expression levels were measured by provisional RNA-seq data from The Cancer Genome Atlas (TCGA) originating from the breast cancer patient primary solid tumor samples, which was curated by UCSC (refer to section 5.4.1). Thresholds of expression were selected to show the most contrast between the two groups (High and Low expression groups), p-values were calculated using the log-rank test statistic and multiple testing corrected using the FDR (Benjamini and Hochberg, 1995). The script is coded in R.

```
rm(list=ls())

b = read.csv('TCGA_BRCA_exp_HiSeqV2_PANCAN-2015-02-24/clinical_data', sep =\
    '\t')
d<-data.frame(b$sampleID, b$AJCC_Stage_nature2012, b$X_OS, b$X_OS_IND, b$X_\
    RFS, b$X_RFS_IND);

#"PAM50_mRNA_nature2012"    "PR_Status_nature2012"     "RPPA_Clusters_\
    nature2012"
subtypesX = b$PAM50Call_RNAseq

i1<-grep('LumA', subtypesX)
i2<-grep('LumB', subtypesX)
i3<-grep('Her2', subtypesX)
i4<-grep('Basal', subtypesX)
i5<-grep('Normal', subtypesX)

subtypes1new<-rep('NotKnown', length(subtypesX));
subtypes1new[i1]<-'Luminal A';
subtypes1new[i2]<-'Luminal B';
subtypes1new[i3]<-'HER2 type';
subtypes1new[i4]<-'Basal-like';
subtypes1new[i5]<-'Normal-like';

#what types of tumors are there:
unique(as.character(lapply(strsplit(as.character(d[,1]), split="-"), "[", \
    4)))
#01 primary solid tumor
#11 solid tissue normal
#06 Metastatic
#https://tcga-data.nci.nih.gov/datareports/codeTablesReport.htm?codeTable=\
    Sample%20type

d1<-data.frame(d, subtypes1new)

#select primary solid
d1 <- d1[grep('.01$', d1[,1]),]
colnames(d1)<-c(colnames(d1)[1:(ncol(d1)-1)], 'SUBTYPE');

d1b<-data.frame(d1$b.X_RFS, d1$b.X_RFS_IND, d1$b.X_OS, d1$b.X_OS_IND, d1$\
    SUBTYPE)
colnames(d1b)<-c('DFS_MONTHS', 'DFS_STATUS', 'OS_MONTHS', 'OS_STATUS', '\
    SUBTYPE')
```

```
rownames(d1b)<-gsub('-', '.', d1$b.sampleID)

####read in RNA-seq expression data#####
x = read.csv('TCGA_BRCA_exp_HiSeqV2_PANCAN-2015-02-24/genomicMatrix', sep='\
    \t')
row.names(x) = x[,1]
x = x[,2:ncol(x)]
x1 = x[,colnames(x) %in% rownames(d1b) ]
t.x1 = t(x1)

all.data = merge(d1b, t.x1, by=0, all=FALSE)

km.panel <- function(x,y,type,mark.time=T,...){
  na.part <- is.na(x)|is.na(y)
  x <- x[!na.part]
  y <- y[!na.part]
  if (length(x)==0) return()
  fit <- survfit(Surv(x,y)~1)
  if (mark.time){
    cens <- which(fit$time %in% x[y==0])
    panel.xyplot(fit$time[cens], fit$surv[cens], type="p",...)
  }
  panel.xyplot(c(0,fit$time), c(1,fit$surv),type="s",...)
}

logrank.panel=function(x,y,subscripts,groups,...){
  lr <-  survdiff(Surv(x,y)~groups[subscripts])
  otmp <- lr$obs
  etmp <- lr$exp
  df <- (sum(1 * (etmp > 0))) - 1
  p <- 1 - pchisq(lr$chisq, df)
  print("pvalue")
  print(p)
  line = grep(p,p.fdr[,3])
  print("line")
  print(line)
  print("fdr")
  print(p.fdr[,2][line])
  p=p.fdr[,2][line]
  p.text <- paste("p=", signif(p, 2))
  grid.text(p.text, 0.95, 0.05, just=c("right","bottom"))
  grid.text(paste('High Expression (', sum(groups[subscripts]),')', sep =''\
    ), 0.05,0.11,just=c("left","bottom"), gp=gpar(col="red"))
  grid.text(paste('Low Expression (',sum(groups[subscripts] == FALSE),')', \
    sep =''), 0.05,0.05,just=c("left","bottom"), gp=gpar(col="blue"))
  panel.superpose(x=x,y=y,subscripts=subscripts,groups=groups,...)
}

library(cgdsr)
library(hash)
library(survival)
library(lattice)
library(grid)

###subsetting all.data by subtypes###
all.data.lumA = subset(all.data,SUBTYPE=='Luminal A')
all.data.lumB = subset(all.data,SUBTYPE=='Luminal B')
all.data.her2 = subset(all.data,SUBTYPE=='HER2 type')
all.data.basal = subset(all.data,SUBTYPE=='Basal-like')
```

```
all.data.normal = subset(all.data,SUBTYPE=='Normal-like')
all.data.other = subset(all.data,SUBTYPE=='NotKnown')

listalldata = list(all.data.lumA,all.data.lumB,all.data.her2,
                   all.data.basal,all.data.normal,all.data.other)
listsubtype = list('lumA','lumB','her2','basal','normal','unknown')

TFs=read.table("160902_5_brca_celines_405communities_map_list_TF_\
    handcuration.csv",sep=",")
genelist=unique(as.character(unlist(TFs)))
genelist=genelist[genelist!=""]

theta=numeric()

fdr.adjust.type=data.frame(matrix(unlist(genelist),ncol=1,nrow=length(\
    genelist)))
colnames(fdr.adjust.type)='TF'
for (k in 1:length(listalldata))
{
  newdata=as.data.frame(listalldata[k])
  teststep=0.10; # should be between 0 and 0.5
  theta_list=seq(from=0+teststep,to=1-teststep,by=teststep);
  pval_res=rep(1,length(theta_list));

  newgenedata=cbind(newdata[,1:5], newdata[,colnames(newdata)%in%genelist])

  ###subsetting newdata by genelist#####
  all.km.df = data.frame(matrix(ncol = 7, nrow = 0))
  colnames(all.km.df) = c('patient','DFS_MONTHS','DFS_STATUS','OS_MONTHS','\
    OS_STATUS','TF','EXPRESSION')

  rawp=numeric()
  for (i in 1:length(genelist))
  {
    tryCatch({#this skip when errors occur, so the looping can carry on
      for (jj in 1:length(theta_list))
      {
        br<-newgenedata;
        br$OS_STATUS=newgenedata$OS_STATUS=="1";
        br$DFS_STATUS=newgenedata$DFS_STATUS=="1";
        thr<-quantile(br[[genelist[i]]], probs=theta_list[jj], na.rm = TRUE\
    )
        br[[genelist[i]]]<-br[[genelist[i]]] >= thr;
        fit <- survfit(Surv(OS_MONTHS,OS_STATUS) ~ get(genelist[i]), data=\
    br);
        fittest <-survdiff(Surv(OS_MONTHS, OS_STATUS) ~ get(genelist[i]), \
    data=br);
        pval<-1-pchisq(fittest$chisq,1);
        pval_res[jj]<-pval;
      }

      minind<-which(min(pval_res)==pval_res);

      theta[i]<-theta_list[min(minind)];
      pval<-pval_res[minind];#here is the pvalue put on the KM plot without\
     fdr
      newp=pval
      names(newp)=genelist[i]
      rawp=c(rawp,newp)
```

```
    gene.exp = vector(mode = 'logical', length = nrow(newgenedata))
    gene.exp[newgenedata[[genelist[i]]] >= quantile(newgenedata[[genelist\
  [i]]], probs=theta[i], na.rm = TRUE) ] = TRUE
    newgenedata[[genelist[i]]] = gene.exp
    newgenedata$OS_STATUS[newgenedata$OS_STATUS == 1] = TRUE
    newgenedata$OS_STATUS[newgenedata$OS_STATUS == 0] = FALSE
    newgenedata$OS_MONTHS[newgenedata$OS_MONTHS == 1] = TRUE
    newgenedata$OS_MONTHS[newgenedata$OS_MONTHS == 0] = FALSE

    lattice.format = cbind(newgenedata[,1:5], genelist[i], newgenedata[[\
  genelist[i]]])
    colnames(lattice.format) = c('patient','DFS_MONTHS','DFS_STATUS','OS_\
  MONTHS','OS_STATUS','TF','EXPRESSION')
    all.km.df = rbind.data.frame(all.km.df, lattice.format)
   }, error=function(e){cat("ERROR :",conditionMessage(e), "\n")})
   print(paste0('TF',i,genelist[i]))
   tryCatch({#this skip when errors occur, so the looping can carry on
    print(paste0('theta',theta[i]))
   }, error=function(e){cat("ERROR :",conditionMessage(e), "\n")})
}

fdr.adjust=p.adjust(rawp,method="fdr")
t.fdr.adjust=data.frame(fdr.adjust,names(fdr.adjust))
colnames(t.fdr.adjust)=c(paste0(listsubtype[k],'.fdr'),'TF')

rawp=data.frame(rawp,names(rawp))
colnames(rawp)=c(paste0(listsubtype[k],'.p'),'TF')

p.fdr = merge(t.fdr.adjust,rawp,by="TF")

save(p.fdr,all.km.df,km.panel,logrank.panel,listsubtype,k,file=paste0(\
  listsubtype[k],'.RData'))

fdr.adjust.type=merge(fdr.adjust.type,p.fdr,by='TF',all.x=T)

figurename=paste0('KMplot_',listsubtype[k],".pdf")
pdf(figurename, width=4, height=3.4 * length(unique(all.km.df$TF)))
col.lines = c('red', 'blue')
print(paste0('TFlength',length(unique(all.km.df$TF))))

figure=
   xyplot(all.km.df$OS_STATUS ~ all.km.df$OS_MONTHS | all.km.df$TF, data=\
  all.km.df, groups=all.km.df$EXPRESSION,
          lty= 1,
          aspect = 1,
          pch= "+",
          cex=1.5,
          between=list(y=0.5, x=0.5),
          col = rev(col.lines),
          strip = function(..., which.panel, bg) {
            bg.col = c("grey85")
            strip.default(..., which.panel = which.panel, bg = rep(bg.col,\
   length = which.panel)[which.panel])
          },
          ylab = "Survival",
          xlab = "Time (days)",
          panel.groups=km.panel,
          panel=logrank.panel,
```

```
            layout=c(1,length(unique(all.km.df$TF))),
            scales = list(x = list(alternating = FALSE),
                          y = list(alternating = FALSE)),
            main=paste0('Subtype: ',listsubtype[k]))

  print(figure)
  dev.off()
  print(paste0('type',k))
}

save(fdr.adjust.type,file="fdr_subtypes.RData")
write.table(fdr.adjust.type,file="fdr_subtypes.txt",sep="\t",col.names=T,\
    row.names=F,quote=F)

#select TFs with fdr < 0.05
fdr.adjust.type.lumA.sig=na.omit(fdr.adjust.type[fdr.adjust.type$lumA.fdr <\
     0.05,c(1,2,3)])
fdr.adjust.type.lumB.sig=na.omit(fdr.adjust.type[fdr.adjust.type$lumB.fdr <\
     0.05,c(1,4,5)])
fdr.adjust.type.her2.sig=na.omit(fdr.adjust.type[fdr.adjust.type$her2.fdr <\
     0.05,c(1,6,7)])
fdr.adjust.type.basal.sig=na.omit(fdr.adjust.type[fdr.adjust.type$basal.fdr\
     < 0.05,c(1,8,9)])
fdr.adjust.type.normal.sig=na.omit(fdr.adjust.type[fdr.adjust.type$normal.\
    fdr < 0.05,c(1,10,11)])
fdr.adjust.type.unknown.sig=na.omit(fdr.adjust.type[fdr.adjust.type$unknown\
    .fdr < 0.05,c(1,12,13)])
for (i in 1:length(listsubtype))
{
  write.table(get(paste0('fdr.adjust.type.',listsubtype[i],'.sig')),file=\
    paste0('fdr_',listsubtype[i],'.txt'),
                sep="\t",col.names=T,row.names=F,quote=F)
}
```

Appendix

A Bash shell script: add a function to the executable path

Let use the "fastq-dump" function (http://www.ncbi.nlm.nih.gov/Traces/sra/sra.cgi?view=toolkit_doc&f=fastq-dump) for example. Download "SRA Toolkig" from the following web-page: http://www.ncbi.nlm.nih.gov/Traces/sra/sra.cgi?view=software. Install the function using command-line according to instruction ("README" or "INSTALL" file) being provided by the developer. "fastq-dump" function is included in the "bin" folder being downloaded. Add this function using following command line.

```
#This line reflects all current functions included in the path
echo $PATH
#This line shows all files included in the PATH's folder
ls /usr/local/bin
sudo mkdir /usr/local/bin/sratoolkit.2.5.2-mac64
# Move all the functions to the PATH's folder
sudo mv ~/Downloads/sratoolkit.2.5.2-mac64/bin/* /usr/local/bin/sratoolkit\
    .2.5.2-mac64/
```

Next, add the path of "fastq-dump" (/usr/local/bin/sratoolkit.2.5.2-mac64) to ".bash_profile" file, which is the path file. We can edit this file using EMACS (https://www.gnu.org/software/emacs/). Paths of different functions are separated by colon (:).

```
emacs ~/.bash\_profile
#In the bash\_profile file, add the function path
/usr/local/bin:/usr/bin:/bin:/usr/sbin:/sbin:/usr/local/bin/sratoolkit\
    .2.5.2-mac64
```

B R script: calculate the probes with highest intensity

```
library(bigWig)

calc.highest.probe <- function(sites, wig, window = 30) {
  N = dim(sites)[1]
  result = vector(mode="integer", length=N)
  chromo = vector(mode="numeric", length=N)
  chromo.end = vector(mode="numeric", length=N)

  vector(mode="numeric", length=N)
  cur.chrom = NULL
  cur.wig = NULL

  cur.wig = load.bigWig(wig)

  stopifnot(!is.null(cur.wig))

  for (i in 1:N) {
    chrom = as.character(sites[i,1])

    if (is.null(cur.chrom) || cur.chrom != chrom) {

      cur.chrom = chrom
    }
```

```
    qStart = as.numeric(as.character(sites[i, 2]))
    qEnd = as.numeric(as.character(sites[i, 3]))
    center = (qStart + qEnd)/2
    data = query.bigWig(cur.wig, cur.chrom, qStart, qEnd)

    if (!is.null(data)) {
      maximum=max(data[,3])
      newvar = subset(data, data[,3] == maximum)
      min.distance=min(abs(((newvar[,1]+newvar[,2])/2)-center))
      num.rows = dim(newvar)[1]
      if (num.rows != 0) {
        for (j in 1:num.rows) {
          if (min(abs(((newvar[j,1]+newvar[j,2])/2)-center)) == min.\
    distance) {
            highest.closest.probe = newvar[j,]
          }
        }
      }
      highest.closest.probe = matrix(highest.closest.probe,nrow=1, ncol=3)

      start = round((highest.closest.probe[,1]+highest.closest.probe[,2])/\
    2) - window
      end = round((highest.closest.probe[,1]+highest.closest.probe[,2])/2) \
    + window
      result[i] = highest.closest.probe[,3]
      chromo[i] = round((highest.closest.probe[,1] + highest.closest.probe\
    [,2])/2)
    } else {
      chromo[i] = round((qStart + qEnd)/2)
    }
  }
  unload.bigWig(cur.wig)
  df = cbind(as.character(sites[,1]),matrix(chromo - window,nrow=N), matrix\
    (chromo + window,nrow=N))
  df= as.data.frame(df)
  colnames(df) = c('chr', 'start', 'end')
  df[,2] = as.numeric(as.character(df[,2]))
  df[,3] = as.numeric(as.character(df[,3]))
  return(df)
}

hotspots = 'Hotspots_Either_E2_condition.merge.broadPeak'
highest.hotspot = calc.highest.probe(read.table(hotspots), 'Either_E2_\
    condition_MCF7.bigWig', window =60)

x = bed.region.probeQuery.bigWig(load.bigWig('Either_E2_condition_MCF7.\
    bigWig'), highest.hotspot)
inten.highest.hotspot = cbind(highest.hotspot,x)
colnames(inten.highest.hotspot) = c('chr','start','end','intensity')
write.table(inten.highest.hotspot, file = 'inten_highest_hotspot.bed', \
    quote=F, row.names=F, col.names=F, sep= '\t')
```

C Bash shell script: *de novo* motif finding with one-stop iteration and MEME-ChIP

```
#!/bin/bash
#submit this job locally
```

```
#needs to be changed
#sh appendix_C_MCF7.sh

#needs to be changed
dir=~/Desktop/Iterate_MCF7_hotspot_onestop/

con=~/iteration/database
#cd $dir
start=`date +%s`
first=1
i=$first
k=2000
a=1
j=1

name[0]=brca_int.$k.top
motif[0]=
#specify the background file's order
b=3
subdir=top_int_${k}_b${b}_MAST
mkdir $dir/$subdir
cp $dir/inten_highest_hotspot.bed $dir/$subdir/${name[0]}_r0_${motif[0]}.\
    hotspot.bed

#top k intensity
sort -k4 -r -n $dir/$subdir/${name[0]}_r0_${motif[0]}.hotspot.bed | head -\
    $k > $dir/$subdir/${name[0]}_r0_${motif[0]}.bed

wc[0]=$(wc -l $dir/$subdir/${name[0]}_r0_${motif[0]}.hotspot.bed | awk '{\
    print $1}')
echo wordcount of the hotspot.bed file: wc[0]_${wc[0]}

#extracting the sequence for each genomic coordinate
fastaFromBed -fi ~/hg19.fa -bed $dir/$subdir/${name[0]}_r0_${motif[0]}.bed \
    -fo $dir/$subdir/${name[0]}_r0_${motif[0]}.fasta

while [[ ( ${wc[$i-1]} -gt 20000 )  && ( $(($k*$[$j+1])) -lt 20000 ) ]]
do
    echo round$i
    name[$i]=$(echo ${name[0]}_r$[$i-1]_${motif[$i-1]})
    echo round{$i}_{name}_${name[$i]}

    #generate second-order bacground model
    fasta-get-markov -m $b  $dir/$subdir/${name[$i]}.fasta  $dir/$subdir/${\
    name[$i]}.fasta.$b.bfile

    #MEME
    meme $dir/$subdir/${name[$i]}.fasta -o $dir/$subdir/${name[$i]}.\
    meme_output -dna -nmotifs 1 -minw 5 -maxw 20 -revcomp -evt 0.01 -maxsize\
     10000000 -bfile $dir/$subdir/${name[$i]}.fasta.$b.bfile

    #tomtom
    tomtom -no-ssc -o $dir/$subdir/${name[$i]}.tomtom_output -verbosity 1 -\
    min-overlap 5 -mi 1 -dist pearson -evalue -thresh 0.01 $dir/$subdir/${\
    name[$i]}.meme_output/meme.txt $con/combined_motif_db.txt

    motif[$i]=$(awk 'FNR == 2 {print $2}' $dir/$subdir/${name[$i]}.\
    tomtom_output/tomtom.txt)
```

```
    #you can check the content of the variable by
    echo round{$i}_{motif}_${motif[$i]}
    echo ${motif[$i]} >> $dir/$subdir/motifs.txt

    #MAST
    mkdir $dir/$subdir/${name[$i]}.mast_output
    mast $dir/$subdir/${name[$i]}.meme_output/meme.txt $dir/\
   inten_highest_hotspot.fasta -hit_list -m 1 >  $dir/$subdir/${name[$i]}.\
   mast_output/mast_hotspots.txt
    #python MAST_to_bed, output file is ${name[$i]}_mast_hotspots.bed
    python  $con/MAST_bed_conversion.py -i $dir/$subdir/${name[$i]}.\
   mast_output/mast_hotspots.txt -o $dir/$subdir/${name[$i]}.mast_output/\
   mast_hotspots.bed

    #composite analysis
    python $con/MEME_matrix_wrapper.py -i $dir/$subdir/${name[$i]}.\
   meme_output/meme.txt -o $dir/$subdir/${name[$i]}.meme_output/minimal

    #see where motif instances overlap with DNase peaks
    intersectBed -v -wa -a $dir/$subdir/${name[0]}_r$[$i-1]_${motif[$i-1]}.\
   hotspot.bed -b $dir/$subdir/${name[$i]}.mast_output/mast_hotspots.bed > \
     $dir/$subdir/${name[0]}_r${i}_${motif[$i]}.hotspot.bed
    wc[$i]=$(wc -l $dir/$subdir/${name[0]}_r${i}_${motif[$i]}.hotspot.bed |\
     awk '{print $1}')
    echo wordcount of the hotspot.bed file: wc[$i]_${wc[$i]}

    if [[ ${wc[$i]} == ${wc[$i-1]} ]]
    then
        echo notice: move to top_$[k*j+1]_to_$[k*(j+1)]
    sort -k4 -r -n $dir/$subdir/${name[0]}_r${i}_${motif[$i]}.hotspot.bed |\
     head -$(($k*$[$j+1])) | tail -$k > $dir/$subdir/${name[0]}_r${i}_${\
   motif[$i]}.bed
    (( j =j + 1 ))
    else
    echo continue as always
    j=1
    #select top k intensity
    sort -k4 -r -n $dir/$subdir/${name[0]}_r${i}_${motif[$i]}.hotspot.bed |\
     head -$k > $dir/$subdir/${name[0]}_r${i}_${motif[$i]}.bed

       if [[ ${motif[$i]} == "" ]]
       then
           echo r${i}_motif_orphan$a >> $dir/$subdir/ophans.txt
           ((a = a + 1))
        else
           echo motif_${motif[$i]} found within our database
       fi

    #rename all the files with the round number $i and the motif found in \
   round $i
    ##rename all meme output files
    for file in $dir/$subdir/${name[$i]}.meme_output/*
    do
    mv $file ${file%.*}_r${i}_${motif[$i]}.${file##*.}
    done
    #rename all tomtom output files
    for file in $dir/$subdir/${name[$i]}.tomtom_output/*
    do
    mv $file ${file%.*}_r${i}_${motif[$i]}.${file##*.}
```

```
    done

    #renmae all mast output files
    for file in $dir/$subdir/${name[$i]}.mast_output/*
    do
    mv $file ${file%.*}_r${i}_${motif[$i]}.${file##*.}
    done

    fi

   #extract the sequence for each genomic coordinate--bed into fasta file
    fastaFromBed -fi ~/hg19.fa -bed $dir/$subdir/${name[0]}_r${i}_${motif[\
   $i]}.bed -fo $dir/$subdir/${name[0]}_r${i}_${motif[$i]}.fasta

    ((i = i + 1))
done

end=`date +%s`
runtime=$((end-start))
echo $runtime

mkdir $dir/withmotif/
cp $dir/$subdir/*.meme_output/minimal_meme_r*_*.txt $dir/withmotif/

#check the size of all the minimal_meme_r*_*.txt files and make sure that \
   there is no file with no PWM
#if there is any minimal meme file with no PWM, remove them

#cacatenate all the minimal meme files
cat $dir/withmotif/* > $dir/meme_chip_minimal_meme.txt

#conduct mast on the minimal meme files
#make a mast_output folder for each text file
mkdir $dir/mast_output
mast $dir/meme_chip_minimal_meme.txt $dir/inten_highest_hotspot.fasta -\
   hit_list -mt 0.00001 > $dir/mast_output/mast_hotspots.txt
python $con/MAST_bed_conversion.py -i $dir/mast_output/mast_hotspots.txt -o\
    $dir/mast_output/mast_hotspots.bed
intersectBed -v -wa -a $dir/inten_highest_hotspot.bed -b $dir/mast_output/\
   mast_hotspots.bed > $dir/meme_chip.hotspot.bed
fastaFromBed -fi ~/hg19.fa -bed $dir/meme_chip.hotspot.bed -fo $dir/\
   meme_chip.hotspot.fasta

fasta-get-markov -m $b $dir/meme_chip.hotspot.fasta $dir/meme_chip.hotspot.\
   fasta.$b.bfile
fasta-center -len 120 <$dir/meme_chip.hotspot.fasta 1> $dir/seqs-centered-\
   120
fasta-dinucleotide-shuffle -f $dir/seqs-centered-120 -t -dinuc 1> $dir/seqs\
   -shuffled-120
fasta-subsample $dir/seqs-centered-120 2000 -rest $dir/seqs-discarded-2000 \
   1> $dir/seqs-sampled-2000
#run meme to find 50 motifs
meme $dir/seqs-sampled-2000 -oc $dir/meme_chip_meme_output -dna -mod zoops \
   -nmotifs 50 -minw 5 -maxw 20 -bfile $dir/meme_chip.hotspot.fasta.3.bfile\
    -revcomp -nostatus -maxsize 10000000
#run dreme to find the short-length motifs
dreme -v 1 -oc $dir/meme_chip_dreme_output -png -p $dir/seqs-centered-120 -\
   n $dir/seqs-shuffled-120
```

```
#process the memechip output
####memechip-meme
mkdir $dir/memechip_meme_individual
cp $dir/meme_chip_meme_output/meme.txt $dir/memechip_meme_individual/
#convert to minimal meme text file
python $con/MEME_matrix_wrapper.py -i $dir/memechip_meme_individual/meme.\
    txt -o $dir/memechip_meme_individual/meme_motif
rm $dir/memechip_meme_individual/meme.txt
#generate individual meme files
python $con/MEME_individual_from_db.py -i $dir/memechip_meme_individual/\
    meme_motif_meme.txt
rm $dir/memechip_meme_individual/meme_motif_meme.txt

#rerun it starting from here
#run tomtom and rename everything
cd ~
j=1
for file in $dir/memechip_meme_individual/*
do
    echo $file
    file1=${file%/*}
    file2=${file#*individual/meme_}
    file3=${file2%.txt}
    tomtom=$file1/${file3}_tomtom_output
    echo tomtom_$tomtom
    tomtom -verbosity 1 -o $tomtom -min-overlap 5 -dist pearson -evalue -\
    thresh 1 -no-ssc $file $con/combined_motif_db.txt
    tomtomtxt=$tomtom/tomtom.txt
    motifid=$(awk 'FNR == 2 {print $2}' $tomtomtxt)
    echo motifid_$motifid

    if [[ $motifid == "" ]]
then
    motifid=orphan$j
    echo motifid_$motifid
    echo $file3.orphan$j >> $dir/memechip_meme_individual/orphans.E1.txt
    ((j = j + 1))
else
    echo motifid_$motifid
    echo $motifid >> $dir/memechip_meme_individual/motifs.E1.txt
    fi

    newfile=$file1/motif_${motifid}_meme.txt
    mv $file $newfile
    for file in $tomtom/*
    do
        file1=${file%.*}
        file2=${file#*.}
        mv $file ${file1}_motif_${motifid}_meme.${file2}
        done
done

#mast
for file in $dir/memechip_meme_individual/motif_*.txt
do
 file1=${file%.txt}
 mkdir ${file1}.mast_output
 file2=${file1##*/}
 mast $file $dir/inten_highest_hotspot.fasta -hit_list -m 1 >  ${file1}.\
```

```
    mast_output/mast_hotspots_${file2}.txt
 python $con/MAST_bed_conversion.py -i ${file1}.mast_output/mast_hotspots_$\
    {file2}.txt
done


#########memechip_dreme
mkdir $dir/memechip_dreme_individual
cp $dir/meme_chip_dreme_output/dreme.txt  $dir/memechip_dreme_individual/
#eliminate everything line starting with "#"
grep -v "#" $dir/memechip_dreme_individual/dreme.txt > $dir/\
    memechip_dreme_individual/dreme_post.txt
#generate individual minimal meme files
cd $dir/memechip_dreme_individual
python  $con/MEME_individual_from_db.py -i $dir/memechip_dreme_individual/\
    dreme_post.txt
rm $dir/memechip_dreme_individual/dreme*.txt
cd ~

#run tomtom and rename everything
j=1
for file in $dir/memechip_dreme_individual/*.txt
do
echo $file
filename=${file%.txt}
tomtom -no-ssc -o $filename.E1.tomtom_output -verbosity 1 -min-overlap 5 -\
    mi 1 -dist pearson -evalue -thresh 1.0 $file $con/combined_motif_db.txt

tomtomtxt=$filename.E1.tomtom_output/tomtom.txt
motifid=$(awk 'FNR == 2 {print $2}' $tomtomtxt)
echo motifid_$motifid

if [[ $motifid == "" ]]
then
    motifid=orphan$j
    echo motifid_$motifid
    echo $filename.orphan$j >> $dir/memechip_dreme_individual/orphans.E1.\
    txt
    ((j = j + 1))
else
    echo motifid_$motifid
    echo $motifid >> $dir/memechip_dreme_individual/motifs.E1.txt
fi

for files in $filename.E1.tomtom_output/*
do
file1=${files%.*}
file2=${files##*.}
mv $files ${file1}_motif_${motifid}.${file2}
done

mv $file $dir/memechip_dreme_individual/motif_${motifid}_dreme.txt

#run mast for the dreme.txt files
mkdir $dir/memechip_dreme_individual/motif_${motifid}_dreme.mast_output
mast $dir/memechip_dreme_individual/motif_${motifid}_dreme.txt $dir/\
    inten_highest_hotspot.fasta -hit_list -m 1 >   $dir/\
    memechip_dreme_individual/motif_${motifid}_dreme.mast_output/\
    mast_hotspots_motif_${motifid}_dreme.txt
```

```
python $con/MAST_bed_conversion.py -i $dir/memechip_dreme_individual/\
    motif_${motifid}_dreme.mast_output/mast_hotspots_motif_${motifid}_dreme.\
    txt

done
```

D Python script: reformat MAST text file to BED file

```
#! /usr/bin/python
import sys
import getopt
def matrix(filename):
    infile=open(filename, 'r')
    print str.split(filename, '.txt')[0] + '.bed'
    outfile=open(str.split(filename, '.txt')[0] + '.bed', 'w')
    while 1:
        line = infile.readline()
        if not line: break
        splitline = line.split()
        if line.startswith('#'):
            continue
        else:
            chr = str.split(splitline[0], ':')[0]
            strand = splitline[1][0]
            intensity = splitline[4]
            pval = splitline[5]
            motif = splitline[1][1]
            if splitline[1].startswith('+'):
                start = int(str.split(str.split(splitline[0], ':')[1], '-')\
    [0]) + int(splitline[2])
                end = int(str.split(str.split(splitline[0], ':')[1], '-')\
    [0]) + int(splitline[3])
            else:
                start = int(str.split(str.split(splitline[0], ':')[1], '-')\
    [0]) + int(splitline[2])
                end = int(str.split(str.split(splitline[0], ':')[1], '-')\
    [0]) + int(splitline[3])
            outfile.write('%s\t%s\t%s\t%s\t%s\t%s\t%s\n'%(chr, start, end, \
    intensity, pval, strand, motif))
    outfile.close()
    return

def main(argv):
    try:
        opts, args = getopt.getopt(argv, "hi:o:", ["help", "input=", "out="\
    ])
    except getopt.GetoptError, err:
        print str(err)
        sys.exit(2)
    name = False
    outname = False
    for opt, arg in opts:
        if opt in ('-i', '--input'):
            name = arg
        elif opt in ('-h', '--help'):
            print 'python MAST_bed_conversion.py -i cFos_mast_hotspots.txt'
            sys.exit()
    if name:
```

```
        matrix(name)
if __name__ == "__main__":
    main(sys.argv[1:])
```

E Python script: MEME matrix wrapper

```
#! /usr/bin/python
import sys
import getopt
def matrix(filename, outfilename_prefix):
    infile=open(filename, 'r')
    nmotifs = 0
    outfile=open(str(outfilename_prefix) + '_meme.txt', 'w')
    while 1:
        line = infile.readline()
        if not line: break
        splitline = line.split()
        directory = filename.split('meme.txt')[0]
        count = 0
        if line.startswith('MEME version'):
            outfile.write(line)
            outfile.write('\n')
        if line.startswith('ALPHABET'):
            outfile.write(line)
            outfile.write('\n')
        if line.startswith('strands:'):
            outfile.write(line)
            outfile.write('\n')
        if line.startswith('Background letter frequencies'):
            outfile.write(line)
            outfile.write(infile.readline())
            outfile.write('\n')
        if line.startswith('letter-probability matrix:'):
            nmotifs += 1
            outfile.write('%s%s%s\n'%('MOTIF ', outfilename_prefix, '_' + \
    str(nmotifs)))
            outfile.write(line)
            motif_len = splitline[5]
            while 1:
                next = infile.readline()
                if len(next.split()) != 4: break
                outfile.write(next)
            outfile.write('\n')
    outfile.close()
    return

def main(argv):
    try:
        opts, args = getopt.getopt(argv, "hi:o:", ["help", "input=", "out="\
    ])
    except getopt.GetoptError, err:
        print str(err)
        sys.exit(2)
    name = False
    outname = False
    for opt, arg in opts:
        if opt in ('-i', '--input'):
            name = arg
```

```
        if opt in ('-o', '--out'):
            outname = arg
        elif opt in ('-h', '--help'):
            print '~/pyscripts/MEME_matrix.py -i meme.txt -o outfileprefix'
            sys.exit()
    if name and outname:
        matrix(name, outname)
if __name__ == "__main__":
    main(sys.argv[1:])
```

F Python script: production of individual motif meme-format files form the motif database

```
#! /usr/bin/python
import sys
import getopt
def matrix(filename):
    infile=open(filename, 'r')
    while 1:
        line = infile.readline()
        if not line: break
        splitline = line.split()
        count = 0
        if line.startswith('MEME version'):
            meme_line = line
        if line.startswith('ALPHABET'):
            alphabet_line = line
        if line.startswith('strands:'):
            strands_line = line
        if line.startswith('A 0.'):
            bkg_freq = line
        if line.startswith('MOTIF'):
            outfilename_prefix = splitline[1]
            outfile=open(str(outfilename_prefix) + '_meme.txt', 'w')
            outfile.write(meme_line)
            outfile.write('\n')
            outfile.write(alphabet_line)
            outfile.write('\n')
            outfile.write(strands_line)
            outfile.write('\n')
            outfile.write(bkg_freq)
            outfile.write('\n')
            outfile.write(line)
        if line.startswith('letter-probability matrix:'):# or 'log-odds \
   matrix'):
            outfile.write(line)
            while 1:
                next = infile.readline()
                if len(next.split()) != 4: break
                outfile.write(next)
            outfile.write('\n')
            outfile.close()
    return

def main(argv):
    try:
        opts, args = getopt.getopt(argv, "hi:", ["help", "input="])
```

```
    except getopt.GetoptError, err:
        print str(err)
        sys.exit(2)
    name = False
    for opt, arg in opts:
        if opt in ('-i', '--input'):
            name = arg
        elif opt in ('-h', '--help'):
            print 'python MEME_individual_from_db.py -i combined_motif_db.\
   txt'
            sys.exit()
    if name:
        matrix(name)
    else:
        print 'python MEME_individual_from_db.py -i combined_motif_db.txt'
if __name__ == "__main__":
    main(sys.argv[1:])
```

G Python script: converting Sam file to Bed file by ARQ

```
#!/usr/bin/env python
# encoding: utf-8
"""
samToBed.py

Created by Aaron Quinlan on 2009-08-27.
Copyright (c) 2009 Aaron R. Quinlan. All rights reserved.


PURPOSE:
    Convert SAM format alignment files to BED format, where each alignment
    will conrrespond to a distnct BED line.

Modified by Ryan May 2010: ignore "@" lines.
"""

import sys
import getopt
import re

help_message = '''

samToBed -s <sam> -t <alignment type>

OPTIONS:
    -s     The SAM file to be converted to BED
    -t     What types of alignments should be reported?
             "all"    all aligned reads will be reported (Default)
             "con"    only concordant pairs will be reported
             "dis"    only discordant pairs will be reported

'''


class Usage(Exception):
    def __init__(self, msg):
        self.msg = msg

```

```
def processSAM(file, alignType):
    """
        Load a SAM file and convert each line to BED format.

        We avoid readlines() in this case, as SAM files can
        be HUGE, and thus loading it into memory could be painful.
    """
    for line in open(file,'r'):
        if line.startswith('@'):
            continue
        samLine = splitLine(line.strip())
        makeBED(samLine, alignType)


def makeBED(samFields, aType):

    samFlag = int(samFields[1])

    # Only create a BED entry if the read was aligned
    if (not (samFlag & 0x0004)):

        chrom = samFields[2]
        start = str(int(samFields[3])-1)
        end = str(int(samFields[3]) + len(samFields[9]) - 1)
        name = samFields[8]
        strand = getStrand(samFlag)

        # Let's use the edit distance as the BED score.
        #editPattern = re.compile('NM\:i\:(\d+)')
        #editDistance = editPattern.findall(samFields[12])

        # Write the BED line per user's request.
        printBED(aType, properPairing(samFlag),
                chrom, start, end, name, '1', strand)


def splitLine(line, delim="\t"):
    splitline = line.split(delim)
    return splitline


def properPairing(samFlag):
    return samFlag & (0x0002)


def getStrand(samFlag):
    strand = "+"
    if (samFlag & (0x0010)):    # minus strand if true.
        strand = "-"
    return strand


def printBED(aType, concordant, chrom, start, end, name, score, strand):
    try:
        if (aType == "all"):
            print chrom + "\t" + start + "\t" + end + "\t" + name + "\t" + \
    score + "\t" + strand
        else:
```

```
            if (aType == "con") and concordant:
                print chrom + "\t" + start + "\t" + end + "\t" + name + "\t\
    " + score + "\t" + strand
            elif (aType == "dis") and not concordant:
                print chrom + "\t" + start + "\t" + end + "\t" + name + "\t\
    " + score + "\t" + strand
    except IOError, e:
        sys.exit()
    except KeyboardInterrupt, e:
        sys.exit()


def main(argv=None):
    if argv is None:
        argv = sys.argv
    try:
        try:
            opts, args = getopt.getopt(argv[1:], "hs:t:", ["help", "sam", "\
    type"])
        except getopt.error, msg:
            raise Usage(help_message)

        # option processing
        samFile = ""
        aType = "all"
        for option, value in opts:
            if option in ("-h", "--help"):
                raise Usage(help_message)
            if option in ("-s", "--sam"):
                samFile = value
            if option in ("-t", "--type"):
                aType = value


        try:
           f = open(samFile, 'r')
        except IOError, msg:
            raise Usage(help_message)

    except Usage, err:
        print >> sys.stderr, sys.argv[0].split("/")[-1] + ": " + str(err.\
    msg)
        return 2

    # make a BED file of the SAM file.
    processSAM(samFile, aType)

if __name__ == "__main__":
    sys.exit(main())
```

H Python script: fixing the name for Bed file

```
#! /sw/bin/python

import string
import sys
bedFileName=sys.argv[1]
outFileName=sys.argv[2]
```

```
infile=open(bedFileName, 'r')
outfile=open(outFileName, 'w')

while 1:
    line=infile.readline()
    if not line: break
    wrds=string.split(line)
    chrN=wrds[0]
    chrm = str(chrN)
    strand=wrds[5]
    if strand=="+" and int(wrds[3]) > 0:
        pos = int(wrds[1]) + int(wrds[3])
        outfile.write('%s\t%s\t%s\t%s\t%s\t%s\n'%(chrm, wrds[1], str(pos),\
    wrds[3], wrds[4], wrds[5]))
infile.close()
outfile.close()
```

I Token file: parameters (tokens) used by Hotspot for calling hotspot

```
[script-tokenizer]

#######################################
## Notes:  If duplicate token definitions exist, the last definition in
##         the file will be used. Tokens can use .ini variables and \
    declarations.
##         See http://docs.python.org/library/configparser.html
#######################################

#######################################
# Global tokens (used by most scripts)
#######################################

## Tags file in bam format (file extension .bam), or starched bed file
## (file extension .bed.starch).  If the tags file is in bed.starch
## format, and if it is in directory specified by _OUTDIR_, then it
## needs to be in the format
##
##     chr  5'start  5'start+1
##
## That is, the file should be three column bed (no ID field, etc.)
## containing the 1bp coordinates of the 5' ends of the tags. If
## _TAGS_ is a bam file, or a bed.starch file not in _OUTDIR_, then
## the bed.starch file in the above format will be generated (using
## the strand column if present), and put in _OUTDIR_.  NOTE: if you
## use run_badspot, then you must use bam files, or a bed.starch file
## not in _OUTDIR_.

_TAGS_ = MCF10a_merged.rmdup.bam

## For ChIP data with an Input bam file, set _USE_INPUT_ to T, and set
## _INPUT_TAGS_ to the name of that bam file.

_USE_INPUT_ = F
_INPUT_TAGS_ =
```

```
## Genome
_GENOME_ = hg38
## Tag length
_K_ = 75
## Chromosome coordinates, bed format.
_CHROM_FILE_ = /home/ninadmw/Documents/hotspot_052516/hg38.chromInfo.bed
## Location of uniquely mappable positions in the genome for this tag \
   length.
_MAPPABLE_FILE_ = /home/ninadmw/Documents/hotspot_052516/hg38.K76.\
   mappable_only.starch

## Set DUPOK to T for DNaseI data, F for ChIP-seq data (DUPOK = T means \
   allow duplicate reads)
_DUPOK_ = T

## FDR levels, separated by spaces if more than one. Set to N if you
## do not want FDR thresholding (for example, if you just want SPOT
## score computed.)
## _FDRS_ = "N"
_FDRS_ = "0.01"

## Tag density, 150bp window, sliding every 20bp, used for
## peak-finding.  Will be generated, based on the _TAGS_ file, if it
## does not exist. Assumed to be starched bed file, extension
## bed.starch.  Can be blank, in which case the density file will be
## generated in _OUTDIR_, and the the name will be the name of the
## tags file, minus the bam or bed.starch extension, with the added
## extension tagdensity.bed.starch.
_DENS_:

## Output directories (can all be the same location).  Use full path names.
## _OUTDIR_ contains tags files in converted bed.starch and lib.txt formats\
    (for hotspot
## program), and hotspot and peak results.
## _RANDIR_ contains generated random tags (for FDR thresholding) and \
   hotspots called on random tags.
_OUTDIR_ = /home/ninadmw/Documents/hotspot_052516/hs_out_052516
_RANDIR_ = /home/ninadmw/Documents/hotspot_052516/hs_out_052516

## If there are any regions from which tags should be automatically
## omitted, include those here (only if you use run_badspot). May be
## left blank.
_OMIT_REGIONS_: /home/ninadmw/Documents/hotspot_052516/Satellite.hg38.bed

## Set to T if you want scripts to skip steps that have already been done.
_CHECK_ = T

## If _CHECK_ = T, outputs are checked for completeness by searching
## for results for the following chromsome.
_CHKCHR_ = chrX

## Hotspot program binary
_HOTSPOT_ = /home/ninadmw/Documents/programs/hotspot-master/hotspot-distr/\
   hotspot-deploy/bin/hotspot

## Clean up. Remove all intermediate files and directories if set to T.  \
   See
## pipeline script run_final.
_CLEAN_ = T
```

```
## Peak-finding program.
_PKFIND_BIN_ = /home/ninadmw/Documents/programs/hotspot-master/hotspot-\
    distr/hotspot-deploy/bin/wavePeaks
## Peak-finding smoothing level. If the resolution of the input file
## is x, then the results are smoothed out to a scale of (2^level)*x.
_PKFIND_SMTH_LVL_ = 3

## Random number seed, used for generating random tags for FDR thresholding\
    .
_SEED_=101

## Hotspot program parameters
_THRESH_ = 2
_WIN_MIN_ = 200
_WIN_MAX_ = 300
_WIN_INCR_ = 50
_BACKGRD_WIN_ = 50000
_MERGE_DIST_ = 150
_MINSIZE_ = 10
```

J Hotspot executable file: running Hotspot program in the terminal

```
#! /bin/bash

scriptTokBin=/home/ninadmw/Documents/programs/hotspot-master/hotspot-distr/\
    ScriptTokenizer/src/script-tokenizer.py
pipeDir=/home/ninadmw/Documents/programs/hotspot-master/hotspot-distr/\
    pipeline-scripts
tokenFile=MCF10a_merged.tokens.txt

## Do SPOT only (set _FDRS_ to "N" in runall.tokens.txt)
# scripts="$pipeDir/run_make_lib
#     $pipeDir/run_10kb_counts
#     $pipeDir/run_pass1_hotspot
#     $pipeDir/run_pass1_merge_and_thresh_hotspots
#     $pipeDir/run_pass2_hotspot
#     $pipeDir/run_rescore_hotspot_passes
#     $pipeDir/run_spot"

## Do everything, including badspots and final cleanup
scripts="$pipeDir/run_badspot
    $pipeDir/run_make_lib
    $pipeDir/run_wavelet_peak_finding
    $pipeDir/run_10kb_counts
    $pipeDir/run_generate_random_lib
    $pipeDir/run_pass1_hotspot
    $pipeDir/run_pass1_merge_and_thresh_hotspots
    $pipeDir/run_pass2_hotspot
    $pipeDir/run_rescore_hotspot_passes
    $pipeDir/run_spot
    $pipeDir/run_thresh_hot.R
    $pipeDir/run_both-passes_merge_and_thresh_hotspots
    $pipeDir/run_add_peaks_per_hotspot
    $pipeDir/run_final"
```

```
$scriptTokBin \
    --clobber \
    --output-dir=`pwd` \
    $tokenFile \
    $scripts

for script in $scripts
do
    ./$(basename $script).tok
done
```

K Python script: converting Sam file to Bed file

```
#! /sw/bin/python

import string
import sys
samFileName=sys.argv[1]
bedFileName=sys.argv[2]

infile=open(samFileName, 'r')
outfile=open(bedFileName, 'w')
strandByFlag={'0':'+','16':'-'}
strandRGB={'+':'0,0,0','-':'0,0,0'}
#chr="chr"
commentChars=["@"]
goodFlags=["0","16"]

IUPACtoNucleotides = {}
IUPACtoNucleotides['R'] = '[AG]'
IUPACtoNucleotides['Y'] = '[CT]'
IUPACtoNucleotides['S'] = '[GC]'
IUPACtoNucleotides['W'] = '[AT]'
IUPACtoNucleotides['K'] = '[GT]'
IUPACtoNucleotides['M'] = '[AC]'
IUPACtoNucleotides['B'] = '[CGT]'
IUPACtoNucleotides['D'] = '[AGT]'
IUPACtoNucleotides['H'] = '[ACT]'
IUPACtoNucleotides['V'] = '[ACG]'
IUPACtoNucleotides['N'] = '[CGTA]'

complementTable = string.maketrans('ACTGRYSWKMBDHVNactgryswkmbdhvn','\
   TGACYRSWMKVHDBNtgacyrswmkvhdbn')

def makeRC(origSeq):
    seqList = list(origSeq)
    seqList.reverse()
    seqRC = string.join(seqList,'')
    seqRC = seqRC.translate(complementTable)
    return seqRC

while 1:
        line=infile.readline()
        if not line: break
        if line[0] in commentChars:
                continue
        wrds=string.split(line)
        if wrds[1] not in goodFlags:
```

```
                continue
        sequence=wrds[9]
        chrN=wrds[2]
        chrm = str(chrN)
        one=1
        strand=strandByFlag[wrds[1]]
        color=strandRGB[strand]
        psn=int(wrds[3])-1
        if strand=="-":
                psn+=len(wrds[9])
                sequence=makeRC(sequence)
        outfile.write('%s\t%s\t%s\t%s\t%s\t%s\t%s\t%s\t%s\n'%(chrm, psn, \
    psn+1, sequence, one, strand, psn, psn+1, color))
infile.close()
outfile.close()
```

L R script: production of composite plots for DNase-seq and conservation data with MAST output files

```
#libraries
library(lattice)
library(bigWig)
library(latticeExtra)

#functions
composites.test <- function(path.dir, composite.input, region=20, step=1, \
    grp = 'DNase') {
  vec.names = c('chr','start','end')
  hmap.data = list()
  composite.df=data.frame(matrix(ncol = 6, nrow = 0))
  for (mod.bigWig in Sys.glob(file.path(path.dir, "*.bigWig"))) {
    factor.name = strsplit(strsplit(mod.bigWig, "/")[[1]][length(strsplit(\
    mod.bigWig, "/")[[1]])], '\\.')[[1]][1]
    print(factor.name)
    vec.names = c(vec.names, factor.name)
    wiggle = load.bigWig(mod.bigWig)
    bpquery = window.step(composite.input, wiggle, region, step)
    mult.row = ncol(bpquery[[1]])
    hmap.data[[factor.name]] = bpquery[[1]]
    df.up <- data.frame(matrix(ncol = 6, nrow = mult.row))
    df.up[, 1] <- colMeans(bpquery[[1]])
    df.up[, 2] <- seq((-1 * region) + 0.5 * step, region - 0.5 * step, by =\
     step)
    df.up[, 3] <- matrix(data = factor.name, nrow=mult.row, ncol=1)
    df.up[, 4] <- df.up[, 1]
    df.up[, 5] <- df.up[, 1]
    df.up[, 6] <- matrix(data = grp, nrow=mult.row, ncol=1)
    composite.df = rbind(composite.df, df.up)
    unload.bigWig(wiggle)
  }

  colnames(composite.df) <- c('est', 'x', 'cond', 'upper', 'lower', 'grp')
  composite.df = composite.df[composite.df[,2] >= -1000 & composite.df[,2] \
   <= 1000,]
  for (cond in (1:length(hmap.data))) {
    rownames(hmap.data[[cond]]) = paste(composite.input[,1], ':', composite\
    .input[,2], '-', composite.input[,3], sep='')
```

```
    colnames(hmap.data[[cond]]) = seq((-1 * region) + 0.5 * step, region - \
    0.5 * step, by = step)
  }
  return(list(composite.df, hmap.data))
}

processes.comps <- function(fimo = 'mast_hotspots_r4_FOSL2.txt',
                            fimobed='mast_hotspots_r4_FOSL2.bed',
                            hotspots = 'Hotspots_Either_E2_condition_merged\
    .bed',
                            path.dir = 'UW_DNase', region = 200) {
  factor.name = strsplit(strsplit(fimo, "/mast_")[[1]][length(strsplit(fimo\
    , "/mast_")[[1]])], '\\.txt')[[1]][1]
  print(factor.name)
  all.fimo.bed = parse.mast(fimobed)
  print('rows in original bed')
  print(nrow(all.fimo.bed))
  all.fimo.bed = all.fimo.bed[all.fimo.bed[,2] > region,]#remove rows off \
    teh end of chrromosome

  chrom.bW = load.bigWig(Sys.glob(file.path(path.dir, "*.bigWig"))[1])
  for (i in 1:length(chrom.bW$chroms)) {
    all.fimo.bed = subset(all.fimo.bed, (all.fimo.bed[,1] == chrom.bW$\
    chroms[i]) & (all.fimo.bed[,3] < chrom.bW$chromSizes[i]) | all.fimo.bed\
    [,1] != chrom.bW$chroms[i])
  }
  print('rows in bed after filtering coordiates that reside off the \
    chromosome boundaries')
  print(nrow(all.fimo.bed))
  hotspots = read.table(hotspots, header=F)
  fimo.overlap.hotspots = bedTools.intersect(bed1 = hotspots, bed2=all.fimo\
    .bed, opt.string ='-wb')
  fimo.composites = composites.test(path.dir, fimo.overlap.hotspots, region\
     = region, grp = factor.name)
  return(fimo.composites)
}

parse.mast <- function(file) {
  mast.data = read.table(file, colClasses=c('character','integer','integer'\
    ,'numeric', 'numeric','character','character'))
  res = mast.data
  colnames(res) = c('chr', 'start', 'end', 'score', 'pval', 'strand', '\
    motif')
  return(res)
}


window.step <- function(bed, bigWig, halfWindow, step) {
  windowSize = (2*halfWindow) %/% step
  midPoint = floor((as.numeric(as.character(bed[,2])) + as.numeric(as.\
    character(bed[,3]))) / 2)
  start = (midPoint - halfWindow)
  end = start + windowSize*step
  if ((as.numeric(as.character(bed[1,2])) + as.numeric(as.character(bed\
    [1,3]))) %% 2 == 0) {
    bed[,2] = start
    bed[,3] = end
  } else {
    bed[,2] = start
```

```
    bed[,3] = end
    bed[,2][bed[,6] == '-'] = bed[,2][bed[,6] == '-'] + 1
    bed[,3][bed[,6] == '-'] = bed[,3][bed[,6] == '-'] + 1
  }
  matrix.comp = bed6.step.bpQuery.bigWig(bigWig, bigWig, bed, 1, op = "avg"\
    , follow.strand = TRUE)
  res = do.call(rbind, matrix.comp)
  return(list(res, matrix.comp))
}

bedTools.intersect<-function(functionstring="/usr/local/bin/bedtools2/\
    intersectBed",bed1,bed2,opt.string="") {
  #create temp files
  a.file=tempfile()
  b.file=tempfile()
  out   =tempfile()
  options(scipen =99) # not to use scientific notation when writing out

  #write bed formatted dataframes to tempfile
  write.table(bed1,file=a.file,quote=F,sep="\t",col.names=F,row.names=F)
  write.table(bed2,file=b.file,quote=F,sep="\t",col.names=F,row.names=F)

  # create the command string and call the command using system()
  command=paste(functionstring, opt.string,"-a",a.file,"-b",b.file,">",out,\
    sep=" ")
  cat(command,"\n")
  try(system(command))

  res=read.table(out,header=F, comment.char='')
  unlink(a.file);unlink(b.file);unlink(out)
  return(res[,c(4,5,6,7,8,9)])
}

composites.func.panels.naked.chromatin <- function(dat, fact = 'Factor', \
    summit = 'Summit', num=90) {
  col.lines = c(rgb(0,0,1,1/2), rgb(1,0,0,1/2), "purple", "#0faa12")
  count = length(unique(dat$grp))
  pdf(paste('composite_', fact, '_signals_', summit, '_peaks.pdf', sep=''),\
     width=2* 3.43, height=(count/2) * 3.43)
  print(xyplot(est ~ x|grp, group = cond, data = dat,
               type = 'l',
               scales=list(x=list(cex=0.8, at=seq(-(num),(num),(num)/3),\
    relation = "free"), y =list(cex=0.8, relation="free")),
               xlim=c(-(num),(num)),
               col = col.lines,
               auto.key = list(points=F, lines=T, cex=0.8),
               par.settings = list(superpose.symbol = list(pch = c(16), col\
    =col.lines), superpose.line = list(col = col.lines, lwd=3)),
               cex.axis=1.0,
               par.strip.text=list(cex=0.9, font=1, col='black'),
               aspect=1.0,
               between=list(y=0.5, x=0.5),
               lwd=3,
               ylab = list(label = paste(fact," Cut Frequency", sep=''), \
    cex =0.8),
               xlab = list(label = paste("Distance from ", summit, " center\
    ",sep=''), cex =0.8),
               upper = dat$upper,
               lower = dat$lower,
```

```
               strip = function(..., which.panel, bg) {
                 bg.col = c("grey85")# "grey40","#0a823c", "#0a823c") #blue\
   , red green
                 strip.default(..., which.panel = which.panel, bg = rep(bg.\
   col, length = which.panel)[which.panel])
               }
  ))
  dev.off()
}


composites.func.panels.conservation.x <- function(dat, fact = '', summit = \
    'Summit', num=20) {
  col.lines = c(rgb(0,0,1,1/2), rgb(1,0,0,1/2))
  count = length(unique(dat$grp))
  cons.dat = dat[dat$cond == 'phastCon',]
  phylo.dat = dat[dat$cond == 'phyloP',]

  pdf(paste('composite_', fact, '_conservation_', summit, '_peaks.pdf', sep\
   =''), width=2*3.43, height=(count/2) * 3.43)
  cons = xyplot(est ~ x|grp, group = cond, data = cons.dat,
                type = 'l',
                scales=list(x=list(cex=0.8, at=seq(-(num),(num),(num)/3),\
   relation = "free"), y =list(cex=0.8, relation="free")),
                xlim=c(-(num),(num)),
                col = col.lines,
                key = list(lines=list(col=col.lines, lwd=3), text = list(c(\
   'phastCons','phyloP')), cex=0.8),
                par.settings = list(superpose.symbol = list(pch = c(16), \
   col='black'), superpose.line = list(col = rgb(0,0,1,1/2), lwd=1)),
                cex.axis=1.0,
                par.strip.text=list(cex=0.9, font=1, col='black'),
                aspect=1.0,
                between=list(y=0.5, x=5.5),
                lwd=3,
                ylab = list(label = paste(fact,"Average Conservation Score"\
   , sep=''), cex =0.8),
                xlab = list(label = paste("Distance from ", summit, " \
   center",sep=''), cex =0.8),
                upper = dat$upper,
                lower = dat$lower,
                strip = function(..., which.panel, bg) {
                  bg.col = c("grey85")# "grey40","#0a823c", "#0a823c") #\
   blue, red green
                  strip.default(..., which.panel = which.panel, bg = rep(bg\
   .col, length = which.panel)[which.panel])
                }
  )
  phylo = xyplot(est ~ x|grp, group = cond, data = phylo.dat,
                 type = 'l',
                 scales=list(x=list(cex=0.8, at=seq(-(num),(num),(num)/3),\
   relation = "free"), y =list(cex=0.8, relation="free")),
                 xlim=c(-(num),(num)),
                 col = rev(col.lines),
                 par.settings = list(superpose.symbol = list(pch = c(16), \
   col='black'), superpose.line = list(col = rgb(1,0,0,1/2), lwd=1)),
                 cex.axis=1.0,
                 par.strip.text=list(cex=0.9, font=1, col='black'),
                 aspect=1.0,
```

```
                  between=list(y=0.5, x=5.5),
                  lwd=3,
                  ylab = list(label = paste(fact,"Average Conservation Score\
    ", sep=''), cex =0.8),
                  xlab = list(label = paste("Distance from ", summit, " \
    center",sep=''), cex =0.8),
                  upper = dat$upper,
                  lower = dat$lower,
                  strip = function(..., which.panel, bg) {
                    bg.col = c("grey85")# "grey40","#0a823c", "#0a823c") #\
    blue, red green
                    strip.default(..., which.panel = which.panel, bg = rep(\
    bg.col, length = which.panel)[which.panel])
                  }
  )
  print(doubleYScale(phylo, cons))
  dev.off()
}

change.format <- function(process.pipe.output, min = -6, max = 6) {
  process.pipe.output = process.pipe.output[[1]]
  process.pipe.output$cond[process.pipe.output$cond == 'UW_MCF7_both'] = '\
    Chromatin'
  process.pipe.output$cond[process.pipe.output$cond == 'IMR90_Naked_DNase']\
     = 'Naked DNA'
  all.ctcf.norm = norm.com.df(process.pipe.output[process.pipe.output$cond \
    == 'Naked DNA',], process.pipe.output[process.pipe.output$cond == '\
    Chromatin' & process.pipe.output$x >= min & process.pipe.output$x <= max\
    ,])
  w = rbind(process.pipe.output[process.pipe.output$cond == 'Chromatin',], \
    all.ctcf.norm)
  return(w)
}

norm.com.df <- function(comp.df, norm.to) {
  min.norm = min(norm.to$est)
  min.comp = min(comp.df$est[comp.df$est!=0])
  fact = min.norm/min.comp
  comp.df$est = comp.df$est*fact
  zeroed.df = comp.df$est - min(comp.df$est[comp.df$est!=0])
  zeroed.df = zeroed.df / max(zeroed.df)
  max.norm.minus.min.comp = max(norm.to$est) - min(comp.df$est[comp.df$est!\
    =0])
  new = min(comp.df$est[comp.df$est!=0]) + zeroed.df * max.norm.minus.min.\
    comp
  comp.df$est = new
  return(comp.df)
}


cycle.fimo <- function(path.dir.fimo = '~/mast_file_for_conservation', path\
    .dir.bigWig = '~/UW_DNase_MCF7', hotspot.file = 'Hotspots_Either_E2_\
    condition.merge.broadPeak', window = 200) {
  composite.df=data.frame(matrix(ncol = 6, nrow = 0))
  for (fimo.file in Sys.glob(file.path(path.dir.fimo, "*mast_output/*.txt")\
    )) {
     factor.name = strsplit(strsplit(fimo.file, "mast_")[[1]][length(\
    strsplit(fimo.file, "mast_")[[1]])], '\\.')[[1]][1]
     print(factor.name)
```

```
    tf.x = processes.comps(fimo = fimo.file, fimobed=paste0(strsplit(fimo.\
   file,'.txt'),'.bed'),
                               hotspots = hotspot.file, path.dir =  path.dir.\
   bigWig, region = window)
    plot.composite.fimo = change.format(tf.x, -10, 10)#10 is hardcoded, it \
   may vary per fimo output
    composite.df = rbind(composite.df, plot.composite.fimo)
  }
  composites.func.panels.naked.chromatin(composite.df, 'DNase', 'Motif', \
   num =100)#this is hard coded and shouldn't be
  return(composite.df)
}

cycle.fimo.conservation <- function(path.dir.fimo = '~/mast_file_for_\
   conservation', path.dir.bigWig = '~/UW_DNase_MCF7', hotspot.file = '\
   iterative/Hotspots_Either_E2_condition.merge.broadPeak', window = 200) {
  composite.df=data.frame(matrix(ncol = 6, nrow = 0))
  for (fimo.file in Sys.glob(file.path(path.dir.fimo, "*mast_output/*.txt")\
   )) {
    factor.name = strsplit(strsplit(fimo.file, "mast_")[[1]][length(\
   strsplit(fimo.file, "mast_")[[1]])], '\\.')[[1]][1]
    print(factor.name)
    tf.x = processes.comps(fimo = fimo.file, fimobed=paste0(strsplit(fimo.\
   file,'.txt'),'.bed'),
                               hotspots = hotspot.file, path.dir =  path.dir.\
   bigWig, region = window)
    print(head(tf.x[[1]]))
    composite.df = rbind(composite.df, tf.x[[1]])
  }
  composites.func.panels.conservation.x(composite.df, summit = 'Motif', num\
    =100)
  return(composite.df)
}

#processes

all.composites.dnase = cycle.fimo(path.dir.fimo = '~/mast_file_for_\
   conservation', path.dir.bigWig = '~/UW_DNase_MCF7', hotspot.file = '\
   Hotspots_Either_E2_condition.merge.broadPeak', window = 200)
all.composites.conservation = cycle.fimo.conservation(path.dir.bigWig = '~/\
   conservation_files')
```

M Python script: identification of motif families

```
#! /usr/bin/python
import sys
import getopt
from collections import defaultdict

def list_tuples(filename):
    infile=open(filename, 'r')
    lst = []
    while 1:
        line = infile.readline()
        if not line: break
        splitline = line.split()
        if (len(splitline) == 1 | len(splitline) == 0):
            continue
```

```
        else:
            mytuple = ()
            lengthline = len(splitline)
            for i in splitline:
                mytuple = mytuple + (i,)
            lst.append(mytuple)
            #print mytuple
    list_of_lists = [list(elem) for elem in lst]
    return list_of_lists

def union(lis):
  lis = [set(e) for e in lis]
  res = []
  while True:
    for i in range(len(lis)):
      a = lis[i]
      if res == []:
        res.append(a)
      else:
        pointer = 0
        while pointer < len(res):
          if a & res[pointer] != set([]) :
            res[pointer] = res[pointer].union(a)
            break
          pointer +=1
        if pointer == len(res):
          res.append(a)
    if res == lis:
      break
    lis,res = res,[]
  return res

def main(argv):
    try:
        opts, args = getopt.getopt(argv, "hi:", ["help", "input="])
    except getopt.GetoptError, err:
        print str(err)
        sys.exit(2)
    name = False
    for opt, arg in opts:
        if opt in ('-i', '--input'):
            name = arg
        elif opt in ('-h', '--help'):
            print 'python ~/pyscripts/TOMTOM_motif_families_nonrecursive.py\
     -i maplist_subset.txt'
            sys.exit()
    if name:
        lst = list_tuples(name)
        #set_lst =[set(i) for i in lst if i]
        families = union(lst)
        outfile = open(str.split(name, '.')[0] + '_families.txt','w')
        for i in families:
            for item in i:
                outfile.write("%s\t" % item)
            outfile.write("\n")
        outfile.close()
    else:
        print 'python ~/pyscripts/TOMTOM_motif_families_nonrecursive.py  -i\
     maplist_subset.txt'
```

```
if __name__ == "__main__":
    main(sys.argv[1:])
```

N R script: production of composite plots for DNase-seq and conservation data with orphan MAST output files

```
#libraries
library(lattice)
library(bigWig)
library(latticeExtra)

#functions
composites.test <- function(path.dir, composite.input, region=20, step=1, \
    grp = 'DNase') {
  vec.names = c('chr','start','end')
  hmap.data = list()
  composite.df=data.frame(matrix(ncol = 6, nrow = 0))
  for (mod.bigWig in Sys.glob(file.path(path.dir, "*.bigWig"))) {
    factor.name = strsplit(strsplit(mod.bigWig, "/")[[1]][length(strsplit(\
   mod.bigWig, "/")[[1]])], '\\.')[[1]][1]
    print(factor.name)
    vec.names = c(vec.names, factor.name)
    wiggle = load.bigWig(mod.bigWig)
    bpquery = window.step(composite.input, wiggle, region, step)
    subsample = subsampled.quantiles.metaprofile((bpquery[[1]]))#\
   alternative functions: bootstrapped.confinterval.metaprofile, \
   confinterval.metaprofile, subsampled.quantiles.metaprofile
    mult.row = ncol(bpquery[[1]])
    hmap.data[[factor.name]] = bpquery[[1]]
    df.up <- data.frame(matrix(ncol = 6, nrow = mult.row))
    df.up[, 1] <- subsample$middle
    df.up[, 2] <- seq((-1 * region) + 0.5 * step, region - 0.5 * step, by =\
     step)
    df.up[, 3] <- matrix(data = factor.name, nrow=mult.row, ncol=1)
    df.up[, 4] <- subsample$top
    df.up[, 5] <- subsample$bottom
    df.up[, 6] <- matrix(data = grp, nrow=mult.row, ncol=1)
    composite.df = rbind(composite.df, df.up)
    unload.bigWig(wiggle)
  }

  colnames(composite.df) <- c('est', 'x', 'cond', 'upper', 'lower', 'grp')
  composite.df = composite.df[composite.df[,2] >= -1000 & composite.df[,2] \
    <= 1000,]
  for (cond in (1:length(hmap.data))) {
    rownames(hmap.data[[cond]]) = paste(composite.input[,1], ':', composite\
   .input[,2], '-', composite.input[,3], sep='')
    colnames(hmap.data[[cond]]) = seq((-1 * region) + 0.5 * step, region - \
   0.5 * step, by = step)
  }
  return(list(composite.df, hmap.data))
}

processes.comps <- function(fimo = 'mast_hotspots_r4_FOSL2.txt',
                            fimobed='mast_hotspots_r4_FOSL2.bed',
                            hotspots = 'Hotspots_Either_E2_condition_merged\
   .bed',
```

```
                              path.dir = 'UW_DNase', region = 200)
  {
  factor.name = strsplit(strsplit(fimo, "/mast_")[[1]][length(strsplit(fimo\
    , "/mast_")[[1]])], '\\.txt')[[1]][1]
  print(factor.name)
  all.fimo.bed = parse.mast(fimobed)
  print('rows in original bed')
  print(nrow(all.fimo.bed))
  all.fimo.bed = all.fimo.bed[all.fimo.bed[,2] > region,]#remove rows off \
    teh end of chrromosome

  chrom.bW = load.bigWig(Sys.glob(file.path(path.dir, "*.bigWig"))[1])
  for (i in 1:length(chrom.bW$chroms)) {
     all.fimo.bed = subset(all.fimo.bed, (all.fimo.bed[,1] == chrom.bW$\
    chroms[i]) & (all.fimo.bed[,3] < chrom.bW$chromSizes[i]) | all.fimo.bed\
    [,1] != chrom.bW$chroms[i])
  }
  print('rows in bed after filtering coordiates that reside off the \
    chromosome boundaries')
  print(nrow(all.fimo.bed))
  hotspots = read.table(hotspots, header=F)
  fimo.overlap.hotspots = bedTools.intersect(bed1 = hotspots, bed2=all.fimo\
    .bed, opt.string ='-wb')
  fimo.composites = composites.test(path.dir, fimo.overlap.hotspots, region\
     = region, grp = factor.name)
  return(fimo.composites)
}


parse.mast <- function(file) {
  mast.data = read.table(file, colClasses=c('character','integer','integer'\
    ,'numeric', 'numeric','character','character'))
  res = mast.data
  colnames(res) = c('chr', 'start', 'end', 'score', 'pval', 'strand', '\
    motif')
  return(res)
}


window.step <- function(bed, bigWig, halfWindow, step) {
  windowSize = (2*halfWindow) %/% step
  midPoint = floor((as.numeric(as.character(bed[,2])) + as.numeric(as.\
    character(bed[,3]))) / 2)
  start = (midPoint - halfWindow)
  end = start + windowSize*step
  if ((as.numeric(as.character(bed[1,2])) + as.numeric(as.character(bed\
    [1,3]))) %% 2 == 0) {
     bed[,2] = start
     bed[,3] = end
  } else {
     bed[,2] = start
     bed[,3] = end
     bed[,2][bed[,6] == '-'] = bed[,2][bed[,6] == '-'] + 1
     bed[,3][bed[,6] == '-'] = bed[,3][bed[,6] == '-'] + 1
  }
  matrix.comp = bed6.step.bpQuery.bigWig(bigWig, bigWig, bed, 1, op = "avg"\
    , follow.strand = TRUE)
  res = do.call(rbind, matrix.comp)
  return(list(res, matrix.comp))
```

```
}

bedTools.intersect<-function(functionstring="/usr/local/bin/bedtools2/\
   intersectBed",bed1,bed2,opt.string="") {
  #create temp files
  a.file=tempfile()
  b.file=tempfile()
  out   =tempfile()
  options(scipen =99) # not to use scientific notation when writing out

  #write bed formatted dataframes to tempfile
  write.table(bed1,file=a.file,quote=F,sep="\t",col.names=F,row.names=F)
  write.table(bed2,file=b.file,quote=F,sep="\t",col.names=F,row.names=F)

  # create the command string and call the command using system()
  command=paste(functionstring, opt.string,"-a",a.file,"-b",b.file,">",out,\
   sep=" ")
  cat(command,"\n")
  try(system(command))

  res=read.table(out,header=F, comment.char='')
  unlink(a.file);unlink(b.file);unlink(out)
  return(res[,c(4,5,6,7,8,9)])
}


my.panel.bands <-
    function(x, y, upper, lower,
             fill, col,
             subscripts, ..., font, fontface)
{
    upper <- upper[subscripts]
    lower <- lower[subscripts]
    panel.polygon(c(x, rev(x)), c(upper, rev(lower)),
                  col = fill, border = FALSE,
                  ...)
}

composites.func.panels.naked.chromatin <- function(dat, fact = 'Factor', \
   summit = 'Summit', num=90) {
  col.lines = c(rgb(0,0,1,1/2), rgb(1,0,0,1/2), "purple", "#0faa12")
  count = length(unique(dat$grp))
  pdf(paste('composite_', fact, '_signals_', summit, '_peaks.pdf', sep=''),\
    width=2* 3.43, height=ceiling((count/2)) * 3.43)
  print(xyplot(est ~ x|grp, group = cond, data = dat,
               type = 'l',
               scales=list(x=list(cex=0.8,relation = "free"), y =list(cex\
   =0.8, relation="free")),
               xlim=c(-(num),(num)),
               col = col.lines,
               auto.key = list(points=F, lines=T, cex=0.8),
               par.settings = list(superpose.symbol = list(pch = c(16), col\
   =col.lines), superpose.line = list(col = col.lines, lwd=3)),
               cex.axis=1.0,
               par.strip.text=list(cex=0.9, font=1, col='black'),
               aspect=1.0,
               between=list(y=0.5, x=0.5),
               lwd=3,
               ylab = list(label = paste(fact," Cut Frequency", sep=''), \
```

```
    cex =0.8),
              xlab = list(label = paste("Distance from ", summit, " center\
    ",sep=''), cex =0.8),
              upper = dat$upper,
              fill = c(rgb(0,0,1,1/4), rgb(1,0,0,1/4), "purple", "#0faa12"\
    ),
              lower = dat$lower,
              strip = function(..., which.panel, bg) {
                bg.col = c("grey85")# "grey40","#0a823c", "#0a823c") #blue\
    , red green
                strip.default(..., which.panel = which.panel, bg = rep(bg.\
    col, length = which.panel)[which.panel])
            },
              panel = function(x, y, ...){
                  panel.superpose(x, y, panel.groups = 'my.panel.bands', \
    ...)
                  panel.xyplot(x, y, ...)
        }
  ))
  dev.off()
}

composites.func.panels.conservation.x <- function(dat, fact = '', summit = \
    'Summit', num=20) {
  col.lines = c(rgb(0,0,1,2/3), rgb(1,0,0,2/3))
  count = length(unique(dat$grp))
  cons.dat = dat[dat$cond == 'phastCon',]
  phylo.dat = dat[dat$cond == 'phyloP',]
  pdf(paste('composite_', fact, '_conservation_', summit, '_peaks.pdf', sep\
    =''), width=2*3.43, height=(count/2) * 3.43)
  cons = xyplot(est ~ x|grp, group = cond, data = cons.dat,
      type = 'l',
      scales=list(x=list(cex=0.8, relation = "free"), y =list(cex=0.8, \
    relation="free")),
      xlim=c(-(num),(num)),
      col = col.lines,
      key = list(lines=list(col=col.lines, lwd=3), text = list(c('phastCons\
    ','phyloP')), cex=0.8),
      par.settings = list(superpose.symbol = list(pch = c(16), col='black')\
    , superpose.line = list(col = rgb(0,0,1,1/2), lwd=1)),
      cex.axis=1.0,
      par.strip.text=list(cex=0.9, font=1, col='black'),
      aspect=1.0,
      fill = c(rgb(0,0,1,1/4), rgb(1,0,0,1/4), "purple", "#0faa12"),
      between=list(y=0.5, x=5.5),
      lwd=3,
      ylab = list(label = paste(fact,"Average Conservation Score", sep=''),\
     cex =0.8),
      xlab = list(label = paste("Distance from ", summit, " center",sep='')\
    , cex =0.8),
      upper = cons.dat$upper,
      lower = cons.dat$lower,
      strip = function(..., which.panel, bg) {
          bg.col = c("grey85")# "grey40","#0a823c", "#0a823c") #blue, red \
    green
          strip.default(..., which.panel = which.panel, bg = rep(bg.col, \
    length = which.panel)[which.panel])
      },
      panel = function(x, y, ...){
```

```
          panel.superpose(x, y, panel.groups = 'my.panel.bands', ...)
          panel.xyplot(x, y, ...)
      }
                )
  phylo = xyplot(est ~ x|grp, group = cond, data = phylo.dat,
      type = 'l',
      scales=list(x=list(cex=0.8, relation = "free"), y =list(cex=0.8, \
   relation="free")),
      xlim=c(-(num),(num)),
      col = rev(col.lines),
      par.settings = list(superpose.symbol = list(pch = c(16), col='black')\
   , superpose.line = list(col = rgb(1,0,0,1/2), lwd=1)),
      cex.axis=1.0,
      par.strip.text=list(cex=0.9, font=1, col='black'),
      aspect=1.0,
      fill = rev(c(rgb(0,0,1,1/4), rgb(1,0,0,1/4))),
      between=list(y=0.5, x=5.5),
      lwd=3,
      ylab = list(label = paste(fact,"Average Conservation Score", sep=''),\
     cex =0.8),
      xlab = list(label = paste("Distance from ", summit, " center",sep='')\
   , cex =0.8),
      upper = phylo.dat$upper,
      lower = phylo.dat$lower,
      strip = function(..., which.panel, bg) {
          bg.col = c("grey85")# "grey40","#0a823c", "#0a823c") #blue, red \
   green
          strip.default(..., which.panel = which.panel, bg = rep(bg.col, \
   length = which.panel)[which.panel])
      },
      panel = function(x, y, ...){
        panel.superpose(x, y, panel.groups = 'my.panel.bands', ...)
        panel.xyplot(x, y, ...)
      }
                 )
  print(doubleYScale(phylo, cons))
  dev.off()
}

change.format <- function(process.pipe.output, min = -6, max = 6) {
  process.pipe.output = process.pipe.output[[1]]
  #process.pipe.output$cond[process.pipe.output$cond == 'UW_MCF7_both'] = '\
   Chromatin'
  #process.pipe.output$cond[process.pipe.output$cond == 'IMR90_Naked_DNase\
   '] = 'Naked DNA'
  process.pipe.output$cond[process.pipe.output$cond == 'MCF7_Chromatin_6-\
   mer'] = 'Chromatin'
  process.pipe.output$cond[process.pipe.output$cond == 'Naked_DNA_6-mer'] =\
    'Naked DNA'
  all.ctcf.norm = norm.com.df(process.pipe.output[process.pipe.output$cond \
   == 'Naked DNA',], process.pipe.output[process.pipe.output$cond == '\
   Chromatin' & process.pipe.output$x >= min & process.pipe.output$x <= max\
   ,])
  w = rbind(process.pipe.output[process.pipe.output$cond == 'Chromatin',], \
   all.ctcf.norm)
  return(w)
}

norm.com.df <- function(comp.df, norm.to) {
```

```
  min.norm = min(norm.to$est)
  min.comp = min(comp.df$est[comp.df$est!=0])
  fact = min.norm/min.comp
  comp.df$est = comp.df$est*fact
  zeroed.df = comp.df$est - min(comp.df$est[comp.df$est!=0])
  zeroed.df = zeroed.df / max(zeroed.df)
  max.norm.minus.min.comp = max(norm.to$est) - min(comp.df$est[comp.df$est!\
    =0])
  new = min(comp.df$est[comp.df$est!=0]) + zeroed.df * max.norm.minus.min.\
    comp
  comp.df$est = new
  min.norm = min(norm.to$upper)
  min.comp = min(comp.df$upper[comp.df$upper!=0])
  fact = min.norm/min.comp
  comp.df$upper = comp.df$upper*fact
  zeroed.df = comp.df$upper - min(comp.df$upper[comp.df$upper!=0])
  zeroed.df = zeroed.df / max(zeroed.df)
  max.norm.minus.min.comp = max(norm.to$upper) - min(comp.df$upper[comp.df$\
    upper!=0])
  new = min(comp.df$upper[comp.df$upper!=0]) + zeroed.df * max.norm.minus.\
    min.comp
  comp.df$upper = new
  min.norm = min(norm.to$lower)
  min.comp = min(comp.df$lower[comp.df$lower!=0])
  fact = min.norm/min.comp
  comp.df$lower = comp.df$lower*fact
  zeroed.df = comp.df$lower - min(comp.df$lower[comp.df$lower!=0])
  zeroed.df = zeroed.df / max(zeroed.df)
  max.norm.minus.min.comp = max(norm.to$lower) - min(comp.df$lower[comp.df$\
    lower!=0])
  new = min(comp.df$lower[comp.df$lower!=0]) + zeroed.df * max.norm.minus.\
    min.comp
  comp.df$lower = new
  return(comp.df)
}

cycle.fimo <- function(path.dir.fimo = '~/mast_file_for_conservation', path\
    .dir.bigWig = '~/UW_DNase_MCF7', hotspot.file = 'Hotspots_Either_E2_\
    condition.merge.broadPeak', window = 200) {
  composite.df=data.frame(matrix(ncol = 6, nrow = 0))
  for (fimo.file in Sys.glob(file.path(path.dir.fimo, "*mast_output/*.txt")\
    )) {
     factor.name = strsplit(strsplit(fimo.file, "mast_")[[1]][length(\
    strsplit(fimo.file, "mast_")[[1]])], '\\.')[[1]][1]
     print(factor.name)
     tf.x = processes.comps(fimo = fimo.file, fimobed=paste0(strsplit(fimo.\
    file,'.txt'),'.bed'),
                               hotspots = hotspot.file, path.dir =  path.dir.\
    bigWig, region = window)
     plot.composite.fimo = change.format(tf.x, -10, 10)#10 is hardcoded, it \
    may vary per fimo output
     composite.df = rbind(composite.df, plot.composite.fimo)
  }
  composites.func.panels.naked.chromatin(composite.df, 'DNase', 'Motif', \
    num =100)#this is hard coded and shouldn't be
  return(composite.df)
}

cycle.fimo.conservation <- function(path.dir.fimo = '~/mast_file_for_\
```

```
    conservation', path.dir.bigWig = '~/UW_DNase_MCF7', hotspot.file = '\
    iterative/Hotspots_Either_E2_condition.merge.broadPeak', window = 200) {
  composite.df=data.frame(matrix(ncol = 6, nrow = 0))
  for (fimo.file in Sys.glob(file.path(path.dir.fimo, "*mast_output/*.txt")\
    )) {
     factor.name = strsplit(strsplit(fimo.file, "mast_")[[1]][length(\
    strsplit(fimo.file, "mast_")[[1]])], '\\.')[[1]][1]
     print(factor.name)
     tf.x = processes.comps(fimo = fimo.file, fimobed=paste0(strsplit(fimo.\
    file,'.txt'),'.bed'),
                               hotspots = hotspot.file, path.dir =  path.dir.\
    bigWig, region = window)
     print(head(tf.x[[1]]))
     print(head(tf.x[[2]]))
     composite.df = rbind(composite.df, tf.x[[1]])
  }
  composites.func.panels.conservation.x(composite.df, summit = 'Motif', num\
     =100)
  return(composite.df)
}

#processes
#for all the non-orphan motifs
orphan=c('~/Desktop/Iterate_MCF7_hotspot_onestop/iteration/mast_files_for_\
    conservation_plots_orphan')
all.composites.dnase =
  cycle.fimo(path.dir.fimo = orphan,
             path.dir.bigWig = '~/Desktop/normalized_bigWig/normalized_\
    bigwig/MCF7',
             hotspot.file = '~/iterative/Hotspots_Either_E2_condition.merge\
    .broadPeak', window = 200)

all.composites.conservation =
  cycle.fimo.conservation(path.dir.fimo = orphan,
                          path.dir.bigWig = '~/conservation_files',
                          hotspot.file = '~/iterative/Hotspots_Either_E2_\
    condition.merge.broadPeak', window = 200)

save(all.composites.dnase,all.composites.conservation,file="MCF7_\
    conservation_composite_footprint_orphan.RData")
#load("MCF7_conservation_composite_footprint_orphan.RData")
#composites.func.panels.conservation.x(all.composites.conservation, fact = \
    '', summit = 'Motif', num=100)
#composites.func.panels.naked.chromatin(orphan90,fact = 'orphan90_dreme', \
    summit = 'Motif', num=50)
#composites.func.panels.naked.chromatin(orphan90,fact = 'orphan90_dreme', \
    summit = '20bp', num=20)
#composites.func.panels.naked.chromatin(orphan90,fact = 'orphan90_dreme', \
    summit = '100bp', num=100)
```

O Python script: scramble motif from a minimal meme file

```
#! /usr/bin/python
import sys
import getopt
import random
```

```
from random import shuffle
def matrix(filename):
    infile=open(filename, 'r')
    nmotifs = 0
    directory = filename.split('.txt')[0]
    outfilename_prefix = directory + '_scramble.txt'#test_creb1'#directory \
    + splitline[1].split('.')[0]
    print outfilename_prefix
    outfile=open(outfilename_prefix, 'w')
    lst=[]
    while 1:
        line = infile.readline()
        if not line: break
        splitline = line.split()
        if line.startswith('URL'): continue
        if len(splitline) != 4:
            if not line.startswith('MOTIF'):
                outfile.write(line)
            else:
                outfile.write("%s\n"%(splitline[0] + ' ' + splitline[1] + '\
    _scramble'))
        else:
            lst.append(line)
    shuffle(lst)
    for item in lst:
        outfile.write("%s" % item)
    outfile.close()
    return

def main(argv):
    try:
        opts, args = getopt.getopt(argv, "hi:", ["help", "input="])
    except getopt.GetoptError, err:
        print str(err)
        sys.exit(2)
    name = False
    for opt, arg in opts:
        if opt in ('-i', '--input'):
            name = arg
        elif opt in ('-h', '--help'):
            print 'python ~/pyscripts/meme_scramble.py -i /Users/guertinmj/\
    meme_project/each_minimal_meme_file/CREB1_meme.txt'
            sys.exit()
        matrix(name)
if __name__ == "__main__":
    main(sys.argv[1:])
```

P R script: production of composite plots for DNase-seq and conservation data with scrambled orphan MAST output files

```
#libraries
library(lattice)
library(bigWig)
library(latticeExtra)

#functions
```

```
composites.test <- function(path.dir, composite.input, region=20, step=1, \
    grp = 'DNase') {
  vec.names = c('chr','start','end')
  hmap.data = list()
  composite.df=data.frame(matrix(ncol = 6, nrow = 0))
  for (mod.bigWig in Sys.glob(file.path(path.dir, "*.bigWig"))) {
    factor.name = strsplit(strsplit(mod.bigWig, "/")[[1]][length(strsplit(\
   mod.bigWig, "/")[[1]])], '\\.')[[1]][1]
    print(factor.name)
    vec.names = c(vec.names, factor.name)
    wiggle = load.bigWig(mod.bigWig)
    bpquery = window.step(composite.input, wiggle, region, step)
    subsample = subsampled.quantiles.metaprofile((bpquery[[1]]))#\
   alternative functions: bootstrapped.confinterval.metaprofile, \
   confinterval.metaprofile, subsampled.quantiles.metaprofile
    mult.row = ncol(bpquery[[1]])
    hmap.data[[factor.name]] = bpquery[[1]]
    df.up <- data.frame(matrix(ncol = 6, nrow = mult.row))
    df.up[, 1] <- subsample$middle
    df.up[, 2] <- seq((-1 * region) + 0.5 * step, region - 0.5 * step, by =\
     step)
    df.up[, 3] <- matrix(data = factor.name, nrow=mult.row, ncol=1)
    df.up[, 4] <- subsample$top
    df.up[, 5] <- subsample$bottom
    df.up[, 6] <- matrix(data = grp, nrow=mult.row, ncol=1)
    composite.df = rbind(composite.df, df.up)
    unload.bigWig(wiggle)
  }

  colnames(composite.df) <- c('est', 'x', 'cond', 'upper', 'lower', 'grp')
  composite.df = composite.df[composite.df[,2] >= -1000 & composite.df[,2] \
   <= 1000,]
  for (cond in (1:length(hmap.data))) {
    rownames(hmap.data[[cond]]) = paste(composite.input[,1], ':', composite\
   .input[,2], '-', composite.input[,3], sep='')
    colnames(hmap.data[[cond]]) = seq((-1 * region) + 0.5 * step, region - \
   0.5 * step, by = step)
  }
  return(list(composite.df, hmap.data))
}

processes.comps <- function(fimo = 'mast_hotspots_r4_FOSL2.txt',
                            fimobed='mast_hotspots_r4_FOSL2.bed',
                            hotspots = 'Hotspots_Either_E2_condition_merged\
   .bed',
                            path.dir = 'UW_DNase', region = 200)
  {
  factor.name = strsplit(strsplit(fimo, "/mast_")[[1]][length(strsplit(fimo\
   , "/mast_")[[1]])], '\\.txt')[[1]][1]
  print(factor.name)
  all.fimo.bed = parse.mast(fimobed)
  print('rows in original bed')
  print(nrow(all.fimo.bed))
  all.fimo.bed = all.fimo.bed[all.fimo.bed[,2] > region,]#remove rows off \
   teh end of chrromosome

  chrom.bW = load.bigWig(Sys.glob(file.path(path.dir, "*.bigWig"))[1])
  for (i in 1:length(chrom.bW$chroms)) {
    all.fimo.bed = subset(all.fimo.bed, (all.fimo.bed[,1] == chrom.bW$\
```

```
    chroms[i]) & (all.fimo.bed[,3] < chrom.bW$chromSizes[i]) | all.fimo.bed\
    [,1] != chrom.bW$chroms[i])
  }
  print('rows in bed after filtering coordiates that reside off the \
    chromosome boundaries')
  print(nrow(all.fimo.bed))
  hotspots = read.table(hotspots, header=F)
  fimo.overlap.hotspots = bedTools.intersect(bed1 = hotspots, bed2=all.fimo\
    .bed, opt.string ='-wb')
  fimo.composites = composites.test(path.dir, fimo.overlap.hotspots, region\
     = region, grp = factor.name)
  return(fimo.composites)
}


parse.mast <- function(file) {
  mast.data = read.table(file, colClasses=c('character','integer','integer'\
    ,'numeric', 'numeric','character','character'))
  res = mast.data
  colnames(res) = c('chr', 'start', 'end', 'score', 'pval', 'strand', '\
    motif')
  return(res)
}


window.step <- function(bed, bigWig, halfWindow, step) {
  windowSize = (2*halfWindow) %/% step
  midPoint = floor((as.numeric(as.character(bed[,2])) + as.numeric(as.\
    character(bed[,3]))) / 2)
  start = (midPoint - halfWindow)
  end = start + windowSize*step
  if ((as.numeric(as.character(bed[1,2])) + as.numeric(as.character(bed\
    [1,3]))) %% 2 == 0) {
     bed[,2] = start
     bed[,3] = end
  } else {
     bed[,2] = start
     bed[,3] = end
     bed[,2][bed[,6] == '-'] = bed[,2][bed[,6] == '-'] + 1
     bed[,3][bed[,6] == '-'] = bed[,3][bed[,6] == '-'] + 1
  }
  matrix.comp = bed6.step.bpQuery.bigWig(bigWig, bigWig, bed, 1, op = "avg"\
    , follow.strand = TRUE)
  res = do.call(rbind, matrix.comp)
  return(list(res, matrix.comp))
}

bedTools.intersect<-function(functionstring="/usr/local/bin/bedtools2/\
    intersectBed",bed1,bed2,opt.string="") {
  #create temp files
  a.file=tempfile()
  b.file=tempfile()
  out   =tempfile()
  options(scipen =99) # not to use scientific notation when writing out

  #write bed formatted dataframes to tempfile
  write.table(bed1,file=a.file,quote=F,sep="\t",col.names=F,row.names=F)
  write.table(bed2,file=b.file,quote=F,sep="\t",col.names=F,row.names=F)
```

```
  # create the command string and call the command using system()
  command=paste(functionstring, opt.string,"-a",a.file,"-b",b.file,">",out,\
    sep=" ")
  cat(command,"\n")
  try(system(command))

  res=read.table(out,header=F, comment.char='')
  unlink(a.file);unlink(b.file);unlink(out)
  return(res[,c(4,5,6,7,8,9)])
}


my.panel.bands <-
    function(x, y, upper, lower,
             fill, col,
             subscripts, ..., font, fontface)
{
    upper <- upper[subscripts]
    lower <- lower[subscripts]
    panel.polygon(c(x, rev(x)), c(upper, rev(lower)),
                  col = fill, border = FALSE,
                  ...)
}

composites.func.panels.naked.chromatin <- function(dat, fact = 'Factor', \
    summit = 'Summit', num=90) {
  col.lines = c(rgb(0,0,1,1/2), rgb(1,0,0,1/2), "purple", "#0faa12")
  count = length(unique(dat$grp))
  pdf(paste('composite_', fact, '_signals_', summit, '_peaks.pdf', sep=''),\
     width=2* 3.43, height=ceiling((count/2)) * 3.43)
  print(xyplot(est ~ x|grp, group = cond, data = dat,
               type = 'l',
               scales=list(x=list(cex=0.8,relation = "free"), y =list(cex\
    =0.8, relation="free")),
               xlim=c(-(num),(num)),
               col = col.lines,
               auto.key = list(points=F, lines=T, cex=0.8),
               par.settings = list(superpose.symbol = list(pch = c(16), col\
    =col.lines), superpose.line = list(col = col.lines, lwd=3)),
               cex.axis=1.0,
               par.strip.text=list(cex=0.9, font=1, col='black'),
               aspect=1.0,
               between=list(y=0.5, x=0.5),
               lwd=3,
               ylab = list(label = paste(fact," Cut Frequency", sep=''), \
    cex =0.8),
               xlab = list(label = paste("Distance from ", summit, " center\
    ",sep=''), cex =0.8),
               upper = dat$upper,
               fill = c(rgb(0,0,1,1/4), rgb(1,0,0,1/4), "purple", "#0faa12"\
    ),
               lower = dat$lower,
               strip = function(..., which.panel, bg) {
                 bg.col = c("grey85")# "grey40","#0a823c", "#0a823c") #blue\
    , red green
                 strip.default(..., which.panel = which.panel, bg = rep(bg.\
    col, length = which.panel)[which.panel])
             },
               panel = function(x, y, ...){
```

```
                    panel.superpose(x, y, panel.groups = 'my.panel.bands', \
    ...)
                    panel.xyplot(x, y, ...)
         }
  ))
  dev.off()
}

composites.func.panels.conservation.x <- function(dat, fact = '', summit = \
    'Summit', num=20) {
  col.lines = c(rgb(0,0,1,2/3), rgb(1,0,0,2/3))
  count = length(unique(dat$grp))
  cons.dat = dat[dat$cond == 'phastCon',]
  phylo.dat = dat[dat$cond == 'phyloP',]
  pdf(paste('composite_', fact, '_conservation_', summit, '_peaks.pdf', sep\
    =''), width=2*3.43, height=(count/2) * 3.43)
  cons = xyplot(est ~ x|grp, group = cond, data = cons.dat,
      type = 'l',
      scales=list(x=list(cex=0.8, relation = "free"), y =list(cex=0.8, \
    relation="free")),
      xlim=c(-(num),(num)),
      col = col.lines,
      key = list(lines=list(col=col.lines, lwd=3), text = list(c('phastCons\
    ','phyloP')), cex=0.8),
      par.settings = list(superpose.symbol = list(pch = c(16), col='black')\
    , superpose.line = list(col = rgb(0,0,1,1/2), lwd=1)),
      cex.axis=1.0,
      par.strip.text=list(cex=0.9, font=1, col='black'),
      aspect=1.0,
      fill = c(rgb(0,0,1,1/4), rgb(1,0,0,1/4), "purple", "#0faa12"),
      between=list(y=0.5, x=5.5),
      lwd=3,
      ylab = list(label = paste(fact,"Average Conservation Score", sep=''),\
     cex =0.8),
      xlab = list(label = paste("Distance from ", summit, " center",sep='')\
    , cex =0.8),
      upper = cons.dat$upper,
      lower = cons.dat$lower,
      strip = function(..., which.panel, bg) {
          bg.col = c("grey85")# "grey40","#0a823c", "#0a823c") #blue, red \
    green
          strip.default(..., which.panel = which.panel, bg = rep(bg.col, \
    length = which.panel)[which.panel])
      },
      panel = function(x, y, ...){
          panel.superpose(x, y, panel.groups = 'my.panel.bands', ...)
          panel.xyplot(x, y, ...)
      }
                  )
  phylo = xyplot(est ~ x|grp, group = cond, data = phylo.dat,
      type = 'l',
      scales=list(x=list(cex=0.8, relation = "free"), y =list(cex=0.8, \
    relation="free")),
      xlim=c(-(num),(num)),
      col = rev(col.lines),
      par.settings = list(superpose.symbol = list(pch = c(16), col='black')\
    , superpose.line = list(col = rgb(1,0,0,1/2), lwd=1)),
      cex.axis=1.0,
      par.strip.text=list(cex=0.9, font=1, col='black'),
```

```
      aspect=1.0,
      fill = rev(c(rgb(0,0,1,1/4), rgb(1,0,0,1/4))),
      between=list(y=0.5, x=5.5),
      lwd=3,
      ylab = list(label = paste(fact,"Average Conservation Score", sep=''),\
     cex =0.8),
      xlab = list(label = paste("Distance from ", summit, " center",sep='')\
    , cex =0.8),
      upper = phylo.dat$upper,
      lower = phylo.dat$lower,
      strip = function(..., which.panel, bg) {
          bg.col = c("grey85")# "grey40","#0a823c", "#0a823c") #blue, red \
    green
          strip.default(..., which.panel = which.panel, bg = rep(bg.col, \
    length = which.panel)[which.panel])
      },
      panel = function(x, y, ...){
        panel.superpose(x, y, panel.groups = 'my.panel.bands', ...)
        panel.xyplot(x, y, ...)
      }
                  )
  print(doubleYScale(phylo, cons))
  dev.off()
}

change.format <- function(process.pipe.output, min = -6, max = 6) {
  process.pipe.output = process.pipe.output[[1]]
  #process.pipe.output$cond[process.pipe.output$cond == 'UW_MCF7_both'] = '\
    Chromatin'
  #process.pipe.output$cond[process.pipe.output$cond == 'IMR90_Naked_DNase\
    '] = 'Naked DNA'
  process.pipe.output$cond[process.pipe.output$cond == 'MCF7_Chromatin_6-\
    mer'] = 'Chromatin'
  process.pipe.output$cond[process.pipe.output$cond == 'Naked_DNA_6-mer'] =\
     'Naked DNA'
  all.ctcf.norm = norm.com.df(process.pipe.output[process.pipe.output$cond \
    == 'Naked DNA',], process.pipe.output[process.pipe.output$cond == '\
    Chromatin' & process.pipe.output$x >= min & process.pipe.output$x <= max\
    ,])
  w = rbind(process.pipe.output[process.pipe.output$cond == 'Chromatin',], \
    all.ctcf.norm)
  return(w)
}

norm.com.df <- function(comp.df, norm.to) {
  min.norm = min(norm.to$est)
  min.comp = min(comp.df$est[comp.df$est!=0])
  fact = min.norm/min.comp
  comp.df$est = comp.df$est*fact
  zeroed.df = comp.df$est - min(comp.df$est[comp.df$est!=0])
  zeroed.df = zeroed.df / max(zeroed.df)
  max.norm.minus.min.comp = max(norm.to$est) - min(comp.df$est[comp.df$est!\
    =0])
  new = min(comp.df$est[comp.df$est!=0]) + zeroed.df * max.norm.minus.min.\
    comp
  comp.df$est = new
  min.norm = min(norm.to$upper)
  min.comp = min(comp.df$upper[comp.df$upper!=0])
  fact = min.norm/min.comp
```

```
  comp.df$upper = comp.df$upper*fact
  zeroed.df = comp.df$upper - min(comp.df$upper[comp.df$upper!=0])
  zeroed.df = zeroed.df / max(zeroed.df)
  max.norm.minus.min.comp = max(norm.to$upper) - min(comp.df$upper[comp.df$\
    upper!=0])
  new = min(comp.df$upper[comp.df$upper!=0]) + zeroed.df * max.norm.minus.\
    min.comp
  comp.df$upper = new
  min.norm = min(norm.to$lower)
  min.comp = min(comp.df$lower[comp.df$lower!=0])
  fact = min.norm/min.comp
  comp.df$lower = comp.df$lower*fact
  zeroed.df = comp.df$lower - min(comp.df$lower[comp.df$lower!=0])
  zeroed.df = zeroed.df / max(zeroed.df)
  max.norm.minus.min.comp = max(norm.to$lower) - min(comp.df$lower[comp.df$\
    lower!=0])
  new = min(comp.df$lower[comp.df$lower!=0]) + zeroed.df * max.norm.minus.\
    min.comp
  comp.df$lower = new
  return(comp.df)
}

cycle.fimo <- function(path.dir.fimo = '~/mast_file_for_conservation', path\
    .dir.bigWig = '~/UW_DNase_MCF7', hotspot.file = 'Hotspots_Either_E2_\
    condition.merge.broadPeak', window = 200) {
  composite.df=data.frame(matrix(ncol = 6, nrow = 0))
  for (fimo.file in Sys.glob(file.path(path.dir.fimo, "*mast_output/*.txt")\
    )) {
     factor.name = strsplit(strsplit(fimo.file, "mast_")[[1]][length(\
    strsplit(fimo.file, "mast_")[[1]])], '\\.')[[1]][1]
     print(factor.name)
     tf.x = processes.comps(fimo = fimo.file, fimobed=paste0(strsplit(fimo.\
    file,'.txt'),'.bed'),
                                hotspots = hotspot.file, path.dir =  path.dir.\
    bigWig, region = window)
     plot.composite.fimo = change.format(tf.x, -10, 10)#10 is hardcoded, it \
    may vary per fimo output
     composite.df = rbind(composite.df, plot.composite.fimo)
  }
  composites.func.panels.naked.chromatin(composite.df, 'DNase', 'Motif', \
    num =100)#this is hard coded and shouldn't be
  return(composite.df)
}

cycle.fimo.conservation <- function(path.dir.fimo = '~/mast_file_for_\
    conservation', path.dir.bigWig = '~/UW_DNase_MCF7', hotspot.file = '\
    iterative/Hotspots_Either_E2_condition.merge.broadPeak', window = 200) {
  composite.df=data.frame(matrix(ncol = 6, nrow = 0))
  for (fimo.file in Sys.glob(file.path(path.dir.fimo, "*mast_output/*.txt")\
    )) {
     factor.name = strsplit(strsplit(fimo.file, "mast_")[[1]][length(\
    strsplit(fimo.file, "mast_")[[1]])], '\\.')[[1]][1]
     print(factor.name)
     tf.x = processes.comps(fimo = fimo.file, fimobed=paste0(strsplit(fimo.\
    file,'.txt'),'.bed'),
                                hotspots = hotspot.file, path.dir =  path.dir.\
    bigWig, region = window)
     print(head(tf.x[[1]]))
     composite.df = rbind(composite.df, tf.x[[1]])
```

```
  }
  composites.func.panels.conservation.x(composite.df, summit = 'Motif', num\
     =100)
  return(composite.df)
}

#processes
#for all the non-orphan motifs
orphan=c('~/Desktop/Iterate_MCF7_hotspot_onestop/iteration/scramble_mast_\
    files_for_conservation_plots_orphan')
all.composites.dnase =
  cycle.fimo(path.dir.fimo = orphan,
             path.dir.bigWig = '~/Desktop/normalized_bigWig/normalized_\
    bigwig/MCF7',
             hotspot.file = '~/iterative/Hotspots_Either_E2_condition.merge\
    .broadPeak', window = 200)

all.composites.conservation =
  cycle.fimo.conservation(path.dir.fimo = orphan,
                          path.dir.bigWig = '~/conservation_files',
                          hotspot.file = '~/iterative/Hotspots_Either_E2_\
    condition.merge.broadPeak', window = 200)

save(all.composites.dnase,all.composites.conservation,file="MCF7_\
    conservation_composite_footprint_orphan_scramble.RData")
#load("MCF7_conservation_composite_footprint_orphan.RData")
#composites.func.panels.conservation.x(all.composites.conservation, fact = \
    '', summit = 'Motif', num=100)
```

Q Python script: production of degenerate PSWM from a minimal meme file

```
#! /usr/bin/python
import sys
import getopt
import numpy as np
def matrix(filename, outfilename_prefix):
    infile=open(filename, 'r')
    nmotifs = 0
    outfile=open(str(outfilename_prefix) + '_degenerate.txt', 'w')
    while 1:
        line = infile.readline()
        if not line: break
        splitline = line.split()
        directory = filename.split('meme.txt')[0]
        if line.startswith('MEME version'):
            meme_line = line
            outfile.write(line)
            outfile.write('\n')
        if line.startswith('ALPHABET'):
            alphabet_line = line
            outfile.write(line)
            outfile.write('\n')
        if line.startswith('strands:'):
            strands_line = line
            outfile.write(line)
            outfile.write('\n')
```

```
        #if line.startswith('Background letter frequencies'):
        #    outfile.write(line)
        if line.startswith('A 0.'):
            background = line
            outfile.write(background)
            outfile.write('\n')
        if line.startswith('letter-probability matrix:'):
            nmotifs += 1
            motif_len = splitline[5]
            lst = []
            outfile.write('%s%s%s\n'%('MOTIF ', outfilename_prefix, '_' + '\
full'))
            outfile.write(line)
            lpm = line
            motif_len = splitline[5]
            while 1:
                next = infile.readline()
                lst.append(next)
                if len(next.split()) != 4: break
                outfile.write(next)
            outfile.write('\n')
            for position in range(1,len(lst)):
                first = lst[:position - 1]
                last = lst[position:]
                #uncomment the following will produce a file cancatenating \
different minimal meme files
                outfile.write(meme_line)
                outfile.write('\n')
                outfile.write(alphabet_line)
                outfile.write('\n')
                outfile.write(strands_line)
                outfile.write('\n')
                outfile.write(background)
                outfile.write('\n')
                outfile.write('%s%s%s\n'%('MOTIF ', outfilename_prefix, '_'\
 + 'pos_' + str(position)))
                outfile.write(lpm)
                for item in first:
                    outfile.write("%s" % item)
                outfile.write("%s\t%s\t%s\t%s\n"%(' ' + background.split()\
[1], background.split()[3], background.split()[5], background.split()\
[7]))
                for item in last:
                    outfile.write("%s" % item)
            outfile.write('\n')
    outfile.close()
    return

def main(argv):
    try:
        opts, args = getopt.getopt(argv, "hi:o:", ["help", "input=", "out="\
])
    except getopt.GetoptError, err:
        print str(err)
        sys.exit(2)
    name = False
    outname = False
    for opt, arg in opts:
        if opt in ('-i', '--input'):
```

```
            name = arg
        if opt in ('-o', '--out'):
            outname = arg
        elif opt in ('-h', '--help'):
            print 'python MEME_full_degeneracy.py -i minimal_meme_r1_15.txt\
     -o outfileprefix'
            sys.exit()
    if name and outname:
        matrix(name, outname)
if __name__ == "__main__":
    main(sys.argv[1:])
```

R R script: obtain the information content in single nucleotide resolution with a designated threshold by André Martins

```
#! /usr/bin/env Rscript

#' Load PWM & background freqs from minimal MEME file
#'
#' Obtains the alphabet, background base frequencies and motif frequencies \
    (PWM) from a MEME file.
#' Assumes that file contains only a single PWM.
#'
#' @param filename Path to the MEME file.
#' @return list with 'pwm', the number of sites used to build the PWM ('n.\
    sites'), the alphabet and the background frequencies
options(scipen=999)
meme.parse <- function(filename) {
        lines = readLines(filename)
        empty_lines_idx = which(grepl("^$", lines))
        motif_start_idx = which(grepl("letter-probability matrix", lines)) \
    + 1
        motif_end_idx = empty_lines_idx[which(empty_lines_idx > motif_start\
    _idx)[1]] - 1

        # assuming there is only one motif in the file
        pwm = read.table(filename, skip = motif_start_idx - 1, nrows = \
    motif_end_idx - motif_start_idx + 1)

        # get number of sequences used to compute pwm
        txt = lines[motif_start_idx - 1]
        txt = regmatches(txt,regexpr("nsites=[ ]+[0-9]+",txt))
        n.sites = as.integer(unlist(strsplit(txt, "="))[2])

        # get alphabet
        alphabet = trimws(strsplit(lines[grepl("ALPHABET", lines)], "=")\
    [[1]][2])

        # get background frequencies
        background_line = lines[which(grepl("Background letter frequencies"\
    , lines))+1]
        bfreqs = as.numeric(strsplit(background_line, " ")[[1]][c(2,4,6,8)\
    ])

        # return data
```

```
        list(pwm = pwm, n.sites = n.sites, alphabet = alphabet, background \
    = bfreqs)
}

#' Compute information content for single position frequencies
#'
#' @param freqs vector of symbol frequencies
#' @param bfreqs vector of background frequencies
#' @return information content value
information.content <- function(freqs, bfreqs = NULL) {
        if (is.null(bfreqs)) {
                2 + sum(sapply(freqs, function(f) {
                        if (f == 0)
                                0
                        else
                                f * log2(f)
                }))
        } else {
                sum(sapply(1:length(freqs), function(idx) {
                        if (freqs[idx] == 0)
                                0
                        else
                                freqs[idx] * log2(freqs[idx] / bfreqs[idx])
                }))
        }
}

#' Compute string of prefered PWM bases
#'
#' @param meme return value from meme.parse() function
#' @return list with plus strand bases and minus strand bases
prefered.bases <- function(meme) {
        # get plus strand version
        idxs = apply(meme$pwm, 1, which.max)
        bases = unlist(strsplit(meme$alphabet,""))
        pref = bases[idxs]

        # get reverse-complement version
        rpref = sapply(rev(pref), function(base) {
                if (base == "A") return("T")
                if (base == "C") return("G")
                if (base == "G") return("C")
                if (base == "T") return("A")
                "N"
        })
        names(rpref) <- NULL

        list(plus = pref, minus = rpref)
}

#' Pre-computes per-site output data
#'
#' @param meme return value from meme.parse() function
#' @param use.ic If TRUE use standard information content (ignore \
    background frequencies)
#' @param threshold output only bases with IC >= threshold
#' @param use.correction apply small-sample correction
#' @return list with information content, plus strand bases, minus strand \
    bases and base offsets after filter
```

```
prepare.data <- function(meme, use.ic = FALSE, threshold = -Inf, use.\
    correction = FALSE) {
        # compute information content
        ic = NULL
        if (use.ic) {
                ic = apply(meme$pwm, 1, information.content)
        } else {
                ic = apply(meme$pwm, 1, function(row) {
                        information.content(row, meme$background)
                })
        }

        if (use.correction) {
                # small sample correction
                # J Mol Biol. 1986 Apr 5;188(3):415-31.
                s = 4 # number of bases
                en = 1/log(2) * (s - 1) / (2 * meme$n.sites)
                ic = ic - en
        }

        # PWM prefered bases
        pwm.bases = prefered.bases(meme)

        # filter
        keep = ic >= threshold

        list(ic = ic[keep], plus = pwm.bases$plus[keep], minus = pwm.bases$\
    minus[rev(keep)], offsets.plus = which(keep) - 1, offsets.minus = which(\
    rev(keep)) - 1)
}

#' Load a txt MAST file as a BED data.frame
#'
#' @param filename path to the MAST file
#' @return data.frame with MAST data in BED format
load.txtmast <- function(filename) {
        tbl = read.table(filename, stringsAsFactors = FALSE, colClasses =c(\
    "character", "character", "numeric", "numeric", "numeric", "numeric"))
        strands = substr(tbl[,2], 1, 1)
        ids = substr(tbl[,2], 2, nchar(tbl[,2]))

        parts = strsplit(tbl[,1], "[:-]")
        chroms = sapply(parts, function(row) row[1])
        start = sapply(parts, function(row) as.numeric(row[2]))

        data.frame(
                chroms,
                start + tbl[,3],
                start + tbl[,4],
                tbl[,5:6],
                strands,
                ids
        )
}

#' Write output file with base-pair information for the PWM sites reported \
    in the MAST file.
#'
#' Assumes that MAST was run with a single motif.
```

```
#'
#' @param output.filename Path for output file
#' @param mast.bed data.frame with contents of BED file containing MAST \
    sites (NOTE: requires that data.frame was built with stringsAsFactors = \
    FALSE)
#' @param pwm.data output of prepare.data function
process.mast <- function(output.filename, mast.bed, pwm.data) {
        out = file(output.filename, "w")
        ic.str = format(pwm.data$ic)
        ic.rstr = rev(ic.str)
        is.minus = mast.bed[,6] == '-'

        for (i in 1:dim(mast.bed)[1]) {
                bases = pwm.data$plus
                ics = ic.str
        positions = pwm.data$offsets.plus + mast.bed[i, 2]
                if (is.minus[i]) {
                        bases = pwm.data$minus
                        ics = ic.rstr
            positions = pwm.data$offsets.minus + mast.bed[i, 2]
                }
                bed.i = data.frame(
                        chrom = mast.bed[i, 1],
                        start = positions,
                        end = positions + 1,
                        bases = bases,
                        ic = ics,
                        strand = mast.bed[i, 6],
                        stringsAsFactors = FALSE
                )
                write.table(bed.i, file = out, quote = FALSE, sep = "\t", \
    row.names = FALSE, col.names = FALSE)
        }
        close(out)
}

########################################################################

# running in Rscript
if (!interactive()) {
        # build usage message
        all.args = commandArgs(trailingOnly = FALSE)
        scriptName <- basename(sub("^--file=", "", all.args[grep("^--file="\
    , all.args)]))

        short.usage = paste("Usage:", scriptName, " [options] <MEME file> <\
    MAST file> [<output file>]\n      ", scriptName, " -h\n")

        # collect command arguments
        cmd.args = commandArgs(trailingOnly = TRUE)
        is.option = grepl("^-", cmd.args, perl = TRUE)
        options = cmd.args[is.option]
        main.args = cmd.args[!is.option]

        # help requested
        if (any(options == "-h")) {
                cat("Usage:", scriptName, "[options] <MEME file> <MAST file\
    > [<output file>]\n")
                cat("\n")
```

```
                cat("Options:\n")
                cat("--thresh=value  minimal value filter (output values >=\
 thresh)\n")
                cat("-i              output information content (default \
relative entropy)\n")
                cat("-c              apply small sample correction to \
information content\n")
                cat("-o              override output file if it exists\n")
                cat("-h              show this help message\n")
                cat("\n")
                quit(save = "no", status = 1, runLast = FALSE)
        }

        # other options
        use.ic = any(options == "-i")
        use.correction = any(options == "-c")
        override.output = any(options == "-o")
        thresh = -Inf
        aux = grepl("^--thresh=", options)
        if (sum(aux) == 1) {
                idx = which(aux)
                parts = unlist(strsplit(options[idx], "="))
                thresh = as.numeric(parts[2])
        } else if (sum(aux) > 1) {
                cat("ERROR: Repeated threshold option\n")
                cat(short.usage)
                quit(save = "no", status = 1, runLast = FALSE)
        }

        # test for invalid options
        invalid = (
                options != "-i" &
                options != "-c" &
                options != "-o" &
                !grepl("^--thresh=", options)
                )
        if (any(invalid)) {
                cat("ERROR: Invalid option(s):", options[invalid], "\n")
                cat(short.usage)
                quit(save = "no", status = 1, runLast = FALSE)
        }

        # main arguments
        n.args = sum(!is.option)
        if (n.args != 2 && n.args != 3) {
                cat("ERROR: Not enough arguments\n")
                cat(short.usage)
                quit(save = "no", status = 1, runLast = FALSE)
        }
        meme.filename = main.args[1]
        mast.filename = main.args[2]
        mast.is.bed = regexpr("bed$", mast.filename) >= 0
        output.filename = ""
        if (n.args == 3) {
                output.filename = main.args[3]
        } else {
                idxs = regexpr("[.]", mast.filename)
                if (length(idxs) == 0) {
                        output.filename = paste(mast.filename, "output.bed"\
```

```
, sep="_")
                } else {
                        idx = idxs[length(idxs)]
                        output.filename = paste(substr(mast.filename, 1, \
idx - 1) ,"output.bed", sep="_")
                }
        }

        # check files exist
        if (!file.exists(meme.filename)) {
                cat("ERROR: Can't find MEME file:", meme.filename, "\n")
                quit(save = "no", status = 1, runLast = FALSE)
        }
        if (!file.exists(mast.filename)) {
                cat("ERROR: Can't find MAST file:", mast.filename, "\n")
                quit(save = "no", status = 1, runLast = FALSE)
        }
        if (!override.output && file.exists(output.filename)) {
                cat("ERROR: Can't find output file already exists:", output\
.filename, "\n")
                quit(save = "no", status = 1, runLast = FALSE)
        }

        ##

        # parse MEME data
        meme.data = meme.parse(meme.filename)

        # pre-compute data
        pwm.data = prepare.data(meme.data, use.ic = use.ic, threshold = \
thresh, use.correction = use.correction)

        # load MAST file
        # NOTE: processing function requires stringsAsFactors = FALSE
        mast.bed = NULL
        if (mast.is.bed) {
                mast.bed = read.table(mast.filename, stringsAsFactors = \
FALSE)
        } else {
                mast.bed = load.txtmast(mast.filename)
        }

        # process data
        time.before = proc.time()
        process.mast(output.filename, mast.bed, pwm.data)
        time.after = proc.time()

        #
        cat(dim(mast.bed)[1], "sites processed in", time.after[3] - time.\
before[3], "seconds.\n")
}
```

S Bash shell script: obtain the TCGA patient genotype data from birdseed files and imputation with Michigan Imputation Server

```
#navigate into the directory containing all the birdseed files
```

```
cd <dir>
#unzip all the zipped birseed file
gunzip *

#make sure that "broad.mit.edu_BRCA.Genome_Wide_SNP_6.sdrf.birdseed.normal.\
    names.txt" is in the same foler as all the unzipped "birseed file"
cp ~/broad.mit.edu_BRCA.Genome_Wide_SNP_6.sdrf.birdseed.normal.names.txt <\
    dir>

#give all files TCGA names and make the header suitable for birdseed to VCF\
     script
cat broad.mit.edu_BRCA.Genome_Wide_SNP_6.sdrf.birdseed.normal.names.txt | \
    while read line;
do
        tcga=$(echo $line | awk -F" " '{print $1}')
        file=$(echo $line | awk -F" " '{print $2}')
        echo file
        echo $file
        echo tcga
        echo $tcga
        touch temp.txt
        echo -e "probeset_id\t$tcga" >> temp.txt
        tail +3 $file > tail.temp.txt
        cat temp.txt tail.temp.txt > ${tcga}_birdseed.txt
        rm temp.txt
        gzip $file
done

#check out how many bird seed files
ls *birdseed.txt | wc -l
#975

#delete files that don't have genotypes
find . -name "*birdseed.txt" -size -100 -delete

#check out how many bird seed files left
ls *birdseed.txt | wc -l
#937

#convert to VCF files: https://www.broadinstitute.org/cancer/cga/sites/\
    default/files/data/tools/contest/BirdseedToVCF.py; GenomeWideSNP_6.na30.\
    annot.hg19.csv.pickle downloaded from TCGA
for i in *birdseed.txt
do
        tcga=$(echo $i | awk -F"/" '{print $NF}' | awk -F"_birdseed" '{\
    print $1}')
        echo $tcga
        python ~/BirdseedToVCF.py --birdseed $i --output_vcf ${tcga}.vcf --\
    snp_annotation ~/GenomeWideSNP_6.na30.annot.hg19.csv.pickle --fasta ~/\
    hg19.fa --array_sample ${tcga} --vcf_sample ${tcga} --add_chr
        gzip $i
done
#note that this step is time consuming, took about 11 hours on my mac

#separte vcf by chromosomes
#first install gawk
brew install gawk

for i in TCGA*.vcf
```

```
do
        name=$(echo $i | awk -F"/" '{print $NF}' | awk -F".vcf" '{print $1\
    }')
        echo $name
        gawk 'BEGIN{Ch=""}{if($1~"#"){H[FNR]=$0;nh++}else{if($1!=Ch){Ch=$1;\
    Out=$1"_'"$name"'.vcf";for(i=1;i<=nh;i++){print H[i] >Out}}; print $0 >\
    Out}}' $i
done

#note: need to install vcftools here?
#remove chromsome and make into vcf.gz file
for i in chr*.vcf
do
        chrX=$(echo $i | awk -F"/" '{print $NF}' | awk -F"_TCGA" '{print $1\
    }')
        name=$(echo $i | awk -F"/" '{print $NF}' | awk -F".vcf" '{print $1\
    }')
        echo $name
        awk '{gsub(/^chr/,""); print}' $i > ${name}.temp.vcf
        vcf-sort ${name}.temp.vcf | bgzip -c > ${name}.vcf.gz
        rm ${name}.temp.vcf
done

#first download and install "bcftools"
#http://www.htslib.org/download/
#bcftools-1.3.1: file "bcftools-1.3.1.tar.bz2", and unzip this foler
#refer to "~/Downloads/bcftools-1.3.1/INSTALL" for installation \
    instructions
cd ~/Downloads/bcftools-1.3.1
make
make install

#navigate into the directory containing all the originial birdseed files
cd <dir>
for i in chr*.vcf.gz
do
name=$(echo $i | awk -F"/" '{print $NF}' | awk -F".vcf" '{print $1}')
bcftools index $i
done

#merge vcf files for imputation
#note this is path where vcftools paths were downloaded
export PERL5LIB=~/Downloads/vcftools/src/perl

#first test whether it is working for chr1, note that this step is memory \
    consuming, so that we break all the files into three parts, and merge \
    them seperately first, and then merge those three merged files again.
#if you see "Too many open files" errors, run
ulimit -n
#if it is below 500, run the following command to change it to 1000 for the\
     current shell and its children
ulimit -n 1000

#this is just for chr19
#for chr in {19}; do
#    echo chr$chr
#    vcf-merge $(ls -1 chr${chr}_TCGA-A*.vcf.gz | perl -pe 's/\n/ /g') | \
    bgzip -c > TCGA_BrCA_chr${chr}_TCGA-A_merged.vcf.gz
#    vcf-merge $(ls -1 chr${chr}_TCGA-B*.vcf.gz | perl -pe 's/\n/ /g') | \
```

```
    bgzip -c > TCGA_BrCA_chr${chr}_TCGA-B_merged.vcf.gz
#     vcf-merge $(ls -1 chr${chr}_*.vcf.gz | grep -v chr${chr}_TCGA-A | grep\
     -v chr${chr}_TCGA-B | perl -pe 's/\n/ /g') | bgzip -c > TCGA_BrCA_chr${\
    chr}_TCGA-other_merged.vcf.gz
#     bcftools index TCGA_BrCA_chr${chr}_TCGA-A_merged.vcf.gz
#     bcftools index TCGA_BrCA_chr${chr}_TCGA-B_merged.vcf.gz
#     bcftools index TCGA_BrCA_chr${chr}_TCGA-other_merged.vcf.gz
#     vcf-merge $(ls -1 TCGA_BrCA_chr${chr}_TCGA*merged.vcf.gz | perl -pe 's\
    /\n/ /g') | bgzip -c > TCGA_BrCA_chr${chr}_merged.vcf.gz
#done

for chr in {1..22}; do
     echo chr$chr
     vcf-merge $(ls -1 chr${chr}_TCGA-A*.vcf.gz | perl -pe 's/\n/ /g') | \
    bgzip -c > TCGA_BrCA_chr${chr}_TCGA-A_merged.vcf.gz
     vcf-merge $(ls -1 chr${chr}_TCGA-B*.vcf.gz | perl -pe 's/\n/ /g') | \
    bgzip -c > TCGA_BrCA_chr${chr}_TCGA-B_merged.vcf.gz
     vcf-merge $(ls -1 chr${chr}_*.vcf.gz | grep -v chr${chr}_TCGA-A | grep \
    -v chr${chr}_TCGA-B | perl -pe 's/\n/ /g') | bgzip -c > TCGA_BrCA_chr${\
    chr}_TCGA-other_merged.vcf.gz
     bcftools index TCGA_BrCA_chr${chr}_TCGA-A_merged.vcf.gz
     bcftools index TCGA_BrCA_chr${chr}_TCGA-B_merged.vcf.gz
     bcftools index TCGA_BrCA_chr${chr}_TCGA-other_merged.vcf.gz
     vcf-merge $(ls -1 TCGA_BrCA_chr${chr}_TCGA*merged.vcf.gz | perl -pe 's\
    /\n/ /g') | bgzip -c > TCGA_BrCA_chr${chr}_merged.vcf.gz
done
```

T R script: eQTL analysis of our candidate SNPs with genes within 1 Mb

```
rm(list=ls())
sessionInfo()
setwd(<dir>)

########start_here#########
####create_geneloc_1mb.txt_file######
#for the coordinates, I will use the TCGA2START package
#A matrix of information on genes from the human reference genome version \
    19; used when merging
#CNA/CNV data at gene level with CNTools.
library(TCGA2STAT)
data("geneinfo")
#ignore any coordinates on the Y chromosome
newgeneinfo=subset(geneinfo,geneinfo$chrom!='Y')
#right now there is no duplicates
genesloc=newgeneinfo[,c(5,1,2,3)]
genesloc[,2]=paste0('chr',genesloc[,2])
colnames(genesloc)=c('geneid','chr','s1','s2')
write.table(genesloc,file="geneloc_1mb.txt",col.names=T,quote=F,row.names=F\
    )
#end of this section
rm(list=ls())
sessionInfo()

#####create_phenotype_file#######
x = read.csv('TCGA_BRCA_exp_HiSeqV2_PANCAN-2015-02-24/genomicMatrix', sep='\
    \t')
```

```
row.names(x) = x[,1]
x = x[,2:ncol(x)]
t.x=data.frame(t(x))
t.x1=t.x[grep('.01$',row.names(t.x)),]
row.names(t.x1)=sapply(strsplit(row.names(t.x1),'.01$'),function(x) x[[1]])

#add in the selected genes
geneloc=read.table('geneloc_1mb.txt',header = TRUE, stringsAsFactors = \
    FALSE)
genes=unlist(geneloc$geneid)

all.genes.data=t.x1[,colnames(t.x1)%in%genes]#genes shrink from 20530 to \
    18217
all.genes.data=data.frame(t(all.genes.data))
phenopatient=colnames(all.genes.data)

all.genes.data$geneid=rownames(all.genes.data)
phenotype=merge(geneloc,all.genes.data,by='geneid')

newphenotype=phenotype[,c(2,3,4,1)]
newphenotype=data.frame(cbind(newphenotype,phenotype[,-c(1:4)]))
colnames(newphenotype)[1:4]=c('#Chr','start','end','ID')
newphenotype[,1]=as.numeric(as.character(sapply(strsplit(newphenotype[,1],'\
    chr'),function(x) x[2])))
newphenotype=newphenotype[order(newphenotype[,1],newphenotype$start),]
write.table(newphenotype,file='phenotype.bed',quote=F,row.names=F,sep='\t')
#note: if the following command line does not work, try to run the code \
    inside the quote directly in terminal
system("bgzip phenotype.bed && tabix -p bed phenotype.bed.gz")
#run it in terminal directly
#bgzip phenotype.bed && tabix -p bed phenotype.bed.gz
#end of this session, keep the patients corresponding to phenotype file
rm(list=setdiff(ls(), "phenopatient"))

#######create_covariant_file######
source("https://bioconductor.org/biocLite.R")
biocLite("RTCGA")
library(RTCGA)
#from there, you will get the dataset containing the clinical information \
    for TCGA breast cancer
checkTCGA('DataSets', 'BRCA')
#BRCA.Merge_Clinical.Level_1.20160

source('http://bioconductor.org/biocLite.R')
biocLite('RTCGA.clinical')
library(RTCGA.clinical)
infoTCGA()
system("mkdir TCGA")
downloadTCGA(cancerTypes = 'BRCA', dataSet='BRCA.Merge_Clinical.Level_1.201\
    ',destDir='TCGA',
             date = tail( checkTCGA('Dates'), 1)[1] )
library(utils)
rm(list=ls(pattern=glob2rx("merge*")))

#####read in the clinical data####
clinical=read.csv('TCGA/gdac.broadinstitute.org_BRCA.Merge_Clinical.Level_\
    1.2015110100.0.0/BRCA.clin.merged.txt',
                  sep='\t',header=F)
rownames(clinical)=clinical[,1]
```

```
clinical=data.frame(t(clinical[,-1]))
clinicalvar=colnames(clinical)

#aliquoute_barcode contains all the fields specified by the TCGA barcode
#####read in PANCAN data#########
b = read.csv('<dir>/TCGA_BRCA_exp_HiSeqV2_PANCAN-2015-02-24/clinical_data',\
     sep = '\t')
#PANCAN data
#ID
#sampleID
#aliquot_uuid
#X_GENOMIC_ID_TCGA_BRCA_exp_HiSeqV2_exon
#X_GENOMIC_ID_TCGA_BRCA_exp_HiSeqV2_PANCAN
#X_GENOMIC_ID_TCGA_BRCA_G4502A_07_3 X_GENOMIC_ID_TCGA_BRCA_exp_HiSeqV2_\
    percentile
#X_GENOMIC_ID_TCGA_BRCA_exp_HiSeqV2
b1=b[,colnames(b)%in%c('sampleID','X_GENOMIC_ID_TCGA_BRCA_exp_HiSeqV2_\
    PANCAN','PAM50Call_RNAseq',
                        'age_at_initial_pathologic_diagnosis')]
#save it uuid as a file
write.table(b$X_GENOMIC_ID_TCGA_BRCA_exp_HiSeqV2_PANCAN,file='PANCAN_uuid.\
    txt',quote=F,row.names=F,col.names=F)
#search for "TCGA Barcode to UUID Web Service User's Guide"
#get the corresponding barcode (input uuid/X_GENOMIC_ID_TCGA_BRCA_exp_\
    HiSeqV2_PANCAN) from metadata Browser
#https://tcga-data.nci.nih.gov/uuid/uuidBrowser.htm
barcode=read.csv('PAN_uuid_barcode_noemptyline.txt',sep='\t')
#keep uuid and barcode only for "barcode"
barcode=barcode[,c(1,2,7)]
colnames(barcode)[2]='PANCAN_barcode'
#change everything in barcode to lowercase to be consistent with "clinical"\
     data
barcode=data.frame(sapply(barcode,tolower))
#merge barcode data with orginal PAN clinical data
PANCAN=merge(b1,barcode,by.x='X_GENOMIC_ID_TCGA_BRCA_exp_HiSeqV2_PANCAN',by\
    .y='UUID')

#pick the column from "clinical data" containing "aliquot_uuid"
aliquot_uuid=clinical[,grep('aliquot_uuid',clinicalvar)]
aliuuidcol=colnames(aliquot_uuid)

#the aliquot with rinvalues (refer to log_16_02_05)
ali_uuid_rin=aliquot_uuid[,grep(paste("patient.samples.sample.2.portions.\
    portion.analytes.analyte.2",
                                      "patient.samples.sample.3.portions.\
    portion.analytes.analyte",
                                      "patient.samples.sample.4.portions.\
    portion.analytes.analyte.2",
                                      "patient.samples.sample.portions.\
    portion.analytes.analyte.2",
                                      "patient.samples.sample.portions.\
    portion.2.analytes.analyte",
                                      "patient.samples.sample.portions.\
    portion.3.analytes.analyte",
                                      "patient.samples.sample.portions.\
    portion.3.analytes.analyte",
                                      "patient.samples.sample.portions.\
    portion.analytes.analyte.5",
                                      sep="|"),aliuuidcol)]
```

```
#merge PANCAN and aliquot_uuid by uuid
totalnum=0
uuid_rin_col=c()
for (i in 1:ncol(ali_uuid_rin))
{
  assign(paste0('merge',i),
         merge(ali_uuid_rin[i],PANCAN,by.x=colnames(ali_uuid_rin)[i],by.y='\
    X_GENOMIC_ID_TCGA_BRCA_exp_HiSeqV2_PANCAN'))
  obs=nrow(get(paste0('merge',i)))
  totalnum=totalnum+obs
  print(totalnum)
  if (obs > 0)
  {
    uuid_rin_col=c(uuid_rin_col,colnames(ali_uuid_rin)[i])
  }
}
#we found the rinvalue for 1213 aliquot from TCGA clinical datasets out of \
    1215 data from PANCAN

#remove the merge* datasets generated first
rm(list=ls(pattern=glob2rx("merge*")))
#further subset ali_uuid_rin
ali_uuid_rin=ali_uuid_rin[,colnames(ali_uuid_rin)%in%uuid_rin_col]
totalnum=0
for (i in 1:ncol(ali_uuid_rin))
{
  assign(paste0('merge',i),
         merge(ali_uuid_rin[i],PANCAN,by.x=colnames(ali_uuid_rin)[i],by.y='\
    X_GENOMIC_ID_TCGA_BRCA_exp_HiSeqV2_PANCAN'))
  obs=nrow(get(paste0('merge',i)))
  totalnum=totalnum+obs
  print(totalnum)
}

#putting all the datasets together
rm(list=ls(pattern=glob2rx("merge*")))
#put "patient_barcode" into the dataframe
ali_uuid_rin=clinical[,colnames(clinical)%in%c("patient.bcr_patient_barcode\
    ",uuid_rin_col)]
totalnum=0
for (i in 2:ncol(ali_uuid_rin))
{
  new=cbind(ali_uuid_rin$patient.bcr_patient_barcode,ali_uuid_rin[i])
  colnames(new)[1]="patient.bcr_patient_barcode"
  assign(paste0('merge',i),
         merge(new,PANCAN,by.x=colnames(ali_uuid_rin)[i],by.y='X_GENOMIC_ID\
    _TCGA_BRCA_exp_HiSeqV2_PANCAN'))
  obs=nrow(get(paste0('merge',i)))
  totalnum=totalnum+obs
  print(totalnum)
}

#in the new* datasets, eliminate datasets containing "aliquots.aliquot.2", \
    "aliquots.aliquot.3", "aliquots.aliquot.4",
#"aliquots.aliquot.5"
#you can eliminates the same things in the orginal "clinical" dataset too
rm(list=ls(pattern=glob2rx("new*")))
for (i in 2:ncol(ali_uuid_rin))
```

```
{
  uuid=colnames(get(paste0('merge',i)))[1]
  aliquot=unlist(strsplit(uuid,'.bcr_aliquot_uuid'))
  rinvalue=paste0(unlist(strsplit(aliquot,'aliquots.aliquot'))[1],'rna.\
    rinvalue')
  s18s28=paste0(unlist(strsplit(aliquot,'aliquots.aliquot'))[1],'rna.ratio_\
    28s_18s')
  yearofcre=paste0(unlist(strsplit(aliquot,'analytes.analyte'))[1],'year_of\
    _creation')
  aliquot.col=grep(aliquot,clinicalvar,ignore.case=T,value=T)
  #note that in this step, it takes the end of the colnames no matter which\
     aliquot it belongs
  #therefore, in the newi dataset being generated, if there are multiple \
    aliquots found
  #we should take the one without numbers (eg. aliquot.2, aliquot.3 or \
    aliquot.4)
  aliquot.col.end=sapply(strsplit(aliquot.col,paste0(aliquot,'[.]')),\
    function(x) x[[2]])

  test=clinical[,grep(aliquot,clinicalvar)]
  test=data.frame(test,clinical[,rinvalue],clinical[,s18s28],clinical[,\
    yearofcre])
  colnames(test)=aliquot.col.end
  colnames(test)[ncol(test)-2]='rinvalue'
  colnames(test)[ncol(test)-1]='rna.ratio_28s_18s'
  colnames(test)[ncol(test)]='year_of_creation'

  assign(paste0('new',i),merge(test,get(paste0('merge',i)),by.x="bcr_\
    aliquot_uuid",by.y=uuid))
  print(paste0('new',i))
  #print out the number of columns in the "newi" dataset
  #print(ncol(get(paste0('new',i))))

  #make sure the barcode and uuid matches, and pick up the correct aliquot
  #check out whether the barcode from the clinical the same as the one from\
     PANCAN
  #we will check the .aliquot only (not 2.aliquot, 3.aliquot or 4.aliquot)
  print('Is it true that the barcodes are consitent?')
  print(all(get(paste0('new',i))$bar_aliquot_barcde,get(paste0('new',i))$\
    PANCAN_barcode,ignoreNames=T))

  #eliminate any columns starting with number/additional studies conducted \
    or not/source_center/canonical_reason
  numcol=glob2rx(paste("2.*","3.*","4.*","5.*","6.*","7.*","8.*","\
    additional_studies*","*canonical_reason",
                        "source_center",sep="|"))
  dataset=get(paste0('new',i))
  assign(paste0('new',i),dataset[,grep(numcol,colnames(dataset),invert=T)])
  print(ncol(get(paste0('new',i))))
}

aliquot_specific=data.frame()
for (i in 2:ncol(ali_uuid_rin))
{
  aliquot_specific=rbind(aliquot_specific,get(paste0('new',i)))
}

#grep other meaningful varaible from the "clinical" dataset
list=paste("patient.bcr_patient_barcode",
```

```
            "batch",
            "days_to_birth","ethnicity","race","country","histological",
            "margin","menopause","gender",sep='|')
newclinical=clinical[,grep(list,clinicalvar)]

#merge aliquot_specific and newclinical together
final=merge(aliquot_specific,newclinical,by='patient.bcr_patient_barcode',\
    all.x=T)
final=final[,grep('PANCAN_barcode',colnames(final),invert=T)]

#add in the population structure
pop=read.table("<dir>/population_structure/TCGA_BrCa_all_chr_merged_\
    filtered.cluster2")
colnames(pop)=c('ID','ID2','population')
pop$ID=as.character(pop$ID)
#split on "-" and produce 7 separate column
pop$project=sapply(strsplit(pop$ID,'-'),function(x) x[[1]])
table(pop$project)
pop$TSS=sapply(strsplit(pop$ID,'-'),function(x) x[[2]])
table(pop$TSS)#add this as a covariant
pop$person=sapply(strsplit(pop$ID,'-'),function(x) x[[3]])

#create the "patient.bcr_patient_barcode" column
pop$patient.bcr_patient_barcode=paste(pop$project,pop$TSS,pop$person,sep='-\
    ')
#change it to lowercase to be consisent with "final" datasets
pop$patient.bcr_patient_barcode=sapply(pop$patient.bcr_patient_barcode,\
    tolower)
pop=pop[,c(7,3,5)]
#remove duplicated rows and pick one randomly
pop=pop[!duplicated(pop$patient.bcr_patient_barcode),]

#merge "final" and "pop" dataset
final.pop=merge(final,pop,by='patient.bcr_patient_barcode',all.x=T)

#go over each column of "final.pop" and eliminate the columns with lots of \
    missing values
for (i in 1:ncol(final.pop))
{
  print(paste('col',i))
  print(colnames(final.pop)[i])
  print(sum(is.na(final.pop[,i])))
}
final.pop=final.pop[,-c(3,25,26,32,36,37,39,40)]

rownames(final.pop)=final.pop$sampleID
#remove covs with duplicated barcode/uuid
final.pop=final.pop[!names(final.pop)%in%c("bcr_aliquot_uuid","bcr_aliquot_\
    barcode",
                                            "biospecimen_barcode_bottom","\
    sampleID")]
#remove factor covs with only one level
#remove "age_at_initial_pathologic_diagnosis" which is highly correlated \
    with "patient.days_to_birth"
#remove duplicated "patient.clinical_cqcf.histological_type","patient.\
    biospecimen_cqcf.histological_type" with "patient.histological_type"
final.pop=final.pop[!names(final.pop)%in%c("center_id","is_derived_from_\
    ffpe",
                                            "age_at_initial_pathologic_\
```

```
    diagnosis",
                                                "patient.clinical_cqcf.\
    histological_type","patient.biospecimen_cqcf.histological_type")]
#merge the shipment of year/month/day together
final.pop=within(final.pop,
                 {shipment=as.factor(paste0("'",month_of_shipment,day_of_\
    shipment,year_of_creation,"'"))}
)
final.pop=final.pop[,grep('_of_shipment',colnames(final.pop),invert=T)]

lapply(final.pop,class)#
final.pop.1=within(final.pop,
                   {
                     concentration=as.numeric(as.character(concentration))
                     quantity=as.numeric(as.character(quantity))
                     volume=as.numeric(as.character(volume))
                     rinvalue=as.numeric(as.character(rinvalue))
                     rna.ratio_28s_18s=as.numeric(as.character(rna.ratio_28\
    s_18s))
                     patient.days_to_birth=as.numeric(as.character(patient.\
    days_to_birth))
                     TSS=as.factor(TSS)
                     #year_of_creation=as.numeric(as.factor(year_of_\
    creation))
                   }
)

cov.class=lapply(final.pop.1,class)#double check the new class
#save the "factor" covariants in a character vector
cov.factor=character()
for (i in 1:length(cov.class))
{
  cov.name=names(cov.class[i])
  print(names(cov.class[i]))
  class=cov.class[[i]]
  print(cov.class[[i]])
  if (identical(class,'factor'))
  {cov.factor=c(cov.factor,cov.name)}
}

fit=princomp(na.omit(final.pop.1[!names(final.pop.1)%in%cov.factor]),cor=T)
plot(fit,type='lines')
fitscore=data.frame(fit$scores)
final.pop.1.pca=merge(fitscore[,c(1:3)],final.pop.1[names(final.pop.1)%in%\
    cov.factor],by='row.names')

final.pop.1.pca.tumor=subset(final.pop.1.pca,final.pop.1.pca$Sample.Type=='\
    primary solid tumor')
rownames(final.pop.1.pca.tumor)=toupper(gsub('-','.',final.pop.1.pca.tumor$\
    patient.bcr_patient_barcode))
covpatient=rownames(final.pop.1.pca.tumor)
cov=final.pop.1.pca.tumor
cov=cov[!names(cov)%in%c("Row.names","patient.bcr_patient_barcode","Sample.\
    Type")]

cov=data.frame(t(cov))
newcov=cbind(rownames(cov),cov)
colnames(newcov)[1]='id'
write.table(newcov,file='covariant.txt',quote=F,sep='\t',row.names=F)
```

```
system("gzip covariant.txt")
#in terminal
#gzip covariant.txt
#end of this session, keep the patients corresponding to this covariants/\
    phenotype files
rm(list=setdiff(ls(), c("phenopatient","covpatient")))

####create_genotype_file########
#read in the list of corordiantes, chromosome number, and snp name from the\
     file.
automate=read.csv('<dir>/mast1e-4_ic0.5_automate_spreadsheet.txt',
                    sep="\t")

#note that this is the start coordinate from the hg38!!!
#note that this is from the hg19 coordinates
cor=automate$HG19.START.COR
chr=sapply(strsplit(as.character(automate$CHR),"chr"),function(x) x[[2]])
snp=automate$LD.SNP

for (i in 1:length(snp))
{
  #create*_filter.recode.vcf in bash script
  #bash <dir>/tcga_patients_genotype_data.sh
  #this step choose coordinates within 1MB from the imputed vcf files and \
    use the following filter:
  #--maf 0.05
  #--max-alleles 2
  #--hwe 1e-06
  file=paste0('chr',chr[i],'_',snp[i],'_filter.recode.vcf')
  tail=paste('tail','-n+10',file,'>','newgenotype.vcf')
  #create "newgenotype.vcf" file
  system(tail)

  genotype=read.table('newgenotype.vcf',header=T,comment.char="")
  system("rm newgenotype.vcf")
  t.genotype=data.frame(t(genotype))
  t.genotype$ID=rownames(t.genotype)
  t.genotype$newID=sapply(strsplit(t.genotype$ID,split="[.]10A"),function(x\
    ) x[[1]])
  t.genotype=subset(t.genotype,t.genotype$newID!=c("TCGA.E2.A10A.01A.21D.\
    A111.01"))
  t.genotype=t.genotype[!duplicated(t.genotype$newID),]
  rownames(t.genotype)=t.genotype$newID
  t.genotype.sub=t.genotype[,!colnames(t.genotype)%in%c('ID','newID')]
  newgenotype=data.frame(t(t.genotype.sub))
  colnames(newgenotype)[1]='#CHROM'
  genopatient=colnames(newgenotype)[-c(1:9)]
  write.table(newgenotype,file=paste0('chr',chr[i],'_',snp[i],'_','genotype\
    .vcf'),quote=F,row.names=F,sep='\t')

  ########sample_to_be_exclude######
  inter=intersect(covpatient,intersect(genopatient,phenopatient))
  all=union(covpatient,union(genopatient,phenopatient))
  sample_to_exclude=all[!all%in%inter]
  exclude=data.frame(sample_to_exclude)
  write.table(exclude,file=paste0('chr',chr[i],'_',snp[i],'_','sample_\
    exclude.exc'),quote=F,sep='\t',row.names=F,col.names=F)
}
#end of this session
```

```
rm(list=ls())

system("bash <dir>/fastQTL_geno_bgzip.sh")
#run in terminal
#bash <dir>/fastQTL_geno_bgzip.sh

#######fastQTL#########
system("bash <dir>/fastQTL.sh")
#run everything in bash script
#bash <dir>/fastQTL.sh

######fastQTL_result_visualization########
rm(list=ls())
#this is hg19 cordiantes?
#read in the list of corordiantes, chromosome number, and snp name from the\
     file.
automate=read.csv('<dir>/mast1e-4_ic0.5_automate_spreadsheet.txt',
                  sep="\t")

#make a new folder for the visualization results
system("mkdir eQTL_visualization")

#note that this is the start coordinate from the hg38!!!
#note that this is from the hg19 coordinates
cor=automate$HG19.START.COR
chr=sapply(strsplit(as.character(automate$CHR),"chr"),function(x) x[[2]])
snps=as.character(automate$LD.SNP)
#the length of unique SNPs
length(unique(snps))#463
#save all the significant snp info. in a new variable
snpinfo=data.frame()
filename='1mb_snps.txt'
snplocname=read.csv(filename,sep='\t')
snplocname=snplocname[,c(3,4)]
save(snplocname,file='1mb_snps.RData')
#load("1mb_snps.RData")

for (i in c(1:length(snps)))
{
  print(i)
  ciseqtl=read.csv(paste0('chr',chr[i],'_',snps[i],'_',"nominals.cov.txt"),\
    sep=' ',header=F)
  ciseqtl$cor=sapply(strsplit(as.character(ciseqtl[,2]),paste0(chr[i],':'))\
    ,function(x) x[[2]])
  snplocname.sub=snplocname[!duplicated(snplocname[,1]),]
  colnames(snplocname.sub)=c('cor','snp')
  newciseqtl=merge(ciseqtl,snplocname.sub,by='cor')
  colnames(newciseqtl)=c('cor','gene','chrcor','dis','p.value','slope','snp\
    ')
  newciseqtl=newciseqtl[order(newciseqtl$p.value),]
  #newciseqtl=newciseqtl[,c(1,2,5,7)]#recent
  newciseqtl=newciseqtl[,c(1,2,3,5,7)]
  #keep p.value < e-5 in newciseqtl
  newciseqtl=newciseqtl[newciseqtl$p.value < 1e-5,]
  #or keep p.value < e-4 in newciseqtl
  #newciseqtl=newciseqtl[newciseqtl$p.value < 1e-4,]

  library(ggrepel)
```

```
  eqtl=newciseqtl
  genelist=unique(na.omit(as.character(eqtl$gene[eqtl$snp==snps[i]])))
  if (length(genelist) > 0)
  {

    for (j in (1:length(genelist)))
    {
      gene=subset(eqtl,eqtl$gene==unlist(genelist[j]))
      gene$newcor=gene$cor
      gene$newcor=as.numeric(as.character(gene$newcor))
      gene$newp=-log10(gene$p.value)
      snp=subset(gene,snp==snps[i])
      #save all the significant snp info. in a new variable called sninfo
      snpinfo=rbind(snpinfo,snp)
      topsnp=gene[1:10,]
      pdf(paste0('eQTL_visualization/','chr',chr[i],'_',snps[i],'_',\
   genelist[j],'.pdf'),width=8,height=4)
      print(
        ggplot(data=gene,aes(x=newcor,y=newp))+
          geom_point(colour='hotpink',size=1)+
          geom_point(data=snp,aes(x=newcor,y=newp),colour="blue",size=4)+
          geom_text_repel(data=snp,aes(x=newcor,y=newp),label=snp$snp,\
   colour="blue",size=4)+
          geom_text_repel(data=topsnp,aes(x=newcor,y=newp),label=topsnp$snp\
   ,colour="blue",size=4)+
          xlab(paste('chr',chr[i],'coordinates'))+
          ylab('-log10(P.value)')+
          ggtitle(unlist(genelist[j]))+
          scale_x_continuous(breaks=seq(min(gene$newcor),max(gene$newcor),\
   by=(max(gene$newcor)-min(gene$newcor))/5))+
          theme_bw(base_size=15)+
          theme(panel.grid.major = element_blank(),
                panel.grid.minor = element_blank())
      )
      dev.off()
    }
  }
  }

write.table(snpinfo,file='eQTL_visualization/eqtl_snpinfo.txt',quote=F,sep=\
   '\t',row.names=F)

###prepare doc for locus zoom plots######
rm(list=ls())
setwd("<dir>")
load("1mb_snps.RData")

automate=read.csv('eQTL_visualization/eqtl_snpinfo.txt',
                  sep="\t")

#note that this is the start coordinate from the hg38!!!
#note that this is from the hg19 coordinates
cor=automate$cor
chr=sapply(strsplit(as.character(automate$chrcor),":"),function(x) x[[1]])
snps=as.character(automate$snp)
genelist=as.character(automate$gene)

for (i in 1:length(snps))
{
```

```
ciseqtl=read.csv(paste0('chr',chr[i],'_',snps[i],'_',"nominals.cov.txt"),\
    sep=' ',header=F)
ciseqtl$cor=sapply(strsplit(as.character(ciseqtl[,2]),paste0(chr[i],':')),\
    function(x) x[[2]])
snplocname.sub=snplocname[!duplicated(snplocname[,1]),]
colnames(snplocname.sub)=c('cor','snp')
newciseqtl=merge(ciseqtl,snplocname.sub,by='cor')
colnames(newciseqtl)=c('cor','gene','chrcor','dis','p.value','slope','snp')
newciseqtl=newciseqtl[order(newciseqtl$p.value),]
newciseqtl=newciseqtl[,c(1,2,5,7)]
eqtl=newciseqtl
gene=subset(eqtl,eqtl$gene==genelist[i])
locuszoom=gene[,c(4,3)]
colnames(locuszoom)=c('MarkerName','P.value')
write.table(locuszoom,file=paste0('eQTL_visualization/','chr',chr[i],'_',\
    snps[i],'_',genelist[i],'_',
                                        'locuszoom.txt'),quote=F,sep='\t',row.\
    names=F,col.names=T)
}
```

U Bash shell script: zip and index the genotype file for eQTL analysis

```
#!/usr/local/Cellar/bash/4.3.42/bin/bash
#run the bash script locally
#bash <dir>/fastQTL_geno_bgzip.sh
start=`date +%s`
dir=<dir>
automate=<dir>/mast1e-4_ic0.5_automate_spreadsheet.txt

cd $dir

#read in the chromosome, cordinates, and snp name information from the file
readarray -t chrarray < <(cut -f1 $automate | tail -n+2)
readarray -t snparray < <(cut -f7 $automate | tail -n+2)

i=0
for chr in "${chrarray[@]}"
do
    echo $chr
#added here
    chrnum=${chr#chr}
    echo $chrnum
    snp=${snparray[$i]}
    echo $snp
    bgzip chr${chrnum}_${snp}_genotype.vcf && tabix -p vcf chr${chrnum}_${\
   snp}_genotype.vcf.gz

    (( i = i +1 ))
done
end=`date +%s`
runtime=$((end-start))
echo runtime:$runtime
```

V Bash shell script: eQTL analysis by fastQTL

```
#!/usr/local/Cellar/bash/4.3.42/bin/bash
#run the bash script locally
#bash <dir>/fastQTL.sh
start=`date +%s`
dir=<dir>
automate=<dir>/mast1e-4_ic0.5_automate_spreadsheet.txt

cd $dir

#read in the chromosome, cordinates, and snp name information from the file
readarray -t chrarray < <(cut -f1 $automate | tail -n+2)
#those cordinates are in hg19
readarray -t corarray < <(cut -f5 $automate | tail -n+2)
readarray -t snparray < <(cut -f7 $automate | tail -n+2)

i=0
for chr in "${chrarray[@]}"
do
    echo chr:$chr
#newly added command
    chrnum=${chr#chr}
    echo $chrnum
    snp=${snparray[$i]}
    echo snp:$snp
    cor=${corarray[$i]}
    echo cor:$cor
    mb=1000000

    firstcor=$(($cor-$mb))
    echo firstcor:$firstcor
    lastcor=$(($cor+$mb))
    echo lastcor:$lastcor

    fastQTL --vcf chr${chrnum}_${snp}_genotype.vcf.gz --bed phenotype.bed.\
   gz --region $chrnum:$firstcor-$lastcor --threshold 0.1 --exclude-samples\
    chr${chrnum}_${snp}_sample_exclude.exc --cov covariant.txt.gz --out \
   chr${chrnum}_${snp}_nominals.cov.txt.gz --log chr${chrnum}_${snp}\
   _fastQTL.log
    gunzip chr${chrnum}_${snp}_nominals.cov.txt.gz

    (( i = i +1 ))
done
end=`date +%s`
runtime=$((end-start))
echo runtime:$runtime

#note that due to the same candidate causal SNP could be in LD with \
   multiple GWAS BrCA SNPs, it is possible that the output chr${chrnum}_${\
   snp}_nominals.cov.txt.gz already exist. Just type in "y" if you ever see\
    "chr${chrnum}_${snp}_nominals.cov.txt.gz already exists -- do you wish \
   to overwrite (y or n)?"
```

References

Ardlie KG, Deluca DS, Segrè AV, Sullivan TJ, Young TR, Gelfand ET, Trowbridge CA, Maller JB, Tukiainen T, Lek M, *et al.* (2015). "The Genotype-Tissue Expression (GTEx) pilot analysis: Multitissue gene regulation in humans." *Science*, **348**(6235), 648–660.

Bailey TL (2011). "DREME: motif discovery in transcription factor ChIP-seq data." *Bioinformatics*, **27**(12), 1653–1659.

Bailey TL, Elkan C, *et al.* (1994). "Fitting a mixture model by expectation maximization to discover motifs in bipolymers."

Bailey TL, Gribskov M (1998). "Combining evidence using p-values: application to sequence homology searches." *Bioinformatics*, **14**(1), 48–54.

Benjamini Y, Hochberg Y (1995). "Controlling the false discovery rate: a practical and powerful approach to multiple testing." *Journal of the Royal Statistical Society. Series B (Methodological)*, pp. 289–300.

Boyle AP, Davis S, Shulha HP, Meltzer P, Margulies EH, Weng Z, Furey TS, Crawford GE (2008). "High-resolution mapping and characterization of open chromatin across the genome." *Cell*, **132**(2), 311–322.

Clauset A, Newman ME, Moore C (2004). "Finding community structure in very large networks." *Physical review E*, **70**(6), 066111.

Consortium G, *et al.* (2015). "The Genotype-Tissue Expression (GTEx) pilot analysis: Multitissue gene regulation in humans." *Science*, **348**(6235), 648–660.

Danecek P, Auton A, Abecasis G, Albers CA, Banks E, DePristo MA, Handsaker RE, Lunter G, Marth GT, Sherry ST, *et al.* (2011). "The variant call format and VCFtools." *Bioinformatics*, **27**(15), 2156–2158.

Goldman M, Craft B, Swatloski T, Cline M, Morozova O, Diekhans M, Haussler D, Zhu J (2014). "The UCSC Cancer Genomics Browser: update 2015." *Nucleic acids research*, p. gku1073.

Gupta S, Stamatoyannopoulos JA, Bailey TL, Noble WS (2007). "Quantifying similarity between motifs." *Genome biology*, **8**(2), R24.

John S, Sabo PJ, Thurman RE, Sung MH, Biddie SC, Johnson TA, Hager GL, Stamatoyannopoulos JA (2011). "Chromatin accessibility pre-determines glucocorticoid receptor binding patterns." *Nature genetics*, **43**(3), 264–268.

Langmead B, Salzberg SL (2012). "Fast gapped-read alignment with Bowtie 2." *Nature methods*, **9**(4), 357–359.

Lazarovici A, Zhou T, Shafer A, Machado ACD, Riley TR, Sandstrom R, Sabo PJ, Lu Y, Rohs R, Stamatoyannopoulos JA, *et al.* (2013). "Probing DNA shape and methylation state on a genomic scale with DNase I." *Proceedings of the National Academy of Sciences*, **110**(16), 6376–6381.

Li H, Handsaker B, Wysoker A, Fennell T, Ruan J, Homer N, Marth G, Abecasis G, Durbin R, *et al.* (2009). "The sequence alignment/map format and SAMtools." *Bioinformatics*, **25**(16), 2078–2079.

Ma W, Noble WS, Bailey TL (2014). "Motif-based analysis of large nucleotide data sets using MEME-ChIP." *Nature protocols*, **9**(6), 1428–1450.

Ongen H, Buil A, Brown AA, Dermitzakis ET, Delaneau O (2015). "Fast and efficient QTL mapper for thousands of molecular phenotypes." *Bioinformatics*, p. btv722.

Pollard KS, Hubisz MJ, Rosenbloom KR, Siepel A (2010). "Detection of nonneutral substitution rates on mammalian phylogenies." *Genome research*, **20**(1), 110–121.

Pruim RJ, Welch RP, Sanna S, Teslovich TM, Chines PS, Gliedt TP, Boehnke M, Abecasis GR, Willer CJ (2010). "LocusZoom: regional visualization of genome-wide association scan results." *Bioinformatics*, **26**(18), 2336–2337.

Siepel A, Bejerano G, Pedersen JS, Hinrichs AS, Hou M, Rosenbloom K, Clawson H, Spieth J, Hillier LW, Richards S, *et al.* (2005). "Evolutionarily conserved elements in vertebrate, insect, worm, and yeast genomes." *Genome research*, **15**(8), 1034–1050.

Sung MH, Guertin MJ, Baek S, Hager GL (2014). "DNase footprint signatures are dictated by factor dynamics and DNA sequence." *Molecular cell*, **56**(2), 275–285.
