# Supplementary figures and images for: Identification of breast cancer associated variants that modulate transcription factor binding
Source: PLoS Genet. 2017 Sep 28;13(9):e1006761. doi: 10.1371/journal.pgen.1006761 (PMC5619690; doi:10.1371/journal.pgen.1006761)

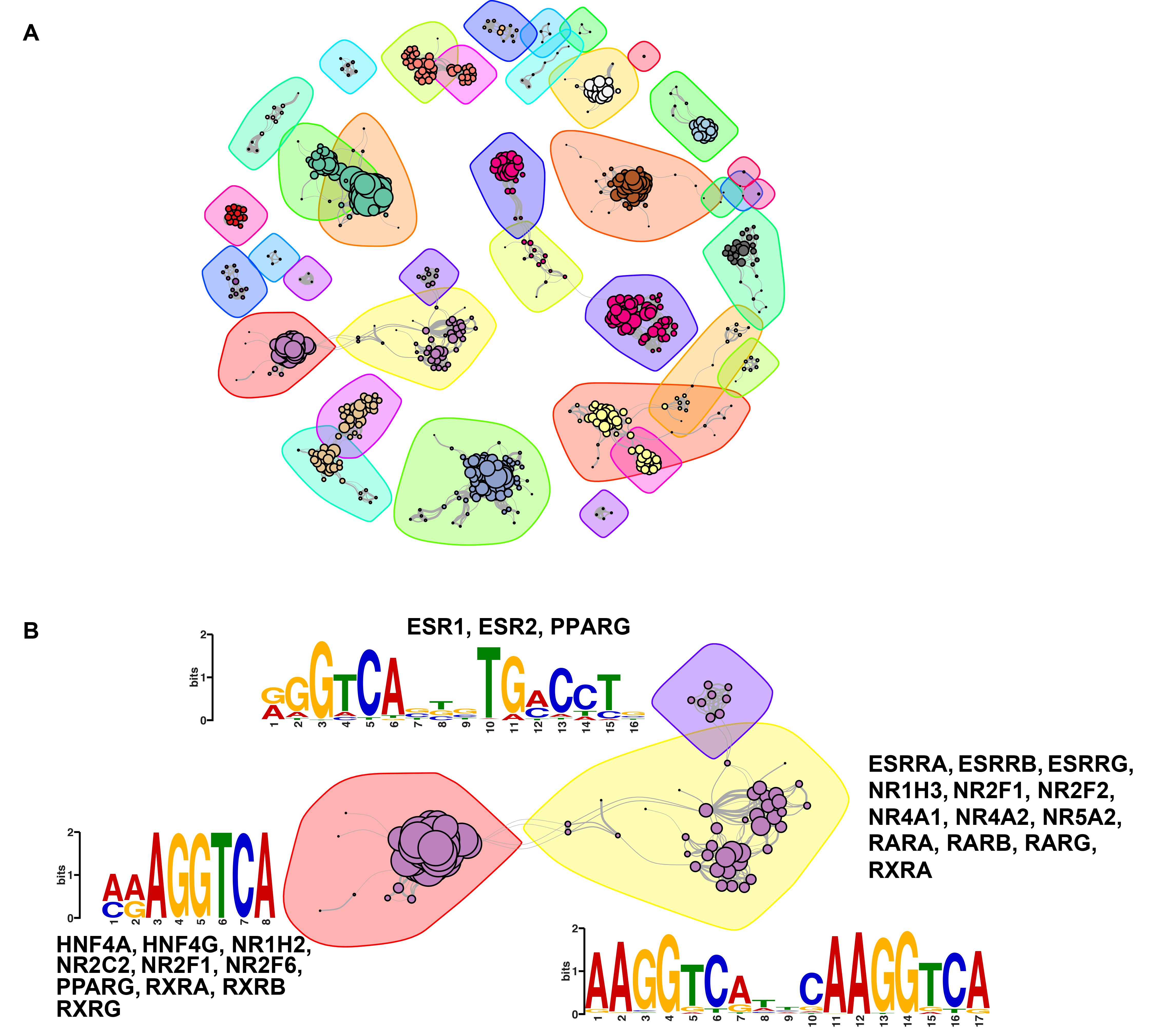

Supplement: S1 Fig — (A) We clustered several publicly available PSWMs into families based on their similarities. In this illustration each node is a PSWM and the size of each node is proportional its number of edges. Contiguously connected nodes are colored uniquely and each family of PSWM is outlined by a distinct background color. We inferred edges using TOMTOM [75] to match PSWMs. The width of edges denote the similarity between two PSWMs and this width is proportional to the −log10 E-value of the match. (B) TFs are organized into families that recognize similar sequences and a representative motif for each regulatory sequence family is presented as a seqLogo. For example, three known TFs (ESR1, ESR2, and PPARG-γ) recognize the estrogen response element (ERE). (TIF) [file pgen.1006761.s003.tif]

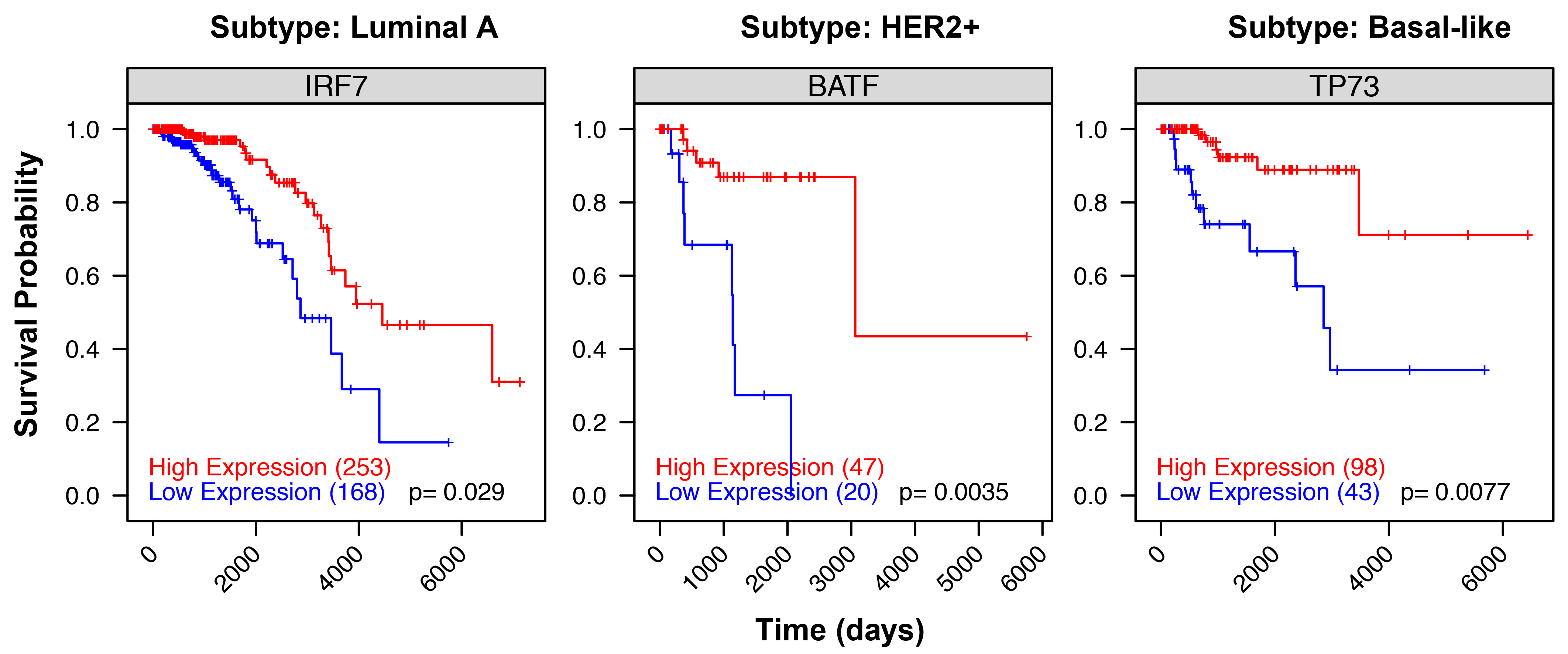

Supplement: S3 Fig — High expression of IRF7, BATF, and TP73 is correlated with better breast cancer patient outcome in Luminal A, HER2+, and Basal-like subtype, respectively. TF expression groups are classified as either high or low expression and with the corresponding patient number is noted in the parentheses. We use FDR to correct the P-values for multiple testing. Kaplan-Meier analysis is performed using TCGA breast cancer patient solid tumor sample RNA-seq expression and overall breast cancer patient survival data [57]. (TIF) [file pgen.1006761.s005.tif]

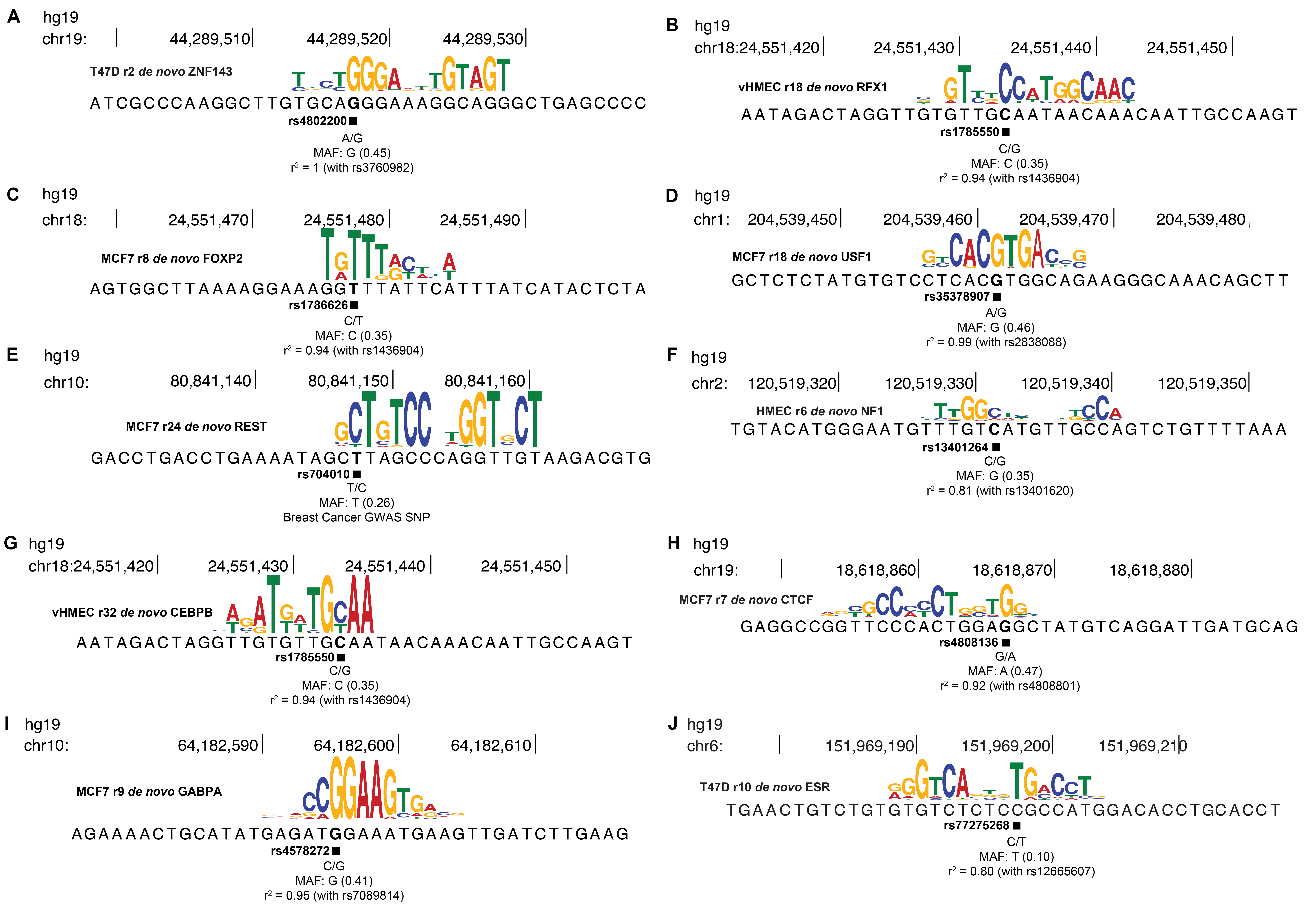

Supplement: S4 Fig — Each SNP is in strong LD (r2 ≥ 0.8) with the most associated breast cancer GWAS SNP and affects TF binding affinity. We report the minor allele frequency (MAF) and LD association (r2) of each SNP with the most associated breast cancer GWAS SNP. (TIF) [file pgen.1006761.s006.tif]

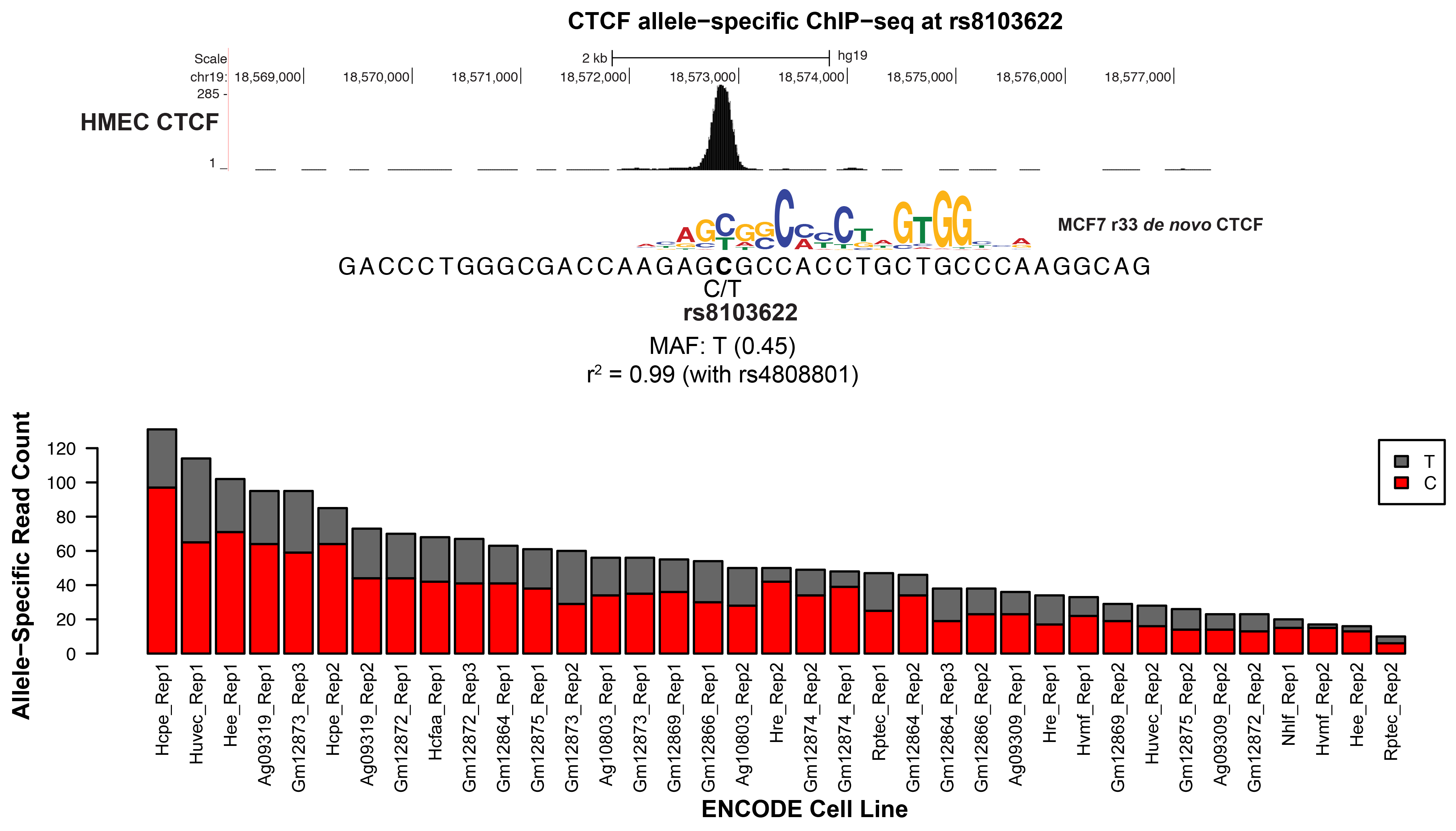

Supplement: S5 Fig — ENCODE ChIP-seq data (top) indicates that CTCF is strongly bound in HMEC at rs8103622. The risk allele C of rs8103622 is predicted to increase CTCF binding, which was identified in round 33 of our de novo motif analysis from MCF7 (middle). By analyzing ENCODE ChIP-seq count data, we show that there is an allelic imbalance favoring C (denoted in red) versus T (denoted in black) in 34 out of 37 cell types/replicates that are diploid and heterozygous for the C/T allele at rs8103622. (TIF) [file pgen.1006761.s007.tif]
